# Roles of Glutathione and AP-1 in the Enhancement of Vitamin D-Induced Differentiation by Activators of the Nrf2 Signaling Pathway in Acute Myeloid Leukemia Cells

**DOI:** 10.3390/ijms25042284

**Published:** 2024-02-14

**Authors:** Yasmeen Jramne-Saleem, Michael Danilenko

**Affiliations:** Department of Clinical Biochemistry and Pharmacology, Faculty of Health Sciences, Ben-Gurion University of the Negev, Beer Sheva 8410501, Israel; jramne@post.bgu.ac.il

**Keywords:** acute myeloid leukemia, buthionine sulfoximine, carnosic acid, monomethyl fumarate, vitamin D receptor, activator protein-1

## Abstract

Active vitamin D derivatives (VDDs)—1α,25-dihydroxyvitamin D_3_/D_2_ and their synthetic analogs—are well-known inducers of cell maturation with the potential for differentiation therapy of acute myeloid leukemia (AML). However, their dose-limiting calcemic activity is a significant obstacle to using VDDs as an anticancer treatment. We have shown that different activators of the NF-E2-related factor-2/Antioxidant Response Element (Nrf2/ARE) signaling pathway, such as the phenolic antioxidant carnosic acid (CA) or the multiple sclerosis drug monomethyl fumarate (MMF), synergistically enhance the antileukemic effects of various VDDs applied at low concentrations in vitro and in vivo. This study aimed to investigate whether glutathione, the major cellular antioxidant and the product of the Nrf2/ARE pathway, can mediate the Nrf2-dependent differentiation-enhancing activity of CA and MMF in HL60 human AML cells. We report that glutathione depletion using L-buthionine sulfoximine attenuated the enhancing effects of both Nrf2 activators concomitant with downregulating vitamin D receptor (VDR) target genes and the activator protein-1 (AP-1) family protein c-Jun levels and phosphorylation. On the other hand, adding reduced glutathione ethyl ester to dominant negative Nrf2-expressing cells restored both the suppressed differentiation responses and the downregulated expression of VDR protein, VDR target genes, as well as c-Jun and P-c-Jun levels. Finally, using the transcription factor decoy strategy, we demonstrated that AP-1 is necessary for the enhancement by CA and MMF of 1α,25-dihydroxyvitamin D_3_-induced VDR and RXRα protein expression, transactivation of the vitamin D response element, and cell differentiation. Collectively, our findings suggest that glutathione mediates, at least in part, the potentiating effect of Nrf2 activators on VDDs-induced differentiation of AML cells, likely through the positive regulation of AP-1.

## 1. Introduction

Acute myeloid leukemia (AML) is one of the most aggressive hematologic malignancies, primarily targeting older adults aged ≥65. It is characterized by genetic and epigenetic defects that block the development of myeloid progenitor cells in the bone marrow at early stages of differentiation and promotes the uncontrolled growth of leukemic blasts. Combination chemotherapy with cytosine β-D-arabinofuranoside (cytarabine) and an anthracycline antibiotic (e.g., daunorubicin) has been the frontline treatment for AML for more than 40 years and is relatively successful for younger patients. However, older individuals are mainly unfit for intensive chemotherapy and their treatment options remain sparse, resulting in a very short median overall survival (6–25 months) [1,2]. Although several novel targeted AML drugs are currently available, their impact on long-term patient survival is yet to be determined [1,3,4]. Since maturation block is the primary feature of AML blasts, differentiation therapy presents an attractive alternative option for treating this disease [5]. One AML subtype, acute promyelocytic leukemia (APL), has been successfully treated with the combination of the natural differentiation inducer all-*trans*-retinoic acid (ATRA) and arsenic trioxide [6]. No differentiation therapy is currently available for nonAPL AML. However, recent studies have demonstrated differentiation-related clinical responses to specific inhibitors of mutant dehydrogenase 1 (IDH1) and IDH2 in AML patients carrying these mutations [7,8].

The hormonal form of vitamins D_3_ and D_2_, 1α,25-dihydroxyvitamin D_3_/D_2_ (1,25D_3/2_), is a well-known inducer of myeloid differentiation in various nonAPL AML cell types [9,10]. 1,25D_3_ is the physiological ligand of the vitamin D receptor (VDR), a member of the nuclear receptor subfamily type II that includes retinoid X receptors (RXRs) and retinoic acid receptors. Upon binding 1,25D_3_, VDR interacts with RXRα and/or other transcription factors, such as purine-rich box-1 (PU.1) or CCAAT enhancer binding protein alpha (CEBPα), to form protein complexes that act as ligand-activated transcription factors [11,12].

A major obstacle to the clinical development of vitamin D derivatives (VDDs)—1,25D_3/2_ and their synthetic analogs—for AML therapy is their dose-limiting calcemic toxicity. Clinical trials of different VDDs conducted so far have reported low anticancer efficiency at safe plasma levels of the compounds [9,10,13]. Furthermore, since some AML subtypes showed resistance to VDDs in ex vivo studies, only those patients who are likely to respond would probably benefit from VDD-based differentiation therapy [9]. A possible way of managing VDD toxicity is combining these compounds at tolerated doses with other agents that would potentiate their anticancer effect but not the calcemic activity.

We and others have shown that different plant antioxidants, such as carotenoids [14,15] and polyphenols, e.g., carnosic acid (CA), silibinin and curcumin [14,16,17,18,19,20], can synergistically enhance the differentiation-inducing and antiproliferative effects of various VDDs applied at low (sub)nanomolar concentrations on human and murine AML cell lines. Similar cooperative prodifferentiation effects of 1,25D_3_ and polyphenolic antioxidants were also obtained in patient-derived AML blasts [21,22]. Notably, combined treatment with CA-rich rosemary extract and low-calcemic VDDs resulted in cooperative antileukemic effects in syngeneic mouse models of AML without inducing hypercalcemia [18,23]. The VDD/CA-induced differentiation was associated with lowered intracellular levels of reactive oxygen species (ROS), upregulated expression of antioxidant enzymes, such as NAD(P)H quinone oxidoreductase-1 (NQO1) and the rate-limiting glutathione-synthesizing enzyme γ-glutamylcysteine synthetase (γGCS), and increased total glutathione content in AML cells [22,23,24]. On the other hand, depletion of cellular glutathione reduced the extent of differentiation [24].

These findings suggested the role of redox-related mechanisms in the differentiation-enhancing effects of polyphenols. Indeed, we have shown that the CA enhancement is mediated via activation of the nuclear factor erythroid 2-related factor 2 (Nrf2) transcription factor [22], a major regulator of the cytoprotective response to electrophilic agents and oxidative stress [25,26]. This was demonstrated by manipulating Nrf2 activity and expression in U937 human AML cells stably expressing a dominant-negative Nrf2 mutant (dnNrf2) lacking the transactivation domain and those overexpressing the wild-type Nrf2 [22]. Further, we found that besides CA, other structurally distinct Nrf2 activators, including the multiple sclerosis drugs dimethyl fumarate (DMF) and monomethyl fumarate (MMF) [27], synergistically potentiated the antileukemic effects of several VDDs on different AML cell types [28]. Notably, combined treatment with DMF and the highly potent low-calcemic vitamin D_2_ analog PRI-5202 cooperatively inhibited leukemia progression in a xenograft model of AML [28].

The cooperation between VDDs and Nrf2 activators was associated with a mutual upregulation of VDR, RXRα, and Nrf2 protein levels and activation of VDR and Nrf2 signaling [22,28]. Other transcriptional pathways are likely to contribute to this synergy. For instance, we have demonstrated a cooperative upregulation of several activator protein 1 (AP-1) family members and augmented DNA binding and transcriptional activity of AP-1 [17,22,29]. A marked upregulation of the early growth response protein 1 (EGR-1) transcription factor was also observed [17,29]. Stable expression of the wild-type Nrf2 or dnNrf2 in U937 cells resulted in an enhanced or reduced AP-1 upregulation and activation, respectively [22], suggesting that Nrf2 may serve as an upstream regulator of AP-1 in AML cells.

The present study was designed to investigate whether glutathione, the most abundant cellular antioxidant and the product of the Nrf2/antioxidant response element (Nrf2/ARE) signaling pathway [26], may mediate the enhancing effects of Nrf2 activators on VDD-induced differentiation of AML cells. To this end, we employed two approaches: (1) glutathione depletion in nontransfected HL60 cells using L-buthionine sulfoximine (BSO), a specific and irreversible γGCS inhibitor, and (2) repletion of reduced glutathione (GSH) levels in dnNrf2-expressing HL60 cells by adding membrane-permeant GSH ethyl ester (GEE). We found that co-treatment with BSO attenuated the potentiating effect of both CA and MMF on 1,25D_3_-induced differentiation. This was paralleled by the downregulation of VDR-responsive genes, the AP-1 family protein c-Jun, and its phosphorylation. On the other hand, the addition of GEE partially reversed the suppressing effects of dnNrf2 on cell differentiation, vitamin D- and Nrf2-related gene and/or protein expression, and c-Jun and P-c-Jun levels. Finally, using the transcription factor decoy strategy [22,30], we demonstrated that cell loading with AP-1-specific oligodeoxynucleotide (ODN) markedly inhibited the enhancing effects of CA and MMF on 1,25D_3_-induced expression of myeloid differentiation markers and VDR and RXRα proteins, and transactivation of the vitamin D response element (VDRE).

## 2. Results

### 2.1. Glutathione Depletion Attenuates the Differentiation-Enhancing Effects of Nrf2 Activators

**Figure 1:**
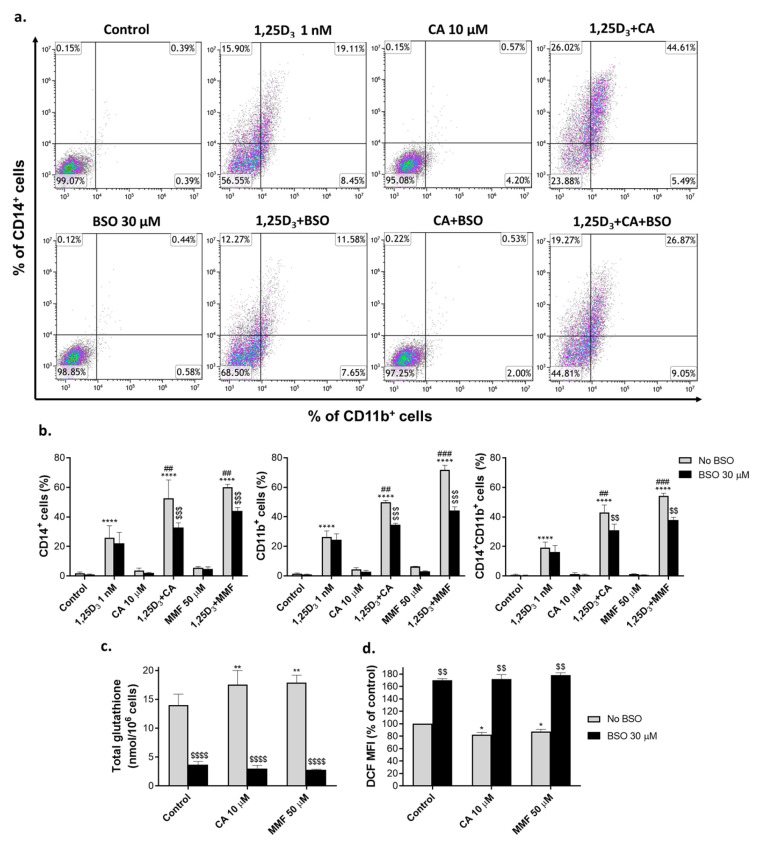
Glutathione depletion by buthionine sulfoximine inhibits the potentiating effects of Nrf2 activators on 1,25D_3_-induced differentiation and elevates ROS levels in HL60 cells. Cells were preincubated with vehicle (water) or 30 μM BSO for 24 h, followed by treatment with the indicated concentrations of 1,25D_3_, carnosic acid (CA), or monomethyl fumarate (MMF), alone or in combination, for another 48 h. (**a**) Representative flow cytometric data showing the enhancing effect of CA on 1,25D_3_-induced surface expression of CD14 and CD11b and the inhibitory effect of BSO on this enhancement. (**b**) Summarized CD14 and CD11b expression data, as exemplified in panel (**a**). (**c**) Changes in the total glutathione content, as determined by the glutathione reductase recycling assay following 24 h of preincubation with BSO followed by 16 h of treatment with CA or MMF. (**d**) Averaged ROS levels measured as DCF geometric mean fluorescence intensity (MFI) units (% of control). Cells were preincubated with BSO for 24 h and treated with or without CA or MMF for an additional 48 h. The data are means ± SD of at least 3 independent experiments performed in duplicate. *, *p* < 0.05; **, *p* < 0.01; ****, *p* < 0.0001 vs. untreated control group; ##, *p* < 0.01; ###, *p* < 0.001 vs. sum of the effects of single agents; $$, *p* < 0.01; $$$, *p* < 0.001; $$$$, *p* < 0.0001 vs. corresponding BSO-untreated group.

To characterize the effect of glutathione depletion on 1,25D_3_-induced differentiation and its enhancement by Nrf2 activators, HL60 cells were preincubated with either vehicle or BSO (30 µM) for 24 h, followed by exposure to 1 nM 1,25D_3_, 10 µM CA or 50 µM MMF alone, or the combinations of 1,25D_3_ with either CA or MMF for another 48 h. The extent of myeloid differentiation was assessed by measuring the surface expression of the specific monocytic marker CD14 and the general myeloid marker CD11b using flow cytometry. In accordance with our previous data (e.g., [17,28]), we found that at the noncytotoxic concentrations used, CA and MMF markedly potentiated the expression of CD14 and CD11b induced by a low concentration of 1,25D_3_ (1 nM) in a synergistic manner (Figure 1a,b and Figure S1). Cotreatment with BSO only slightly influenced 1,25D_3_-induced cell differentiation but significantly attenuated the enhancing effects of the two Nrf2 activators (Figure 1a,b and Figure S1).

To document the inhibition of γGCS activity by BSO in our system, we determined changes in the total cellular glutathione levels using the glutathione reductase recycling assay. As expected, incubation with either CA or MMF significantly elevated the glutathione content (Figure 1c), which was accompanied by lowering the cytosolic ROS levels measured using the DCFH-DA fluorescence probe (Figure 1d and Figure S2). Coincubation with BSO resulted in a marked decrease in both the basal and the treatment-induced glutathione levels, abrogating the CA and MMF stimulation compared to the control cells (Figure 1c). Predictably, this was associated with a marked elevation of ROS levels (Figure 1d and Figure S2). The above results support the notion that glutathione mediates, at least in part, the differentiation-enhancing effects of Nrf2-activating compounds and that this enhancement may involve the ability of glutathione to maintain reducing conditions in AML cells.

### 2.2. Effects of Glutathione Depletion on mRNA and Protein Levels of Molecular Regulators Involved in Cell Differentiation

To explore the role of glutathione in the enhancement of 1,25D_3_-induced differentiation of HL60 cells by Nrf2 activators, we examined the effects of glutathione depletion on the mRNA and protein expression of the transcription factors VDR, RXRα and Nrf2 as well as their target genes. This was carried out using cell samples collected after incubation with 1 nM 1,25D_3_, 10 µM CA, 50 µM MMF, 1,25D_3_/CA, and 1,25D_3_/MMF in the absence or presence of BSO for 48 h, as described in Section 2.1 above.

#### 2.2.1. Glutathione Depletion Differentially Affects mRNA Expression of Vitamin D- and Nrf2-Related Genes

Using quantitative RT-PCR (qPCR), we analyzed mRNA expression of the vitamin D-related genes *VDR*, *RXRA* (RXRα), *CAMP* (cathelicidin antimicrobial peptide), *CYP24A1* (1,25D 24-hydroxylase)*, CD14,* and *ITGAM* (CD11b) as well as *NFE2L2* (Nrf2) and its target genes *NQO1*, *HMOX1* (heme oxygenase 1), *TXNRD1* (thioredoxin reductase 1), *GCLC* (catalytic subunit of γGCS), and *GCLM* (modifier subunit of γGCS).

**Figure 2:**
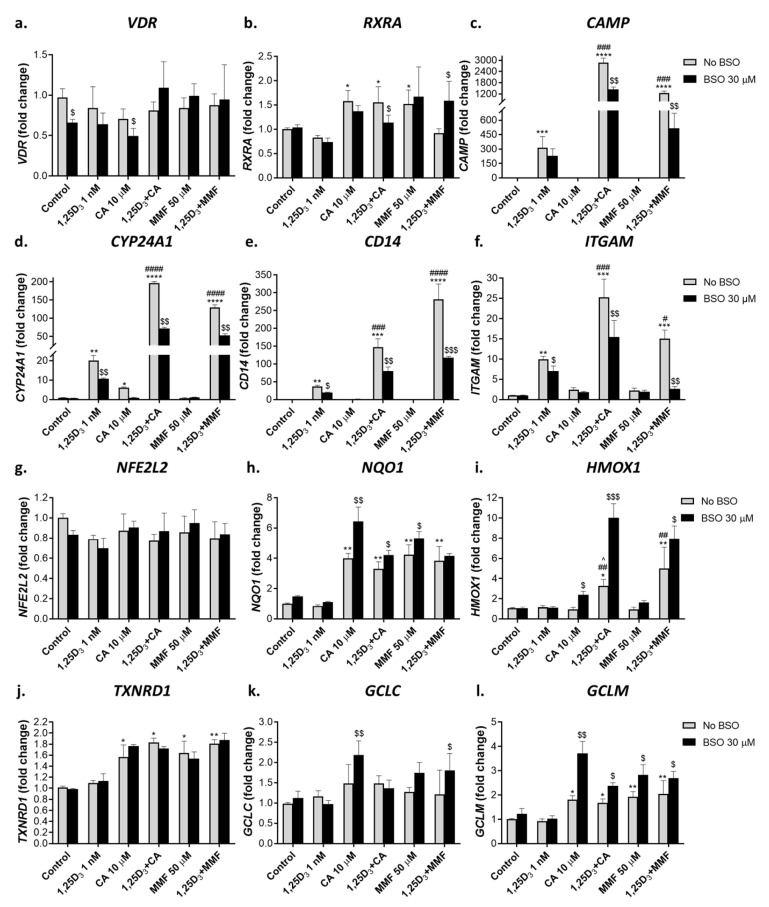
Effects of glutathione depletion on mRNA levels of vitamin D- and Nrf2-related genes. HL60 cells were incubated with the indicated agents for 48 h, as described in the legend to Figure 1. Cell samples were then analyzed for mRNA levels of the indicated genes using quantitative RT-PCR. The expression of specific genes was normalized by the C_T_ value of the internal reference gene (*GAPDH*). The data are means ± SD of 3 experiments performed in triplicate. *, *p* < 0.05; **, *p* < 0.01; ***, *p* < 0.001; ****, *p* < 0.0001 vs. untreated control group; #, *p* < 0.05; ##, *p* < 0.01; ###, *p* < 0.001; ####, *p* < 0.0001 vs. sum of the effects of single agents; $, *p* < 0.05; $$, *p* < 0.01; $$$, *p* < 0.001 vs. corresponding BSO-untreated group.

As shown in Figure 2a, single or combined treatments with 1,25D_3_, CA, or MMF either did not affect or even slightly reduced *VDR* mRNA levels and moderately elevated *RXRA* expression (Figure 2b). On the other hand, a marked induction of all the VDR target genes tested was detected in 1,25D_3_-treated cells, while neither CA nor MMF alone had noticeable effects. However, combining 1,25D_3_ with either Nrf2 activator resulted in a substantial synergistic upregulation of these genes (Figure 2c–f). Of note, the upregulated *CD14* and *ITGAM* expression strongly correlated with the elevated cell surface levels of CD14 and CD11b, respectively (see Figure 1a,b). Similar to *VDR*, the expression of *NFE2L2* was practically unaltered by our treatments (Figure 2g). Yet, CA or MMF significantly upregulated the Nrf2 target genes *NQO1*, *TXNRD1*, and *GCLM* (Figure 2h,j,l). It was noted that 1,25D_3_ did not potentiate these effects, nor did it significantly induce any of the Nrf2-responsive genes tested when applied alone. However, both CA and MMF synergistically cooperated with 1,25D_3_ in upregulating *HMOX1* (Figure 2i). Unlike *GCLM*, the expression of *GCLC* was unresponsive to any treatment (Figure 2k).

Glutathione depletion with BSO slightly affected *VDR* and *RXRA* expression (Figure 2a,b,g). Nonetheless, it markedly suppressed the induction of the vitamin D-responsive genes in cells exposed to 1,25D_3_, alone or together with CA or MMF (Figure 2c–f). This suppression correlated with the suppression of cell differentiation in BSO-treated cells (see Figure 1a,b). In contrast, BSO treatment further enhanced the induction of all the Nrf2 target genes, except *TXNRD1,* without affecting *NFE2L2* expression (Figure 2g–l).

#### 2.2.2. Glutathione Depletion Differentially Affects the Expression of Proteins Encoded by Vitamin D- and Nrf2-Related Genes

**Figure 3:**
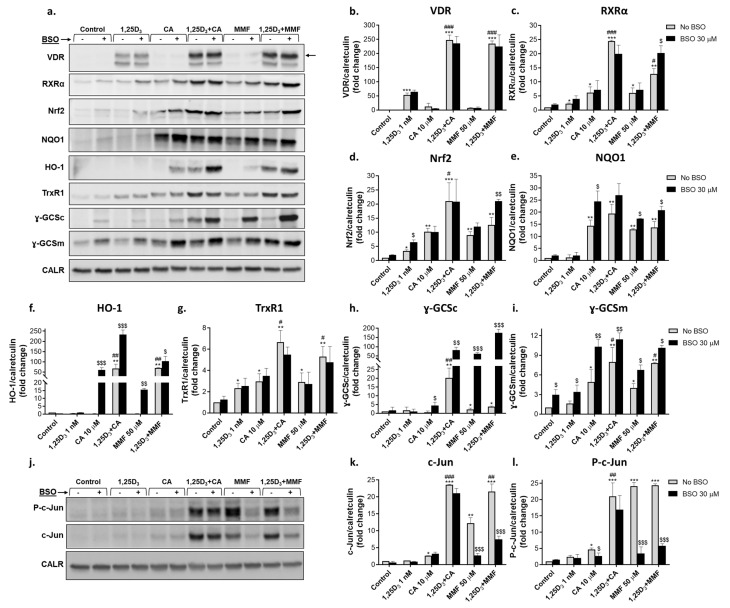
Effects of glutathione depletion on the levels of vitamin D- and Nrf2-related proteins, c-Jun and its phosphorylation. HL60 cells were incubated with the indicated agents, as described in the legend to Figure 1. Cell samples were analyzed by Western blotting. Calreticulin was used as the protein loading control. (**a**,**j**) Representative Western blot images. (**b**–**i**,**k**,**l**) Absorbance values for specific proteins were normalized to those of calreticulin and expressed in the bar graphs as fold change relative to the corresponding untreated controls. The data are means ± SD of 3 experiments. *, *p* < 0.05; **, *p* < 0.01; ***, *p* < 0.001 vs. untreated control cells; #, *p* < 0.05; ##, *p* < 0.01; ###, *p* < 0.001 vs. sum of the effects of single agents; $, *p* < 0.05; $$, *p* < 0.01; $$$, *p* < 0.001 vs. corresponding BSO-untreated group.

Western blot analysis was used to determine protein levels of VDR and RXRα as well as Nrf2 and the proteins encoded by its target genes: NQO1, heme oxygenase 1 (HO-1), thioredoxin reductase 1 (TrxR1), and the catalytic (ɣ-GCSc) and modifier (ɣ-GCSm) subunits of γGCS. As shown in Figure 3a,b, treatment with 1,25D_3_ strongly increased VDR levels, while CA or MMF had only slight effects. However, both Nrf2 activators dramatically enhanced the impact of 1,25D_3_ in a synergistic manner. Unlike VDR, RXRα expression was more responsive to CA or MMF than to 1,25D_3_ and combined treatments produced a synergistic effect (Figure 3a,c). As expected, protein levels of Nrf2 and most of its target gene products (NQO1, TrxR1, ɣ-GCSc, and ɣ-GCSm) were significantly upregulated by CA and/or MMF (Figure 3a,d,e,g–i). While 1,25D_3_ alone had moderate or no effect, it was capable of potentiating the impact of one (Figure 3a,d,h) or both (Figure 3a,f,g,i) Nrf2 activators in most cases. Interestingly, HO-1 expression was insensitive to single agents, but it was highly evident in combination-treated cells (Figure 3a,f). Likewise, ɣ-GCSc levels were elevated by the 1,25D_3_/CA combination, while neither 1,25D_3_ nor CA alone had any significant effect (Figure 3a,h).

As BSO markedly inhibited VDR target gene induction without consistently affecting *VDR* and *RXRA* mRNA levels (see Figure 2a–f), it also had no effect on VDR and RXRα protein levels in cells treated with 1,25D_3_ and Nrf2 activators separately or the 1,25D_3_/CA combination. The effect of 1,25D_3_ + MMF on RXRα expression was even potentiated under these conditions (Figure 3a–c). The enhanced induction of most Nrf2-regulated genes in glutathione-depleted cells (see Figure 2h,i,k,l) was generally paralleled by increased upregulation of the corresponding proteins (Figure 3e,f,h,i). The Nrf2 protein levels also tended to increase following some of the treatments (Figure 3d), even though the *NFE2L2* gene expression was unaffected by BSO (Figure 2g).

We have previously shown that the transcription factor AP-1 is activated via concerted actions of 1,25D_3_ and plant polyphenols in HL60 and U937 AML cells. This was characterized by increased AP-1 DNA binding and transcriptional activity and associated with upregulation and phosphorylation of the AP-1 family proteins c-Jun and ATF-2 [22,24,29]. Consistent with these findings, both 1,25D_3_/CA and 1,25D_3_/MMF combinations synergistically upregulated c-Jun at comparable magnitudes. Interestingly, when added alone, MMF was a more potent inducer than 1,25D_3_ or CA (Figure 3j,k). The levels of the phosphorylated (activated) form of c-Jun (P-c-Jun) were similarly elevated by the above treatments, MMF alone being as effective as its combination with 1,25D_3_ (Figure 3j,l). Notably, the increases in both c-Jun and P-c-Jun levels caused by MMF, with or without 1,25D_3_, were dramatically reduced following coincubation with BSO, whereas the effects of CA ± 1,25D_3_ were slightly affected (Figure 3j,k,l).

In summary, the above data indicate that glutathione depletion in HL60 cells resulted in a marked inhibition of VDR target gene expression induced by 1,25D_3_, particularly in combination with Nrf2 activators. This occurred without significantly reducing VDR mRNA and protein levels, while RXRα expression even tended to increase. On the other hand, the induction of Nrf2 and its target genes by Nrf2 activators and their combinations with 1,25D_3_ was generally augmented in glutathione-depleted cells. Our results also suggested that cellular glutathione is necessary for upregulating c-Jun protein levels and phosphorylation induced by MMF, alone and combined with 1,25D_3_, and is less critical for c-Jun regulation by 1,25D_3_ ± CA.

### 2.3. Introduction of Exogenous GSH Partially Reverses the Suppressing Effect of a Dominant-Negative Nrf2 Mutant on Myeloid Differentiation of HL60 Cells

**Figure 4:**
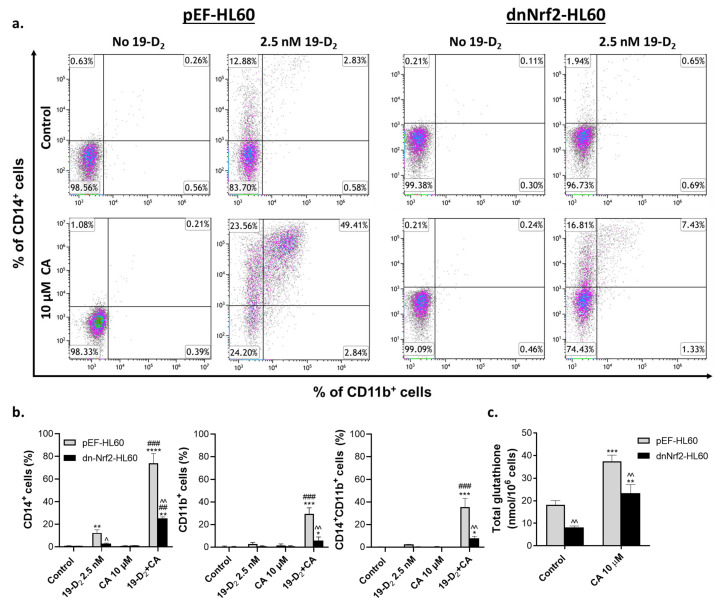
Stable expression of dominant-negative Nrf2 suppresses the differentiation induced by paricalcitol and its combination with carnosic acid and reduces glutathione levels. Cells stably transfected with either empty vector (pEF-HL60) or dominant-negative Nrf2 (dnNrf2-HL60) were incubated with the indicated concentrations of paricalcitol (19-D_2_) and carnosic acid (CA), alone or combination, for 48 h. (**a**) Representative flow cytometric data showing changes in CD14 and CD11b surface expression. (**b**) Summarized CD14 and CD11b expression data exemplified in panel (**a**). (**c**) pEF-HL60 and dnNrf2-HL60 cells differ in the total glutathione content. Cells were treated with vehicle or the indicated concentrations of CA for 48 h. The total cellular glutathione (GSH + GSSG) concentration was determined via the glutathione reductase recycling assay. The data are means ± SD of at least 3 independent experiments performed in duplicate. *, *p* < 0.05; **, *p* < 0.01; ***, *p* < 0.001; ****, *p* < 0.0001 vs. corresponding control group; ##, *p* < 0.01; ###, *p* < 0.001 vs. corresponding sum of the effects of single agents; ^, *p* < 0.05; ^^, *p* < 0.01, dnNrf2-HL60 cells vs. pEF-HL60 cells.

To further explore the role of glutathione in VDD-induced differentiation and its enhancement via Nrf2 activation, we employed HL60 cells stably expressing a dominant-negative Nrf2 mutant (dnNrf2), which lacks the transactivation domain [22,31], and those transfected with empty vector (pEF). These experiments were performed using the clinically approved vitamin D_2_ analog paricalcitol [32] as a VDD and CA as an Nrf2 activator. We have reported that 1,25D_3_ and paricalcitol display comparable differentiation-inducing potencies in the absence or presence of Nrf2 activators [28]. Similar to our data obtained in dnNrf2-expressing U937 cells [22], the extent of myeloid differentiation induced by paricalcitol and enhanced by CA was substantially lower in dnNrf2-HL60 cells than in pEF-HL60 cells (Figure 4a,b).

Since dnNrf2 inhibits Nrf2 transcriptional activity, we hypothesized that dnNrf2-HL60 cells produce less glutathione than pEF-HL60 controls. Indeed, the basal and CA-induced total glutathione production in dnNrf2-HL60 was significantly lower than in pEF-HL60 cells (Figure 4c). Thus, we suggested that reintroducing GSH to dnNrf2-HL60 cells would improve the differentiation response to paricalcitol and its combination with CA. Since GSH is a well-known antioxidant, we also examined if the potential improvement of differentiation would specifically be attributed to the molecular features of GSH or would be due to its general antioxidant effect. To this end, we compared the effects of a membrane-permeable GSH ethyl ester (GEE) and an unrelated antioxidant, Trolox, on pEF-HL60 and dnNrf2-HL60 cell differentiation. Cells were preincubated with a vehicle, 250 µM GEE or 300 µM Trolox for 1 h, followed by treatment with 2.5 nM paricalcitol, 10 µM CA or their combination, for another 48 h, followed by the flow cytometric CD14 and CD11b assay.

**Figure 5:**
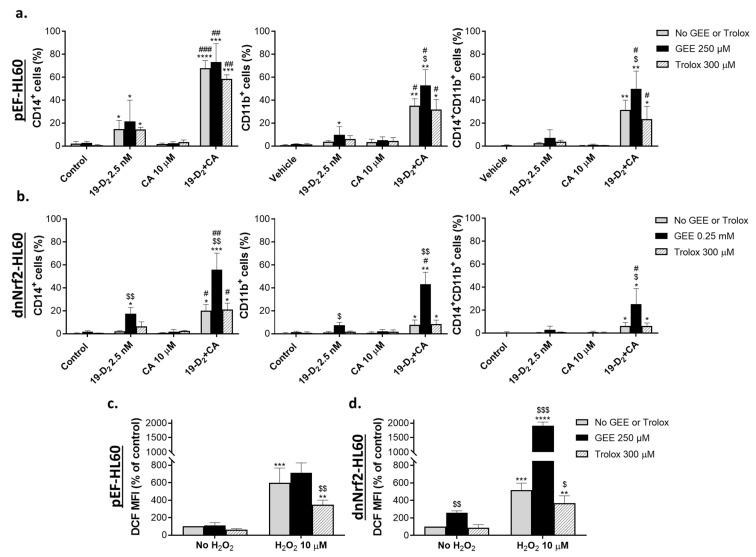
GSH ethyl ester, but not Trolox, reverses the inhibitory effect of dominant-negative Nrf2 on the differentiation of HL60 cells while increasing cytosolic ROS levels. (**a**,**b**) Effects of GEE or Trolox on cell differentiation. Cells were preincubated with vehicle, GEE or Trolox for 1 h, followed by the addition of paricalcitol, CA or their combination and then incubated for another 48 h. The expression of CD14 and CD11b was determined via flow cytometry. *, *p* < 0.05; **, *p* < 0.01; ***, *p* < 0.001; ****, *p* < 0.0001, vs. corresponding control group; #, *p* < 0.05; ##, *p* < 0.01; ###, *p* < 0.001, vs. corresponding sum of the effects of single agents; $, *p* < 0.05; $$, *p* < 0.01, GEE-treated vs. corresponding GEE-untreated group. (**c**,**d**) Changes in cytosolic ROS levels. Cells were incubated with vehicle, GEE or Trolox for 48 h, followed by exposure to 10 µM H_2_O_2_ for an additional 15 min. The results are expressed as DCF geometric mean fluorescence intensity (MFI) units (% of control). The data are means ± SD of 3 independent experiments. **, *p* < 0.01; ***, *p* < 0.001; ****, *p* < 0.0001, vs. corresponding H_2_O_2_-untreated group; $, *p* < 0.05; $$, *p* < 0.01; $$$, *p* < 0.001 vs. corresponding GEE- or Trolox-untreated group.

The results demonstrated that Trolox did not affect either the basal or induced cell differentiation of dnNrf2-HL60 and pEF-HL60 cells (Figure 5a,b). In contrast, the addition of GEE essentially reversed the inhibitory effect of dnNrf2 on cell differentiation, restoring the responsiveness of dnNrf2-HL60 cells to paricalcitol ± CA nearly up to the levels detected in pEF-HL60 cells. In pEF-HL60 cells, the augmenting effect of GEE was relatively less pronounced compared to dnNrf2-HL60 cells (Figure 5a,b). N-acetyl cysteine (1000 µM), a precursor of L-cysteine, which is the rate-limiting factor in cellular glutathione biosynthesis [33], also tended to enhance the differentiation of dnNrf2-HL60 cells; however, it was less effective than GEE (Supplementary Figure S3b). There was no enhancement in NAC-treated pEF-HL60 cells and even a small reduction in CD14 and CD11b surface expression was observed (Supplementary Figure S3a).

We then examined whether the difference between GEE and Trolox in affecting cell differentiation correlated with their possible differential influence on intracellular ROS levels. Thus, the abilities of the two compounds to counteract H_2_O_2_-induced ROS generation in pEF-HL60 and dnNrf2-HL60 cells were determined. Cells were incubated with vehicle, 250 µM GEE, or 300 µM Trolox for 48 h, followed by exposure to 10 µM H_2_O_2_ for an additional 15 min. Cytosolic ROS levels were then measured via flow cytometry using the fluorescent probe DCFH_2_-DA. The data demonstrated that in pEF-HL60 cells, the basal ROS level was unaffected by either GEE or Trolox (Figure 5c), while in dnNrf2-HL60 cells, it was surprisingly elevated by GEE, but not by Trolox (Figure 5d). Incubation of vehicle-treated cells of both types with 10 µM H_2_O_2_ resulted in similar increases in ROS levels (Figure 5c,d). The addition of H_2_O_2_ to GEE-treated pEF-HL60 cells had approximately the same effect as in the vehicle-treated cells (Figure 5c); however, in GEE-treated dnNrf2-HL60 cells, the pro-oxidant effect of H_2_O_2_ was markedly enhanced (Figure 5d). In contrast, Trolox treatment significantly prevented H_2_O_2_-induced ROS generation in both cell types (Figure 5c,d).

Collectively, the results demonstrated that the inhibitory effect of dnNrf2 on paricalcitol ± CA-induced differentiation of HL60 cells correlated with decreased glutathione production (Figure 4 and Figure 5), whereas adding GEE reversed this inhibition. Notably, the rescuing effect of GEE was opposite to that of the glutathione-depleting agent BSO, which suppressed differentiation of intact HL60 cells (Figure 1a,b), even though both compounds appeared to act as pro-oxidants (compare Figure 1d and Figure 5d). Combined with the fact that the antioxidant Trolox did not affect the surface expression of CD14 and CD11b, the above opposite effects of GEE and BSO implied that in our system, the differentiation-promoting action of glutathione was not mediated via cytosolic ROS.

### 2.4. Effects of Exogenous GSH on mRNA and Protein Levels of Molecular Regulators of Cell Differentiation in Vector-Transfected and Dominant-Negative Nrf2-Expressing HL60 Cells

We next determined changes in gene and protein expression associated with restoring paricalcitol ± CA-induced differentiation of dnNrf2-expressing HL60 cells by GEE. For this purpose, pEF-HL60 and dnNrf2-HL60 cells were exposed to vehicle, 2.5 nM paricalcitol, 10 µM CA, with or without 250 µM GEE, for 48 h followed by qPCR and Western blot analyses, as described above (Section 2.2).

#### 2.4.1. Regulation of Vitamin D- and Nrf2-Related Gene Expression by GSH Ethyl Ester

**Figure 6:**
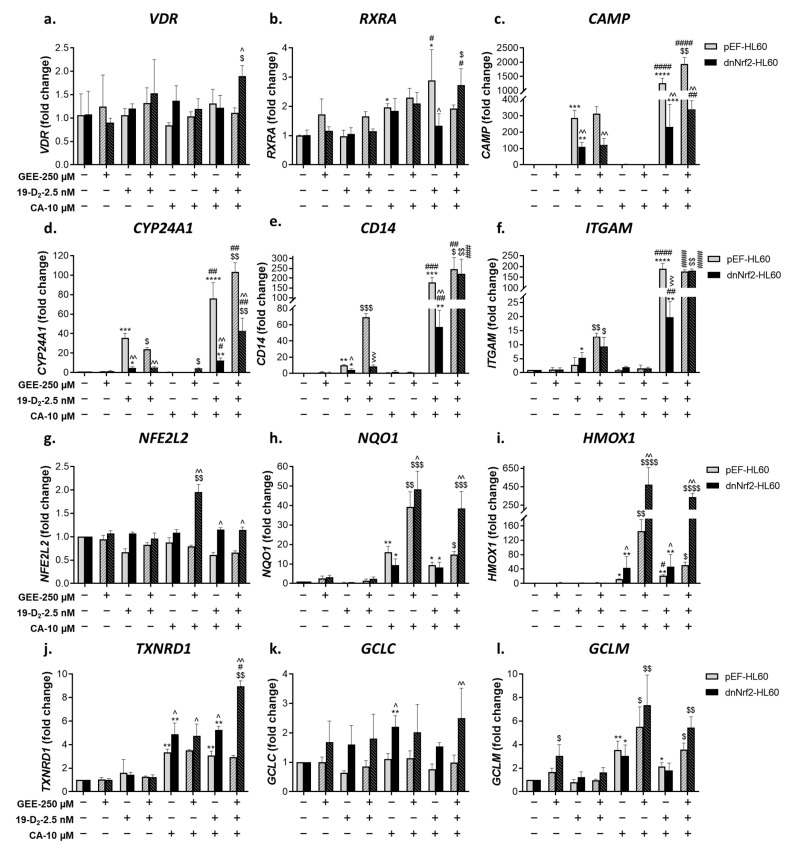
Effects of GSH ethyl ester on mRNA levels of vitamin D- and Nrf2-related genes. pEF-HL60 and dnNrf2-HL60 cells were incubated with the indicated agents for 48 h. Samples were analyzed for mRNA levels of the indicated genes using quantitative RT-PCR. The expression of specific genes was normalized by the C_T_ value of the internal reference gene (*GAPDH*). The data are means ± SD of 3 experiments performed in triplicate. *, *p* < 0.05; **, *p* < 0.01; ***, *p* < 0.001; ****, *p* < 0.0001 vs. corresponding untreated control group; #, *p* < 0.05; ##, *p* < 0.01; ###, *p* < 0.001; ####, *p* < 0.0001 vs. corresponding sum of the effects of single agents. $, *p* < 0.05; $$, *p* < 0.01; $$$, *p* < 0.001; $$$$, *p* < 0.0001, GEE-treated vs. corresponding GEE-untreated group. ^, *p* < 0.05; ^^, *p* < 0.01; ^^^, *p* < 0.001, dnNrf2-HL60 cells vs. pEF-HL60 cells.

As shown in Figure 6, basal mRNA levels of the vitamin D- and Nrf2-related genes tested were similar in pEF-HL60 and dnNrf2-HL60 cells. Consistent with the data obtained in nontransfected HL60 cells (Figure 2), in the vector-transfected cells, the expression of *VDR* was unaffected by paricalcitol ± CA treatments (Figure 6a), and *RXRA* was moderately induced by CA and its combination with paricalcitol (Figure 6b). The expression of VDR target genes (*CAMP*, *CYP24A1*, *CD14*, and *ITGAM*) was upregulated to a varying extent by the VDD and further substantially enhanced by adding the polyphenol (Figure 6c–f). Notably, stable transfection of dnNrf2 in HL60 cells generally reduced the responsiveness of the above target genes to paricalcitol and markedly suppressed the enhancing effects of CA relative to those detected in pEF-HL60 cells (Figure 6c–f). This inhibition correlated with relatively lower CD14 and CD11b surface levels in paricalcitol ± CA-treated dnNrf2-HL60 cells, as determined via flow cytometry (Figure 4 and Figure 5).

The introduction of exogenous GSH in the form of GEE was generally without a significant effect on *VDR* and *RXRA* mRNA levels, except moderately upregulating these genes in paricalcitol/CA-treated dnNrf2-HL60 cells, but not in pEF-HL60 cells (Figure 6b). In contrast to the inhibitory effect of glutathione depletion on the expression of VDR target genes (Figure 2c–f), coincubation with GEE mostly had a positive impact, depending on the treatment and transfectant type. For instance, GEE did not significantly affect *CAMP* and *CYP24A1* induction by paricalcitol alone in either pEF-HL60 or dnNrf2-HL60 cells while tending to augment the effect of the paricalcitol/CA combination to some degree (Figure 6c,d). On the other hand, paricalcitol alone-induced expression of *CD14* and *ITGAM* was enhanced in the presence of GEE in both cell types. In pEF-HL60 cells, GEE did not further augment paricalcitol/CA-induced *CD14* and *ITGAM* upregulation. However, GEE addition to dnNrf2-HL60 cells completely restored the synergistic induction of the two genes to the levels established in the pEF-HL60 controls (Figure 6e,f).

Similar to nontransfected HL60 cells, CA or paricalcitol did not induce *NFE2L2* in either cell type (Figure 6g). As expected, CA significantly upregulated most of the tested genes known to be driven by Nrf2 (*NQO1*, *HMOX1*, *TXNRD1,* and *GCLM*) in pEF-HL60 cells. Paricalcitol alone had no effect and even tended to attenuate the CA induction of *NQO1* and *GCLM*, but it could positively cooperate with CA in inducing *HMOX1* (Figure 6h–l). Surprisingly, none of these genes were significantly repressed by dnNrf2 transfection. Instead, there was even greater upregulation of *NFE2L2*, *HMOX1*, *TXNRD1*, and *GCLC* in dnNrf2-HL60 cells relative to pEF-HL60 cells (Figure 6g–l).

Interestingly, similar to BSO-treated HL60 cells (Figure 2h,i,k,l), coincubation with GEE led to a significant potentiation of CA- and/or paricalcitol/CA-induced expression of Nrf2-related genes either in both transfectant types (*NQO1*, *HMOX1,* and *GCLM)* or just in dn-Nrf2-HL60 cells (*NFE2L2*, *TXNRD1*, and *GCLC)*, the latter transfectant displaying more robust responses (Figure 6g–l).

#### 2.4.2. Regulation of Vitamin D- and Nrf2-Related Protein Expression by GSH Ethyl Ester

**Figure 7:**
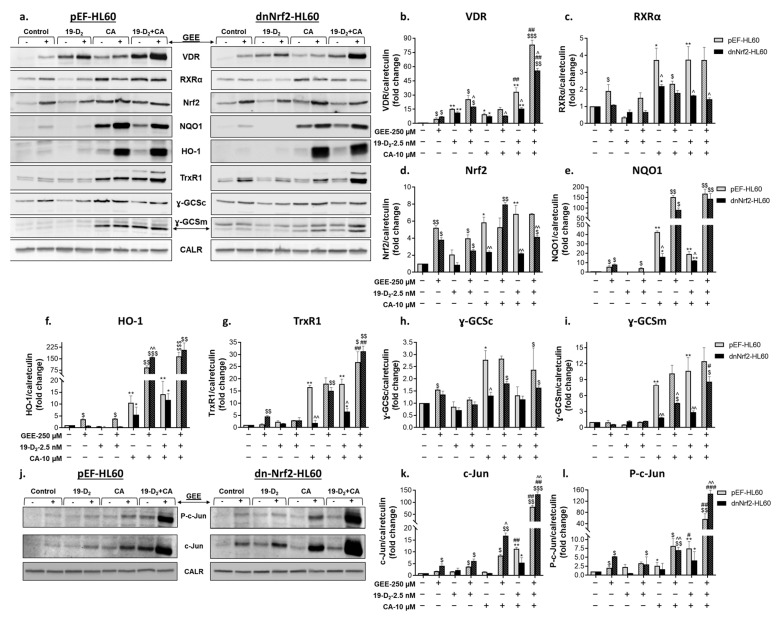
Effects of GSH ethyl ester on the levels of vitamin D- and Nrf2-related proteins, c-Jun and its phosphorylation. pEF-HL60 and dnNrf2-HL60 cells were incubated with the indicated agents for 48 h. Samples were analyzed for the expression of the indicated proteins via Western blotting. Calreticulin was used as a protein loading control. (**a**,**j**) Representative Western blot images. (**b–i,k,l**) Absorbance values for specific proteins normalized to those of calreticulin and expressed in the bar graphs as fold change relative to the corresponding untreated controls. The data are means ± SD of 3 experiments. *, *p* < 0.05; **, *p* < 0.01 vs. corresponding control group; #, *p* < 0.05; ##, *p* < 0.01; ###, *p* < 0.001 vs. corresponding sum of the effects of single agents; $, *p* < 0.05; $$, *p* < 0.01; $$$, *p* < 0.001, GEE-treated vs. corresponding GEE-untreated group; ^, *p* < 0.05; ^^, *p* < 0.01, dnNrf2-HL60 cells vs. pEF-HL60 cells.

Western blot analysis demonstrated that in pEF-HL60 cells, both paricalcitol and, to a lesser extent, CA upregulated VDR protein levels, and their combination produced a synergistic effect (Figure 7a,b). RXRα expression was induced by CA while paricalcitol was ineffective either in the absence or presence of the polyphenol (Figure 7a,c). Stable transfection of dnNrf2 slightly affected VDR upregulation by single compounds but practically abolished their synergistic activity. Likewise, RXRα induction by CA ± paricalcitol was significantly weaker in dnNrf2-HL60 cells compared to their empty vector-transfected counterparts (Figure 7a–c). CA significantly upregulated Nrf2 and its target gene products NQO1, HO-1, TrxR1, γ-GCSc, and γ-GCSm in pEF-HL60 cells, and this effect was predictably less pronounced in dnNrf2-HL60 cells. Paricalcitol did not significantly affect the levels of these proteins or their upregulation by CA in any of the two cell types (Figure 7a,d–i).

Interestingly, cotreatment with GEE increased the basal levels of most vitamin D- and Nrf2-related proteins tested in pEF-HL60 and dnNrf2-HL60 cells (Figure 7a–i). GEE further augmented VDR upregulation by paricalcitol ± CA in both cell types and largely restored its synergistic induction by the VDD/CA combination in dnNrf2-HL60 cells (Figure 7a,b). Likewise, the upregulation of Nrf2 and the related proteins by CA and/or its combination with paricalcitol was generally enhanced by GEE, to a varying extent, in one or both cell types (Figure 7a,d–i). On the other hand, CA-induced RXRα upregulation was surprisingly suppressed by GEE in these samples, while the effects of the paricalcitol/CA combination did not change significantly (Figure 7a,c).

The protein levels of c-Jun and P-c-Jun increased to some extent following single treatments with paricalcitol and CA in both pEF-HL60 and dnNrf2-HL60 cells, but a cooperative effect of the combination was clearly seen only in pEF-HL60 cells (Figure 7j,k,l). Adding GEE moderately increased the basal and paricalcitol-induced expression of c-Jun and its phosphorylated form. However, it dramatically potentiated the effects of CA and its combination with paricalcitol, restoring the synergy between the two agents in dnNrf2-HL60 cells (Figure 7j,k,l).

In summary, cotreatment with GEE enhanced the induction of VDR (Figure 7a,b) and its target genes by paricalcitol and its combination with CA (Figure 6c–f). A similar enhanced upregulation of c-Jun and its phosphorylated form was also observed (Figure 7j–l). These findings directly correlated with augmenting paricalcitol ± CA-induced myeloid differentiation, especially in dnNrf2-expressing cells (Figure 5b,c). The induction of Nrf2 (Figure 6g and Figure 7d) and its target genes (Figure 6h–l and Figure 7e–i) by CA ± paricalcitol increased in the presence of exogenous GSH. However, similar increases were observed in glutathione-depleted cells (see Figure 2h,i,k,l and Figure 3d–f,h,i), in which the induction of differentiation was suppressed (Figure 1a,b). The complex relationship between glutathione and the Nrf2/ARE pathway in AML cells induced to differentiate by VDDs and their combination with Nrf2 activators is addressed in Section 3.

### 2.5. Involvement of AP-1 in the Regulation of VDR/RXRα Protein Expression and Transcriptional Activity and the Enhancement of 1,25D_3_-Induced Cell Differentiation by Nrf2 Activators

As demonstrated above, glutathione depletion resulted in a downregulation of c-Jun protein expression, whereas the introduction of external GSH produced the opposite effect. These data suggest that glutathione may regulate AP-1 expression and activity, which in turn may influence VDD-induced myeloid differentiation of AML cells. We, thus, employed the AP-1 decoy strategy to explore the involvement of AP-1 in the differentiation of HL60 cells, VDR and RXRα protein expression, and VDRE transactivation induced by 1,25D_3_ and its combinations with Nrf2 activators.

**Figure 8:**
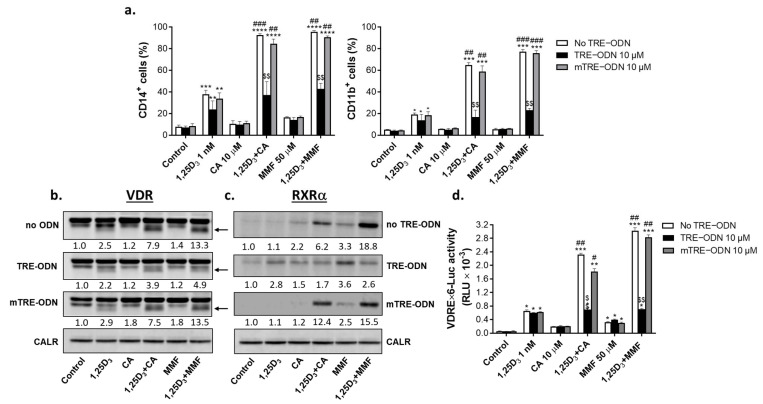
Involvement of AP-1 in the enhancing effects of Nrf2 activators on 1,25D_3_-induced cell differentiation, VDR and RXRα protein expression, and VDRE transactivation. HL60 cells were preincubated for 24 h either without oligodeoxynucleotide (ODN) or with 10 μM TRE-ODN or mTRE-ODN followed by treatment with 1,25D_3_, CA, MMF, or their combinations at the indicated concentrations for an additional 48 h. (**a**) Averaged CD14 and CD11b surface expression, as measured via flow cytometry. (**b**,**c**) Representative Western blots showing changes in VDR and RXRα protein levels. Calreticulin was used as a protein loading control. (**d**) Cells were transiently transfected with VDREx6-Luc and Renilla luciferase plasmids, followed by pretreatment with or without TRE-ODN or mTRE-ODN for 24 h. Cells were then incubated with or without the indicated test agents for another 24 h, followed by measuring luciferase activity. The data are means ± SD of 3 experiments. *, *p* < 0.05; **, *p* < 0.01; ***, *p* < 0.001; ****, *p* < 0.0001 vs. corresponding control group; #, *p* < 0.05; ##, *p* < 0.01; ###, *p* < 0.001; combination vs. corresponding sum of the effects of single agents; $$, *p* < 0.01, TRE-ODN-treated group vs. corresponding TRE-ODN untreated group.

Cells were preincubated with either vehicle, 10 µM TRE-ODN, or mutant TRE (mTRE)-ODN, for 24 h followed by treatment with 1 nM 1,25D_3_, 10 µM CA, or 50 µM MMF, alone or in combination, for an additional 48 h. The results demonstrated that neither TRE-ODN nor mTRE-ODN affected CD14 and CD11b surface expression induced by 1,25D_3_ alone; however, the differentiation-enhancing effects of CA and MMF were practically abolished by TRE-ODN, but not by mTRE-ODN (Figure 8a). Notably, this was paralleled by a marked reduction in VDR and RXRα protein levels, as determined via Western blotting (Figure 8b,c). Consistently, the VDR/RXRα transcriptional activity was also profoundly reduced by TRE-ODN, as measured via the VDRE-luciferase reporter assay (Figure 8d).

These results strongly suggested that AP-1 is essential for the synergistic enhancement of 1,25D_3_-induced differentiation of AML cells by CA and MMF, likely through the positive regulation of VDR/RXRα protein levels and transcriptional activity.

## 3. Discussion

Accumulating evidence indicates that the transcription factors Nrf2 [34,35,36,37,38] and AP-1 [30,39,40,41,42,43,44] play significant roles in the differentiation of various normal and cancer cell types, including hematopoietic cells. We have previously reported that the antileukemic synergy between VDDs and Nrf2 activators is associated with a mutual upregulation of VDR and Nrf2 signaling [22,28] and that Nrf2 may function as an upstream regulator of VDR, RXRα, and AP-1 protein levels in AML cells [22]. However, the mode of the interaction between the Nrf2, AP-1 and VDR/RXRα pathways remains unclear. The present study was designed to explore the role of glutathione as the potential mediator of the differentiation-enhancing effects of Nrf2 activators in this system.

Glutathione is a ubiquitous thiol tripeptide synthesized in the cytosol by consecutive action of two enzymes, γ-GCS and glutathione synthetase, and reaches millimolar intracellular concentrations. γ-GCS catalyzes the synthesis of γ-glutamylcysteine from L-glutamate and L-cysteine, and the glutathione synthetase-catalyzed addition of L-glycine completes the formation of glutathione [26,33]. Nrf2, AP-1, and nuclear factor kappa B (NFκB) are among the key transcription factors that regulate the expression of these enzymes [33]. The reduced form of glutathione (GSH) functions as the principal cellular reducing agent and antioxidant and participates in various regulatory processes, including cytoprotection, cell signaling, metabolism of xenobiotics, gene expression, protein synthesis and modification, cell cycle, apoptosis, and immunomodulation [26,45,46,47,48].

Glutathione depletion has been shown to impair the differentiation of various cell types. For instance, Esposito et al. [49] reported that the glutathione-conjugating compound diethyl maleate inhibited 12-O-tetradecanoylphorbol-13-acetate (TPA)-driven differentiation of HL60 and KG-1 AML cells. We showed that the specific γGCS inhibitor BSO attenuates 1,25D_3_- and 1,25D_3_/CA-induced differentiation of HL60 cells [24]. Similar results were obtained in other cell types, such as T cells [50], dendritic cells [51], macrophages [52], C2C12 skeletal muscle cells [53], osteoblasts [54], and osteoclasts [55]. These data indicate an essential role of glutathione in cell maturation.

**Figure 9:**
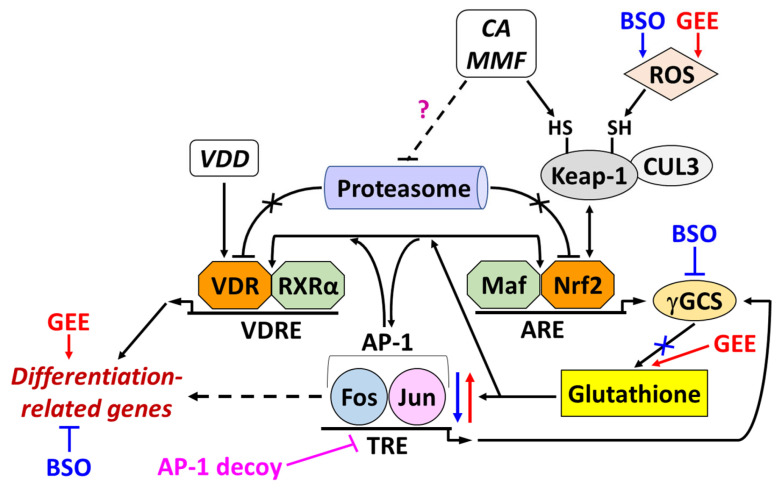
Putative roles of glutathione and AP-1 in the cooperation between VDDs and Nrf2 activators in inducing differentiation of AML cells. Natural (pro)electrophilic compounds, such as carnosic acid (CA) and monomethyl fumarate (MMF), can synergistically enhance the differentiation-inducing effects of vitamin D derivatives (VDDs). Electrophilic modification of the cysteine-rich protein Keap-1 leads to the activation of the Nrf2/Antioxidant Response Element (Nrf2/ARE) signaling pathway. Our previous studies have shown that Nrf2/ARE activation results in the upregulation of the functional vitamin D receptor (VDR/RXRα) in AML cells, which may account for cell sensitization to low concentrations of VDDs in the presence of electrophilic agents. Conversely, VDDs can potentiate electrophile-induced Nrf2/ARE activation. Still, the mechanisms underlying the bidirectional interplay between Nrf2/ARE and VDR/RXRα remain obscure. Here, using glutathione depletion and repletion approaches, we obtained evidence that this reducing agent, whose synthesis is controlled by Nrf2, AP-1 and other transcription factors, is important for the synergistic activation of both VDR/RXRα and Nrf2/ARE by VDDs and Nrf2 activators. Thus, glutathione can, at least partly, mediate the interplay between these transcription pathways. Our data also suggested that the positive effect of glutathione on VDR/RXRα levels and activity and differentiation induction may, in turn, be mediated by AP-1, e.g., through upregulating and activating c-Jun. Interestingly, CA and MMF promoted the elevation of VDR and Nrf2 protein levels without affecting their mRNA expression, suggesting that these compounds can increase VDR and Nrf2 protein stability, likely by inhibiting their proteasomal degradation. On the other hand, Nrf2 activators can induce *RXRA* (RXRα) gene expression, thereby directly contributing to the synergistic activation of the VDR/RXRα pathway. Exogenous GSH could further enhance the synergistic effects of VDDs and Nrf2 activators on the levels of VDR, some Nrf2-related proteins, and c-Jun. The mechanism underlying this enhancement remains to be elucidated. In summary, the interplay between Nrf2/ARE, AP-1 and VDR/RXRα appears to be a complex process involving multiple molecular mechanisms. See more detailed explanations in Section 3.

The main findings of the present study are summarized below and schematically represented in Figure 9.

### 3.1. Involvement of Glutathione in the Regulation of VDR Signaling and Myeloid Differentiation

We found that glutathione depletion and repletion had opposite impacts on the induction of monocytic differentiation of HL60 cells. Although BSO minimally attenuated the 1,25D_3_-induced surface expression of CD14 and CD11b, it significantly suppressed the enhancing effects of both Nrf2 activators. This correlated with decreased induction of *CD14* and *ITGAM* and two other known vitamin D-responsive genes, *CAMP* and *CYP24A1*. Notably, target gene expression was inhibited without noticeably reducing mRNA and protein levels of VDR and RXRα, suggesting that glutathione depletion primarily inhibited VDR transcriptional activity in our system.

In contrast to BSO, exogenous GSH (as GEE) or, to a lesser extent, its precursor NAC augmented cell differentiation induced by paricalcitol and its combination with CA. This effect was especially pronounced in dnNrf2-expressing HL60 cells, which exhibited a relatively lower level of differentiation and glutathione content than vector-transfected cells. The boosted differentiation was accompanied by the increased expression of both VDR protein and all the vitamin D target genes tested. The above results indicated that glutathione positively regulates VDR transcriptional activity and can mediate, at least in part, the enhancing effect of Nrf2 activators on VDD-induced differentiation of HL60 cells.

Interestingly, using similar approaches, Fujita et al. [55] have shown that BSO suppresses TNFα-stimulated osteoclast differentiation in vitro, while exogenous GSH promotes it both in cell culture and a mouse model of lipopolysaccharide-induced osteoclastogenesis. These opposite effects were associated with corresponding changes in the nuclear localization of the nuclear factor of activated T cells c1 (NFATc1), a master regulator of osteoclastogenesis, and the expression of osteoclast-specific genes [55].

### 3.2. Modulation of Glutathione Levels and Nrf2/ARE Signaling: Role of the Intracellular ROS Accumulation

Cellular Nrf2 levels are primarily regulated by Kelch-like ECH-associated protein 1 (Keap-1), a subunit of Cullin 3 (CUL3)-based E3 ubiquitin ligase. Under physiological conditions, Keap-1 physically binds Nrf2 and promotes its proteasomal degradation. ROS and electrophilic compounds, e.g., the quinone form of CA [56] and fumaric acid esters, react with cysteine SH-groups of Keap-1, triggering dissociation and cellular accumulation of Nrf2 [25,57,58].

Accordingly, treating intact or vector-transfected HL60 cells with CA or MMF increased Nrf2 protein levels and the expression of Nrf2 target genes and encoded proteins, including γGCS subunits. This correlated with an increase in the total glutathione levels. As expected, both dnNrf2-expressing and BSO-treated cells had lower basal and induced glutathione levels than the corresponding reference cells. However, only in dnNrf2-HL60 cells was this associated with impaired induction of Nrf2 and its target gene products. In contrast, BSO-treated cells exhibited enhanced induction of most tested genes and encoded proteins attributed to the Nrf2/ARE pathway. The latter could be due to the compensatory upregulation of this and other redox-sensitive regulatory pathways in response to ROS accumulation caused by pharmacological inhibition of γGCS enzymatic activity.

Unexpectedly, GEE was also found to act as a pro-oxidant, but only in dnNrf2-HL60 cells with impaired antioxidant defense, even though we used the compound at a much lower concentration (0.25 mM) compared to other studies (1.0–5.0 mM) [59,60,61,62]. This effect might be due to reductive stress, which can result in excess ROS generation [63,64,65]. Nonetheless, both dnNrf2-HL60 cells and pEF-HL60 cells exhibited enhanced induction of Nrf2-related genes and proteins when cotreated with GEE. Therefore, GSH appears to promote Nrf2/ARE activation by electrophilic agents independently of the cytosolic ROS levels, but ROS accumulation in GEE-treated dnNrf2-HL60 cells may have an additional positive effect on this pathway.

### 3.3. Modulation of c-Jun by Glutathione and the Role of AP-1 in Differentiation Enhancement

The transcription factor AP-1 is a dimeric protein complex composed of transcription factors belonging to the Jun, Fos, ATF, and Maf families, which controls the expression of various genes regulating cell proliferation, cell cycle, apoptosis, and differentiation [39,66,67]. It has also been established that AP-1 can be upregulated and activated by ROS to induce the expression of antioxidant and detoxifying enzymes [68,69]. Furthermore, c-Jun binding to the *NFE2L2* promoter was found to transcriptionally upregulate Nrf2, leading to an antioxidant effect [70]. Additionally, c-Jun can dimerize with Nrf2 and activate Nrf2/ARE-induced transcription [71].

Here, we demonstrated that consistent with our previous findings [22,29], VDDs and CA strongly cooperated in increasing c-Jun protein expression and phosphorylation. On the other hand, MMF was quite active alone, particularly in elevating P-c-Jun levels. Interestingly, another fumaric acid ester, DMF, was shown to differentially affect c-Jun in a cell type- and treatment-dependent manner. For instance, DMF upregulated c-Jun and P-c-Jun levels in macrophage migration inhibitory factor-stimulated human keratinocytes [72] but inhibited hypoxia-induced c-Jun phosphorylation in endothelial cells [73].

It was previously reported that inhibition of TPA-induced differentiation of AML cells by glutathione depletion was associated with a reversible reduction in DNA binding of AP-1 [49]. In line with these data, we found that both BSO treatment and dnNrf2 expression reduced c-Jun and P-c-Jun levels in our experimental system. Although both BSO and GEE induced ROS generation and promoted Nrf2 signaling in HL60 cells, glutathione depletion and repletion had opposite effects on c-Jun levels and phosphorylation. These results suggest that it is glutathione, and probably not other Nrf2/ARE activities, that positively regulates AP-1. By exploiting the transcription factor decoy strategy [22,30,74], we obtained evidence supporting the mediatory function of AP-1 in the differentiation-enhancing effects of the Nrf2 activators, probably via upregulating VDR/RXRα levels and transcriptional activity.

### 3.4. A Possible Role of Proteasome Inhibition in a Cooperative Upregulation of VDR and Nrf2 Protein Expression by VDDs and Nrf2 Activators

It has been reported that in 1,25D_3_-treated HL60 cells, VDR protein levels are elevated without significant changes in *VDR* gene expression [75,76]. Here, we observed a similar lack of *VDR* induction by VDDs alone and also when VDR protein levels were synergistically increased by adding Nrf2 activators. These data indicate that VDR upregulation occurred at post-transcriptional or post-translational levels. In contrast, we found that RXRα is upregulated, primarily by Nrf2 activators, at both mRNA and protein levels. This is consistent with the existence of Nrf2 binging sites (AREs) in the *RXRA* gene promoter region [77]. It was previously suggested that liganded VDR undergoes conformational changes, which prevent its proteolysis [78,79]. Indeed, several studies have demonstrated that 1,25D_3_ upregulates VDR by protecting it from proteasomal degradation [80,81,82]. Interestingly, 1,25D_3_ was also found to promote Nrf2 accumulation in bone marrow mesenchymal stem cells by inhibiting its degradation via transcriptionally repressing Keap-1 [83].

Various natural polyphenolic compounds have been shown to inhibit or activate the ubiquitin/proteasome pathway depending on multiple factors [84]. For instance, resveratrol acted as a proteasome inhibitor in breast cancer cells, inducing the accumulation of the ∆16HER2 splice variant of HER-2 [85], but promoted proteasomal degradation of Nanog in glioma stem cells [86]. These effects are also dose-dependent, e.g., curcumin increases proteasome activity at low concentrations (≤1 µM) but inhibits it at high concentrations (≥10 µM) [87].

Although both CA and MMF can activate the Nrf2/ARE pathway by inducing Keap-1-Nrf2 dissociation, which results in Nrf2 protein stabilization, CA has been shown to promote proteasomal degradation of other proteins in cancer cells (e.g., [88,89]). The major oxidized CA metabolite, carnosol, was found to target several proteins to proteasome degradation in breast cancer cells [90,91] and to directly inhibit proteasome activity in colon cancer cells [92]. DMF was shown to promote protein degradation in fibroblasts [73,93]. However, both DMF and MMF enhanced the cytotoxic effect of proteasome inhibitors in other cell types [94,95].

To the best of our knowledge, there have been no reports on regulating VDR protein proteolysis by polyphenols and fumaric acid esters. Still, CA (or its oxidized metabolites) and MMF could potentially inhibit VDR proteasomal degradation in VDD-treated HL60 cells, which enhanced VDR protein, but not mRNA, expression following combined treatments. Conversely, VDDs might cooperate with the electrophilic compounds to stabilize Nrf2. In addition, our data demonstrate that treatment with exogenous GSH alone upregulated most proteins tested in this study, suggesting a contribution of a general activation of protein synthesis or stabilization under these conditions.

### 3.5. Study Limitations, Future Directions and Potential Clinical Relevance

The synergistic enhancement of VDD-induced differentiation of AML cells by Nrf2-activating compounds has been demonstrated in different human and murine AML cell lines [14,16,17,18,19,20] and patient-derived AML blasts [21,22] as well as mouse models of AML [18,23,28]. However, the mechanistic studies of this synergy have been mostly limited to myeloblastic HL60 and promonocytic U937 cells [14,16,17,22,29]. Specifically, the current study was conducted in a single cell line (HL60) and at a single time-point (48 h), to establish a novel proof of concept for the mediatory role of glutathione in the cooperation between the VDR/RXRα and Nrf2/ARE pathways in differentiating AML cells. Our present findings further substantiate the functional link between the two pathways and support the crucial role of glutathione in cell differentiation.

AML is a highly heterogeneous malignancy with respect to a wide range of genetic mutations, chromosomal aberrations, and epigenetic abnormalities. This heterogeneity strongly affects the drug responsiveness of AML blasts, including the sensitivity to differentiation inducers, such as VDDs [9]. Therefore, further extensive research is required to fully characterize the mechanisms of the sensitization of AML cells to VDDs by electrophilic Nrf2 activators. The roles of Nrf2/ARE and other signaling and transcriptional pathways in the synergy between these compounds need to be investigated in various established AML cell lines and patient-derived blasts carrying different genetic and epigenetic defects. Low sensitivity of cancer cells to VDDs precludes using these promising compounds in clinical oncology/hematology. Thus, elucidating the modes of the interplay between VDR/RXRα, Nrf2/ARE and AP-1 pathways would enhance the translational potential and future clinical significance of the current findings.

High Nrf2 expression and/or activity in cancer cells, including AML blasts, is linked to increased cell proliferation, survival and chemoresistance [96,97,98]. However, based on our previous and present findings, upregulation of the Nrf2/ARE pathway could be exploited for VDD-based differentiation therapy in the corresponding subset of AML patients. Particularly, we suggest conducting a clinical trial of combined treatment with the clinically approved repurposed drugs paricalcitol (*Zemplar*), indicated for the prevention and treatment of secondary hyperparathyroidism associated with chronic kidney disease, and MMF (*Bafiertam*), used in relapsing forms of multiple sclerosis.

Another subset of patients with nonAPL AML who can benefit from differentiation therapy carries recurrent mutations in *IDH1* or *IDH2*. These mutations have been shown to block normal myeloid differentiation via the production of the oncometabolite, *R*-2-hydroxyglutarate from α-ketoglutarate [99,100]. Treatment with the recently approved mutant IDH1 and IDH2 inhibitors Ivosidenib and Enasidenib, respectively, causes clinically evident myeloid differentiation and even differentiation syndrome, a rare life-threatening complication in patients undergoing differentiation therapy [7,8,101]. These findings hold promise for developing mechanism-based differentiation-inducing treatment strategies for nonAPL AML.

## 4. Materials and Methods

### 4.1. Materials

Carnosic acid (>98%) was obtained from ShenZhen Ipure Biological Import and Export Co., Ltd. (Shenzhen, China). The 1,25D_3_ was purchased from Selleck Chemicals (Houston, TX, USA). Monomethyl fumarate (MMF) and DMSO were obtained from Sigma Chemical Co. (St. Louis, MO, USA). Paricalcitol and glutathione ethyl ester (GEE) were from Cayman Chemical (Ann Arbor, MI, USA). The antibodies against VDR (D-6 and C-20), RXRα (D-20), NQO-1 (A-5), ɣ-GCSc (H-5), ɣ-GCSm (E-4), TrxR1 (B-2), and 2′,7′-dichlorofluorescein-diacetate (DCFH-DA) were procured from Santa Cruz Biotechnology Inc. (Dallas, TX, USA). Antibodies against P-c-Jun (54B3), c-Jun (60AB), and HO-1 (D60611) were acquired from Cell Signaling Technology (Danvers, MA, USA). Antibodies against Nrf2 (MAB3925) and calreticulin (PA3-900) were purchased from R&D Systems (Minneapolis, MN, USA) and Thermo Fisher Scientific (Waltham, MA, USA), respectively. Peroxidase-conjugated AffiniPure donkey anti-rabbit and sheep anti-mouse IgG antibodies were bought from Jackson ImmunoResearch Laboratories, Inc. (West Grove, PA, USA). L-buthionine sulfoximine (BSO) was obtained from Merck-Sigma-Aldrich (Rehovot, Israel). Hanks’ buffered salt solution (HBSS), penicillin, streptomycin, and HEPES buffer were from IMBH (Beth Haemek, Israel). RPMI 1640 medium and heat-inactivated fetal bovine serum (FBS) were purchased from Gibco-Invitrogen (Carlsbad, CA, USA). Stock solutions of 1,25D_3_ and paricalcitol (2.4 mM), CA (10 mM), and MMF (50 mM) were prepared in absolute ethanol. The precise concentration of 1,25D_3_ in ethanol was verified spectrophotometrically at 264 nm (ε = 19,000).

### 4.2. Cell Culture and Stable Transfection

HL60 myeloblastic leukemia cells (ATCC-CCL-240) were grown in RPMI 1640 medium supplemented with 10% fetal calf serum (FCS), penicillin (100 U/mL), streptomycin (0.1 mg/mL), and 10 mM HEPES (pH = 7.4) in a humidified atmosphere of 95% air and 5% CO_2_ at 37 °C. The expression vector for dnNrf2, which lacks the transactivation domain residues 1–392 in the NH_2_-terminal portion of the protein, and the empty vector (pEF) were generously provided by Dr. Jawed Alam (Louisiana State University Medical Center, New Orleans, LA, USA). Both plasmids carried the neomycin (*neoR*) resistance gene. Stable nucleofection was performed by Dr. Irene Bobilev (Ben Gurion University of the Negev), as described previously [22]. Briefly, HL60 cells (1 × 10^7^ cells/mL) were mixed with 1 µg plasmid in Cell Line Nucleofector Solution V and transfected in an Amaxa Nucleofector (Lonza, Cologne, Germany) according to the manufacturer’s protocol (program T-19).

### 4.3. Determination of Cell Differentiation Markers

Cells were seeded at 1 × 10^5^ cells/mL and treated with test agents or vehicle (<0.2% ethanol) for 48 h. Cell numbers and viability were determined using the trypan blue exclusion assay via enumeration in a Vi-Cell XR cell viability analyzer (Beckman Coulter Inc., Fullerton, CA, USA). Aliquots of 5 × 10^5^ cells were harvested, washed with PBS, and incubated for 45 min at room temperature with 0.3 µL MO1-FITC and 0.3 µL MY4-RD1 (Beckman Coulter) to determine the expression of myeloid surface antigens CD11b and CD14, respectively, via flow cytometry as described previously [20,24]. For each analysis, 10,000 events were recorded, and the data were processed using Kaluza software, version 2.1 (Beckman Coulter).

### 4.4. Determination of Intracellular Levels of Reactive Oxygen Species

Cytosolic ROS levels were determined using the oxidation-sensitive fluorescent probe DCFH-DA. Intracellular ROS oxidize this probe to a highly fluorescent compound, DCF. Following treatments with the specified compounds at the indicated time points, cells were washed with HBSS containing 10 mM HEPES buffer (pH = 7.4). Subsequently, cells were stained with 5 μM DCFH-DA for 15 min at 37 °C in the dark using a shaking water bath and washed with HEPES-buffered HBSS. For the positive control, DCFH-DA-loaded cells were treated with 0.5 mM H_2_O_2_ for 15 min. Untreated and unstained cells served as the negative control. In the experiment reported in Figure 5c,d, DCFH-DA-loaded cells were divided into two groups and incubated with vehicle (HBSS) or 10 µM of H_2_O_2_ for 15 min, followed by washing with HEPES-buffered HBSS. The DCF fluorescence intensity was measured via flow cytometry, recording 10,000 events for each analysis. Data were analyzed using Kaluza Analysis Software version 2.1 (Beckman Coulter) [59].

### 4.5. Preparation of Whole Cell Lysates and Western Blotting

Cells were seeded at 1 × 10^5^ cells/mL and incubated with test agents or vehicle (<0.2% ethanol) for 48 h. Preparation of whole cell lysates and Western blotting analysis were performed as described previously [20]. Briefly, cells were lysed in a buffer containing 1% (*v*/*v*) Triton X-100 at 4 °C, subjected to SDS-PAGE, and electroblotted into nitrocellulose membranes. The membranes were exposed to primary antibodies overnight at 4 °C. Blots were washed and incubated with horse-radish peroxidase-conjugated secondary antibodies. Membranes were stripped and reprobed for calreticulin, the internal loading control. The protein bands were visualized using the WESTAR ANTARES chemiluminescent substrate for Western blotting (Cyanagen, Bologna, Italy). The absorbance of each band was determined using the Image Quant LAS 4000 system (GE Healthcare, Little Chalfont, UK).

### 4.6. RNA Extraction, cDNA Synthesis, and RT-qPCR

Total RNA was purified from cell cultures according to the manufacturer’s instructions using an RNA extraction kit (GENEzol^TM^ TriRNA Pure Kit+DNASE I; Geneaid, New Taipei City, Taiwan). A micro-volume spectrophotometer (NanoDrop; Wilmington, DE, USA) was used for RNA quantification. First-strand cDNA was generated using reverse transcriptase kit (qScript cDNA synthesis kit; QUANTA Biosciences; Gaithersburg, MD, USA) using random oligo (dT) after a sample concentration was normalized. Quantitative cDNA amplification was performed via real-time PCR (StepOne Real-Time PCR System; Thermo Fisher Scientific; Wilmington, DE, USA) using qPCRBIO Fast qPCR SyGreen Blue Mix from Tamar Laboratory Supplies Ltd. (Mevaseret Zion, Israel). Relative mRNA expression levels were determined using the 2^−(ΔΔCt)^ formula, where ΔCt is Ct (_target gene_)—mean of Ct (_reference genes_). The reference gene used in this study was *GAPDH*. Each experiment was performed using three biological replicates, each assayed in triplicate. Primers for qPCR were synthesized by Hy Laboratories Ltd. (Rehovot, Israel) and Tamar Laboratory Supplies Ltd. (Mevaseret Zion, Israel).

The primer sequences used in this study were as follows:
CAMP FRGCTAACCTCTACCGCCTCCTCAMP REVGGTCACTGTCCCCATACACCCD14 FRCAACCTAGAGCCGTTTCTAAAGCCD14 REVGCGCCTACCAGTAGCTGAGCYP24A1 FRGGAAGTGATGAAGCTGGACAACACYP24A1 REVCTCATACAACACGAGGCAGATACGAPDH FRCATGAGAAGTATGACAACAGCCTGAPDH REVAGTCCTTCCACGATACCAAAGTGCLC FRGGAGGAAACCAAGCGCCATGCLC REVCTTGACGGCGTGGTAGATGTGCLM FRGGAAGAAGTGCCCGTCCAGCLM REVCTGAACAGGCCATGTCAACTHO-1 FRAAGACTGCGTTCCTGCTCAAHO-1 REVGGTCCTTGGTGTCATGGGTCITGAM FRCTGTCTGCCAGAGAATCCAGTGITGAM REVGAGGTGGTTATGCGAGGTCTTGNQO1 FRAAAGAAGGCCATCTGAGCCCNQO1 REVCCAGGCGTTTCTTCCATCCTNrf2 FRCCTTGTCACCATCTCAGGGGNrf2 REVTGGGGTTTTCCGATGACCAGTXNRD1 FRACGTTACTTGGGCATCCCTGTXNRD1 REVAGAAATCCAGCGCACTCCAAVDR FRGACCTGTGGCAACCAAGACTVDR REVAATCAGCTCCAGGCTGTGTC

### 4.7. Total Glutathione Assay

Cells (2 × 10^6^) were collected by centrifugation (1000× *g* for 5 min), washed with ice-cold PBS, and resuspended in 200 µL of 5% 5-sulfosalicylic acid. After 15 min on ice with intermittent vortexing, the suspension was centrifuged at 16,000× *g* for 5 min to remove protein precipitates. Total glutathione was determined in the supernatants using the glutathione reductase recycling assay [22,24]. The rates of 5-thio-2-nitrobenzoic acid (TNB) formation were measured kinetically at 412 nm for 30 min using a VersaMax microplate spectrophotometer (Molecular Devices, Sunnyvale, CA, USA).

### 4.8. Cell Treatment with AP-1 Decoy Oligodeoxynucleotides

HL60 cells were preincubated for 24 h with 10 µM double-stranded phosphorothioate oligodeoxynucleotide containing the proximal binding site for AP-1 (TPA-response element; TRE) from the promoter region of hVDR (−77 to −97 relative to the transcription start site; 5′-CTG GCA AGA GAG **GA**C TGG ACC-3′) or its mutant (5′-CTG GCA AGA GAG **TG**C TGG ACC-3′) in the complete culture medium, followed by treatment with test agents for an additional 48 h [22]. The oligonucleotides were synthesized by Integrated DNA Technologies, lnc. (Coralville, IA, USA).

### 4.9. Transient Transfection and Reporter Gene assay

Cells were harvested after 48 h of culture, washed once in a preheated growth medium at 37 °C, and resuspended at 5 × 10^5^ cells/mL. Four hundred μL of the cell suspension were transferred to 24-well plates, followed by cotransfection with 0.8 µg VDREx6-Luc luciferase reporter plasmid and 0.2 µg *Renilla* luciferase (pRL-null) reporter plasmid (internal control) using jetPEI reagent (Polyplus Transfection, Illkrich, France). Four hours later, cells were preincubated for 24 h with either a vehicle, 10 µM TRE-ODN, or mutant TRE (mTRE)-ODN. Subsequently, they were treated for an additional 48 h with 1 nM 1,25D_3_, 10 µM CA, or 50 µM MMF, alone or in combination. Firefly and *Renilla* luciferase activities were then measured using the dual-luciferase reporter assay system (Promega, Madison, WI, USA) in accordance with the manufacturer’s instructions. Luminescence was determined using a Turner 20/20 luminometer (Turner BioSystems, Sunnyvale, CA, USA). Data are expressed as firefly luciferase-to-*Renilla* luciferase ratios (RLU) [22]. The VDREx6-Luc reporter plasmid was gifted by Dr. David G. Garner (University of California, San Francisco, CA, USA). The pRL-null vector was purchased from Promega (Madison, WI, USA).

### 4.10. Statistical Analysis

All experiments were performed at least three times. Statistically significant differences between the two experimental groups were assessed using one-way ANOVA followed by Tukey’s multiple comparisons test. Data are presented as the mean ± SD. *p* < 0.05 was considered statistically significant. The synergy between the effects of two compounds (A and B) was assumed if the effect of their combination (AB) was larger than the sum of their individual effects (AB > A + B), the data being compared after subtraction of the respective control values from A, B, and AB [17]. The statistical analyses were performed using GraphPad Prism 6.0 software (GraphPad Software, San Diego, CA, USA).

## 5. Conclusions

This study addressed the mechanism of the synergy between VDDs and Nrf2 activators in inducing monocytic differentiation of AML cells. Using glutathione depletion and repletion approaches as well as impairment of Nrf2 activity, we obtained evidence that glutathione mediates, at least in part, the interplay between the Nrf2/ARE and vitamin D signaling pathways, likely through the positive regulation of AP-1. Upregulated AP-1 appears to be essential for enhancing VDD-induced differentiation by Nrf2 activators via increasing VDR/RXRα protein levels and transcriptional activity. Glutathione may also promote Nrf2/ARE activity, which appears to contribute to potentiating VDR signaling.

High expression and persisted activation of Nrf2 in AML blasts promote a more malignant phenotype and resistance to chemotherapy [96,97,98]. Still, our results support the notion that a dose-sparing combination therapy with low-calcemic VDDs and Nrf2 activators may be advantageous for a subset of AML patients with upregulated Nrf2 signaling. This mild treatment strategy may also be explored as part of therapeutic regimens in older patients who are unfit for standard intensive chemotherapy.

## Data Availability

All datasets generated for this study are included in the manuscript and Supplementary Materials.

## Supplementary Materials

The following supporting information can be downloaded at: https://www.mdpi.com/article/10.3390/ijms25042284/s1.
