# Supplementary material for: Roles of Glutathione and AP-1 in the Enhancement of Vitamin D-Induced Differentiation by Activators of the Nrf2 Signaling Pathway in Acute Myeloid Leukemia Cells
Source: Int J Mol Sci. 2024 Feb 14;25(4):2284. doi: 10.3390/ijms25042284 (PMC10889780; doi:10.3390/ijms25042284)
Supplement: Supplementary file 1 [file ijms-25-02284-s001.zip › Original WBs new-ijms-2816601.pdf]

Fig 3a

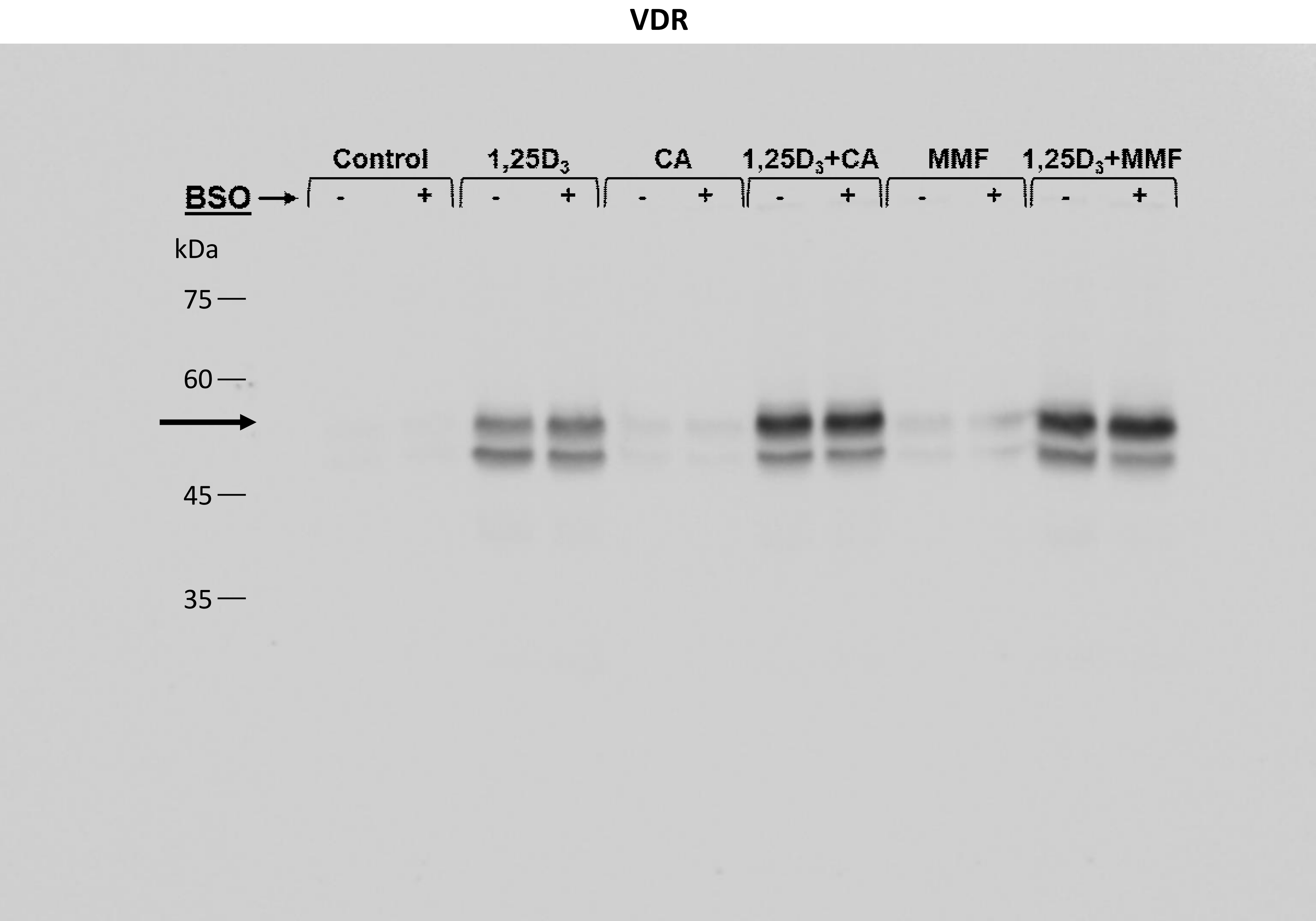

Fig 3a

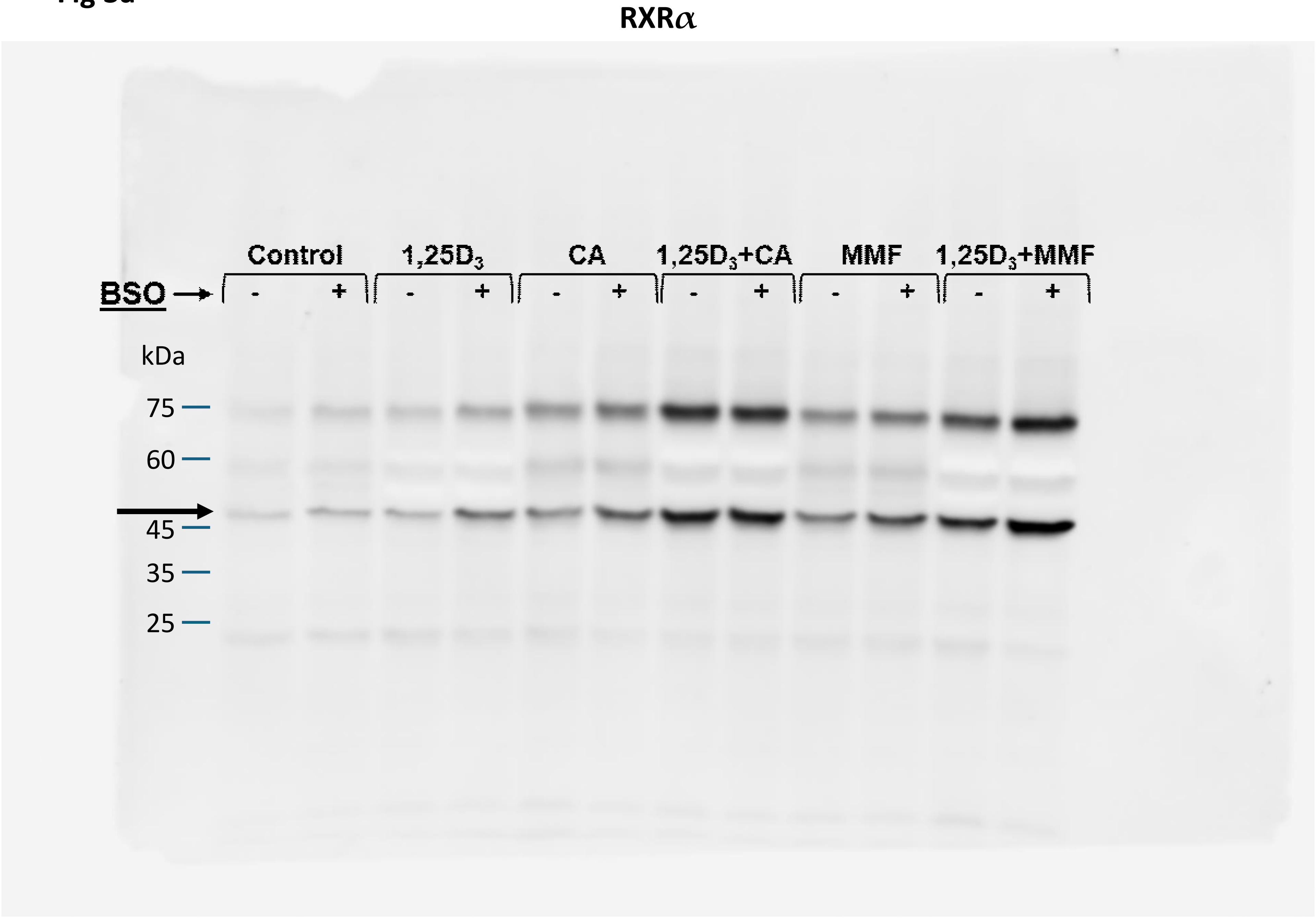
RXRα
Control
1,25D3
CA
1,25D3+CA
MMF
1,25D3+MMF
BSO
- + - + - + - + - + - +
kDa
75
60
45
35
25

Fig 3a

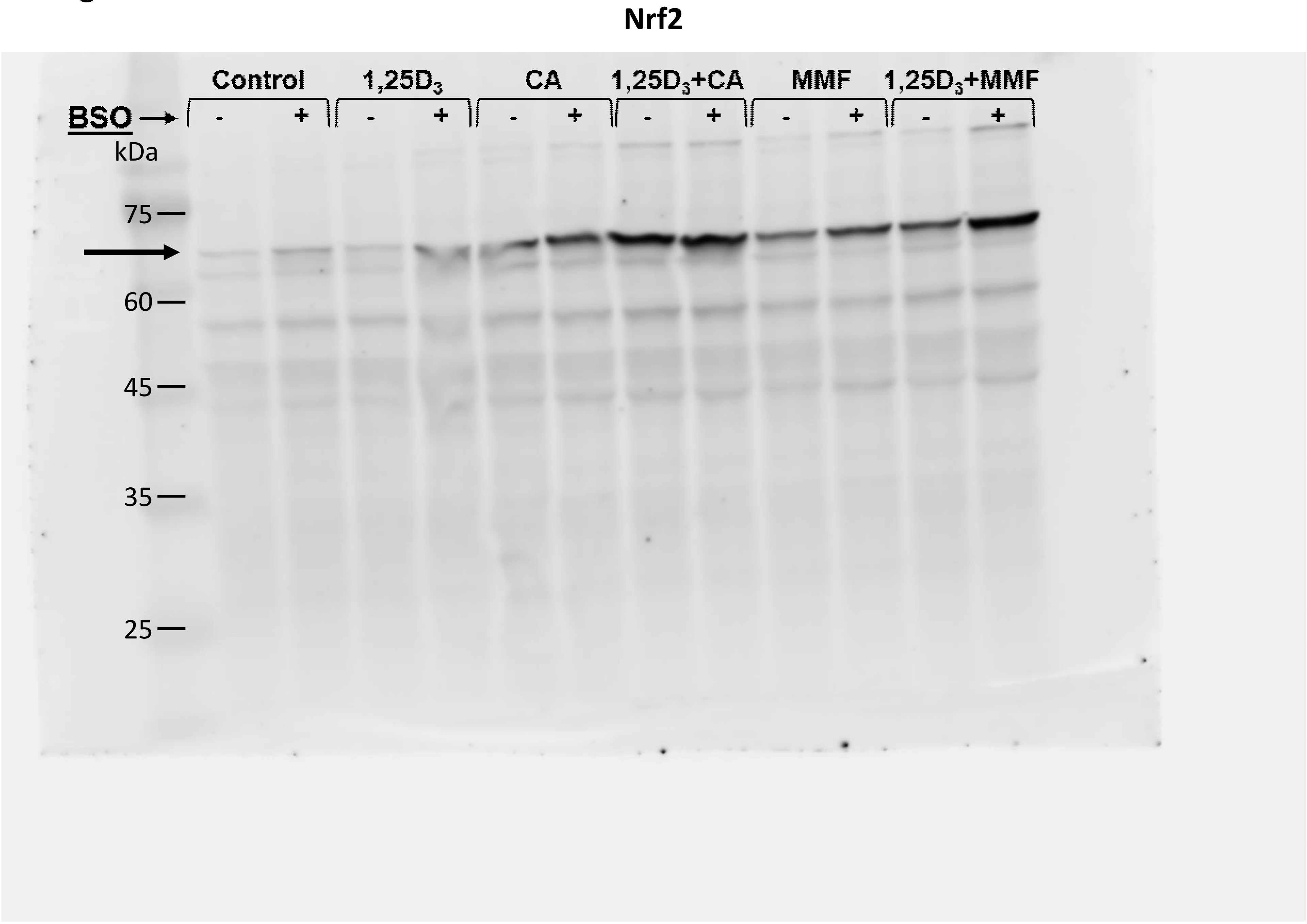
Nrf2
Control
1,25D3
CA
1,25D3+CA
MMF
1,25D3+MMF
BSO
- + - + - + - + - + - +
kDa
75
60
45
35
25

Fig 3a

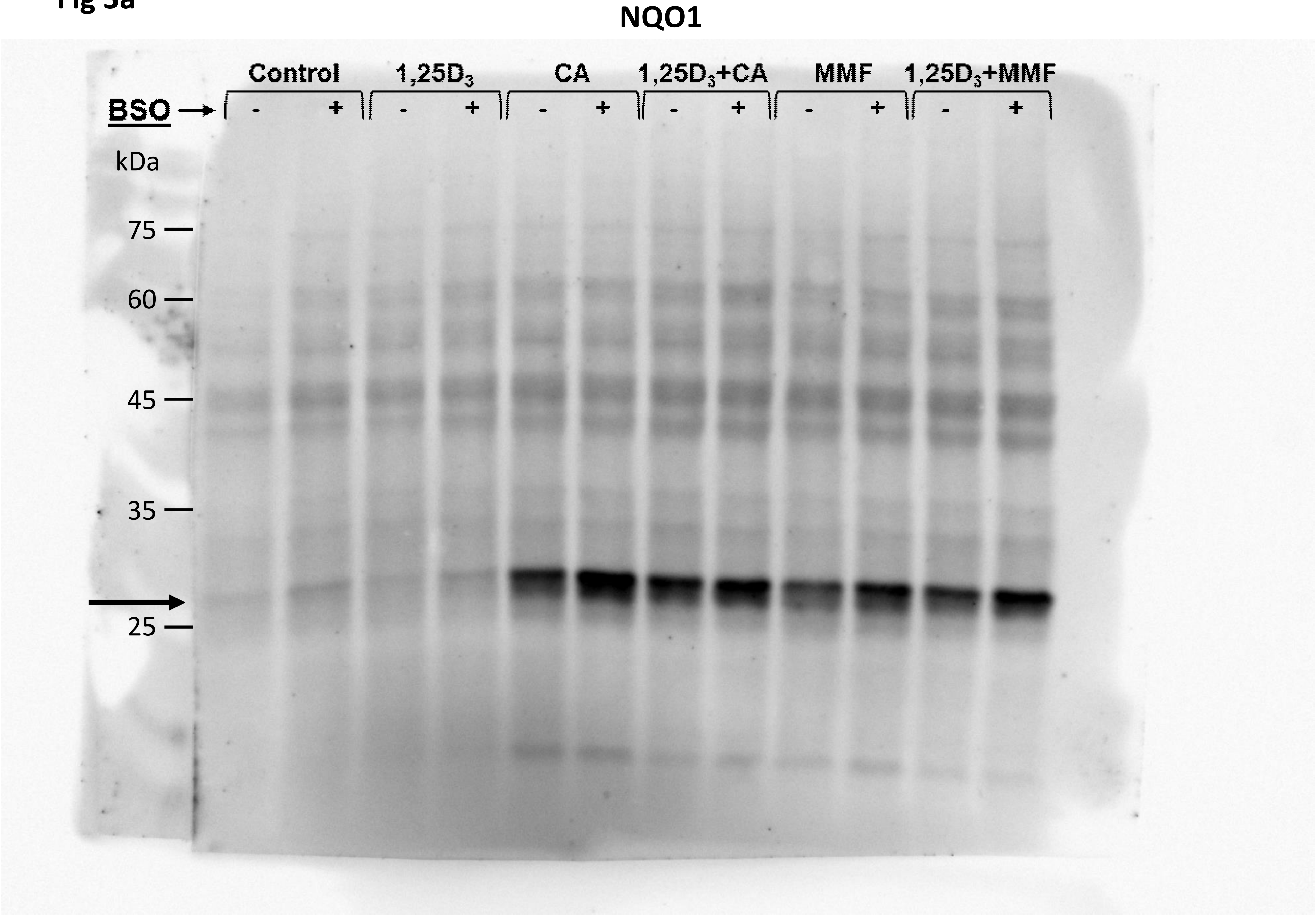

NQO1
Control
1,25D3
CA
1,25D3+CA
MMF
1,25D3+MMF
BSO
- + - + - + - + - + - +
kDa
75
60
45
35
25

Fig 3a

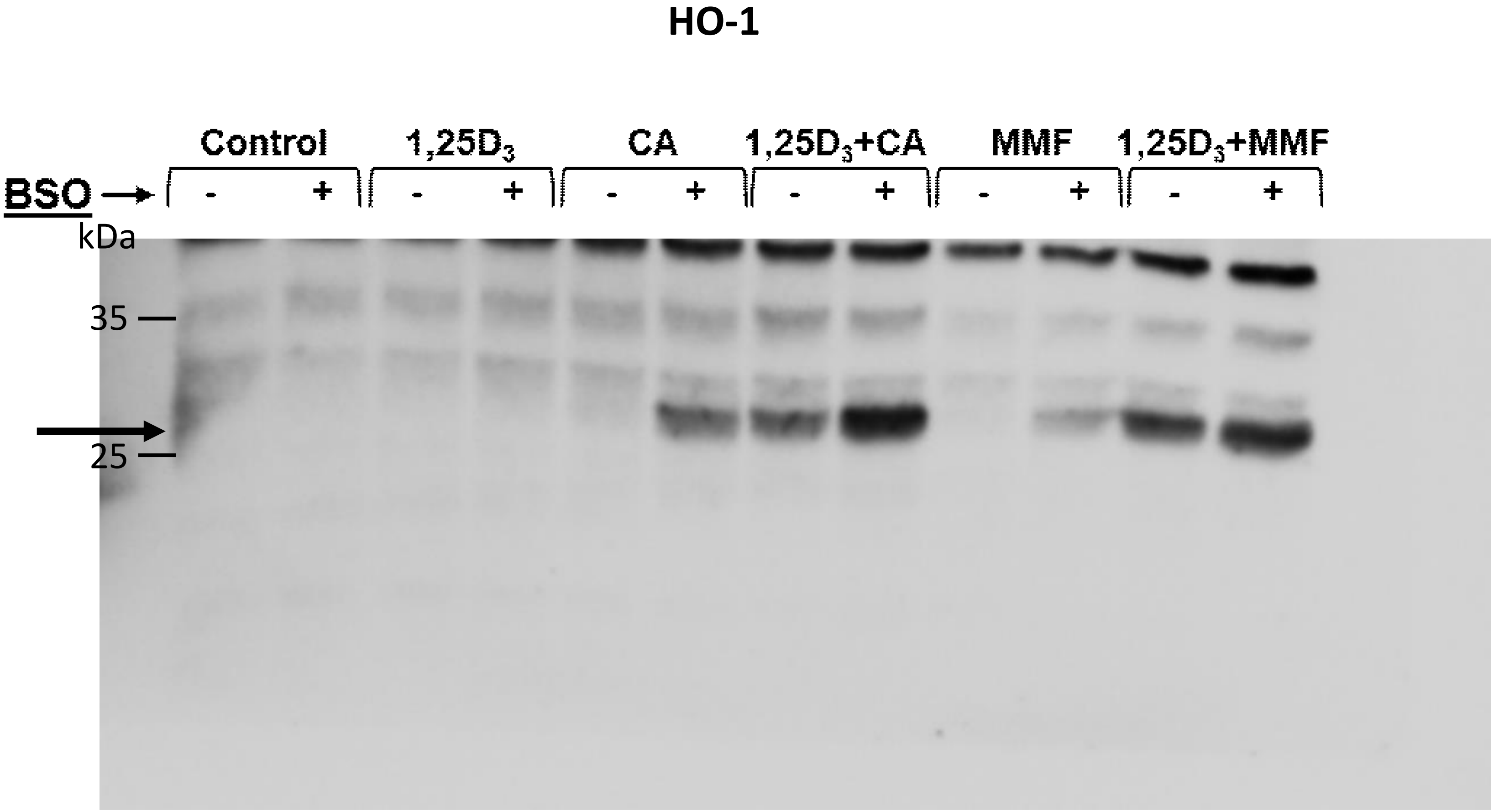

Fig 3a

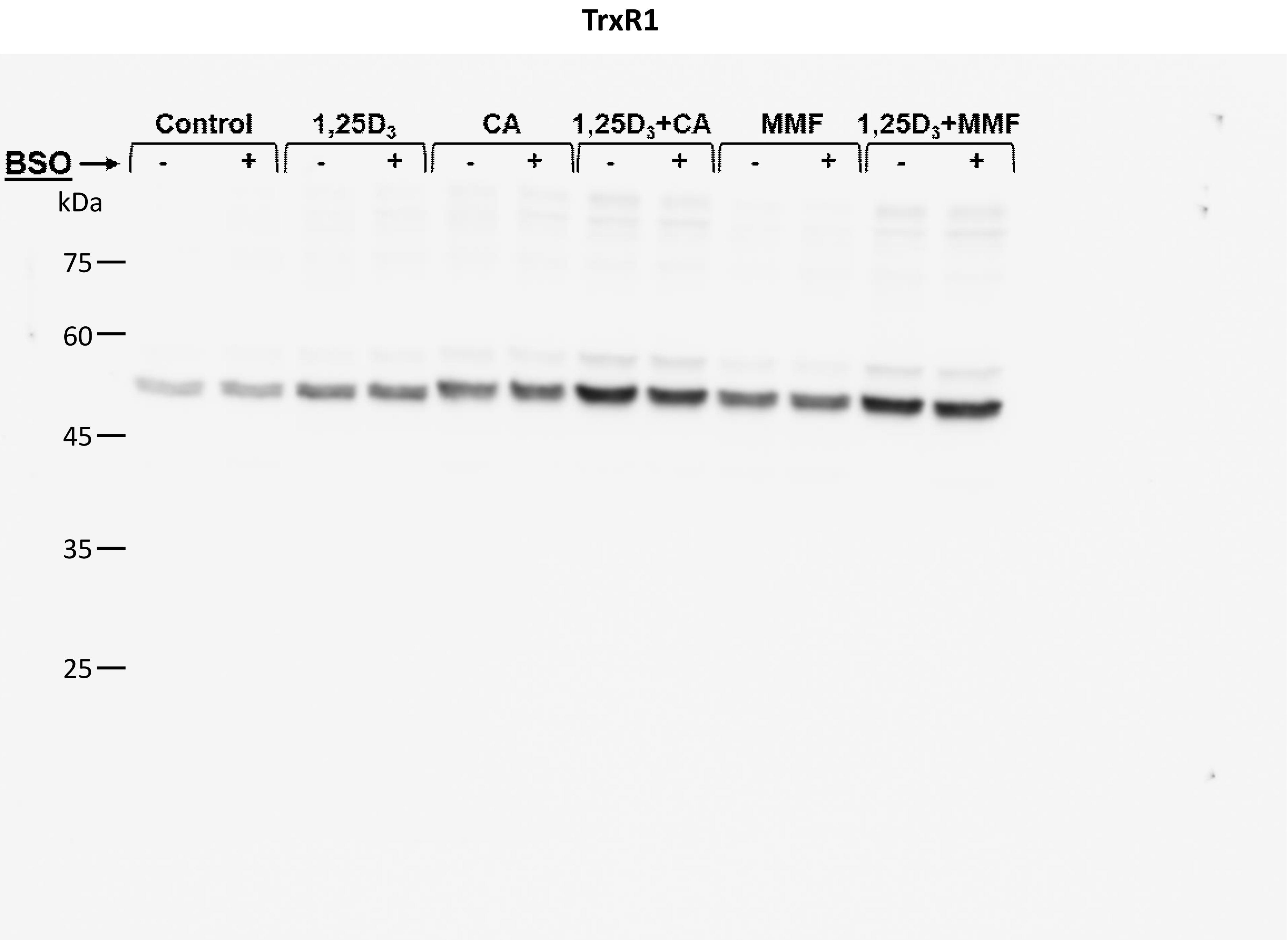
TrxR1
Control
1,25D3
CA
1,25D3+CA
MMF
1,25D3+MMF
BSO
- + - + - + - + - + - +
kDa
75
60
45
35
25

Fig 3a

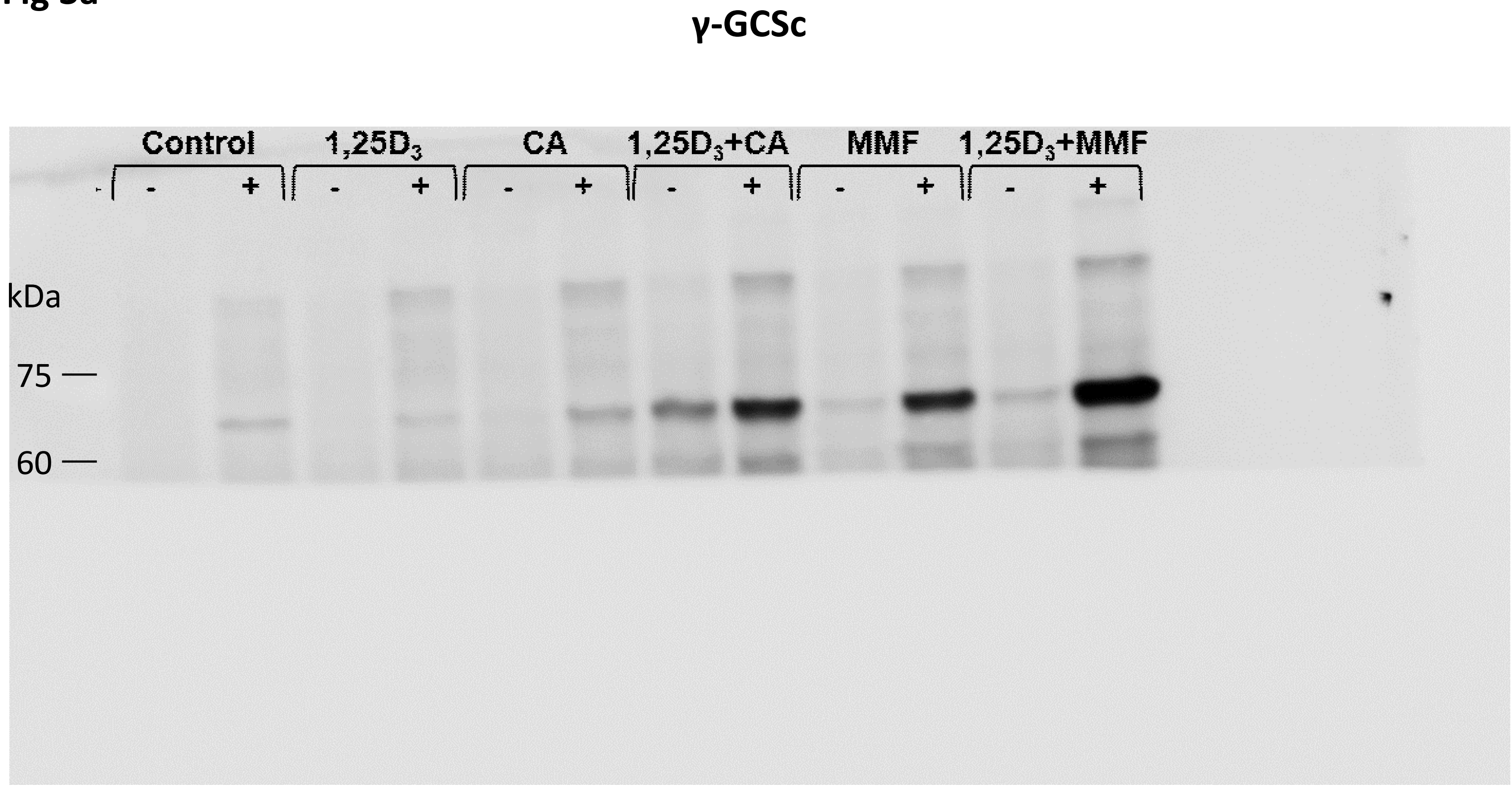

Fig 3a

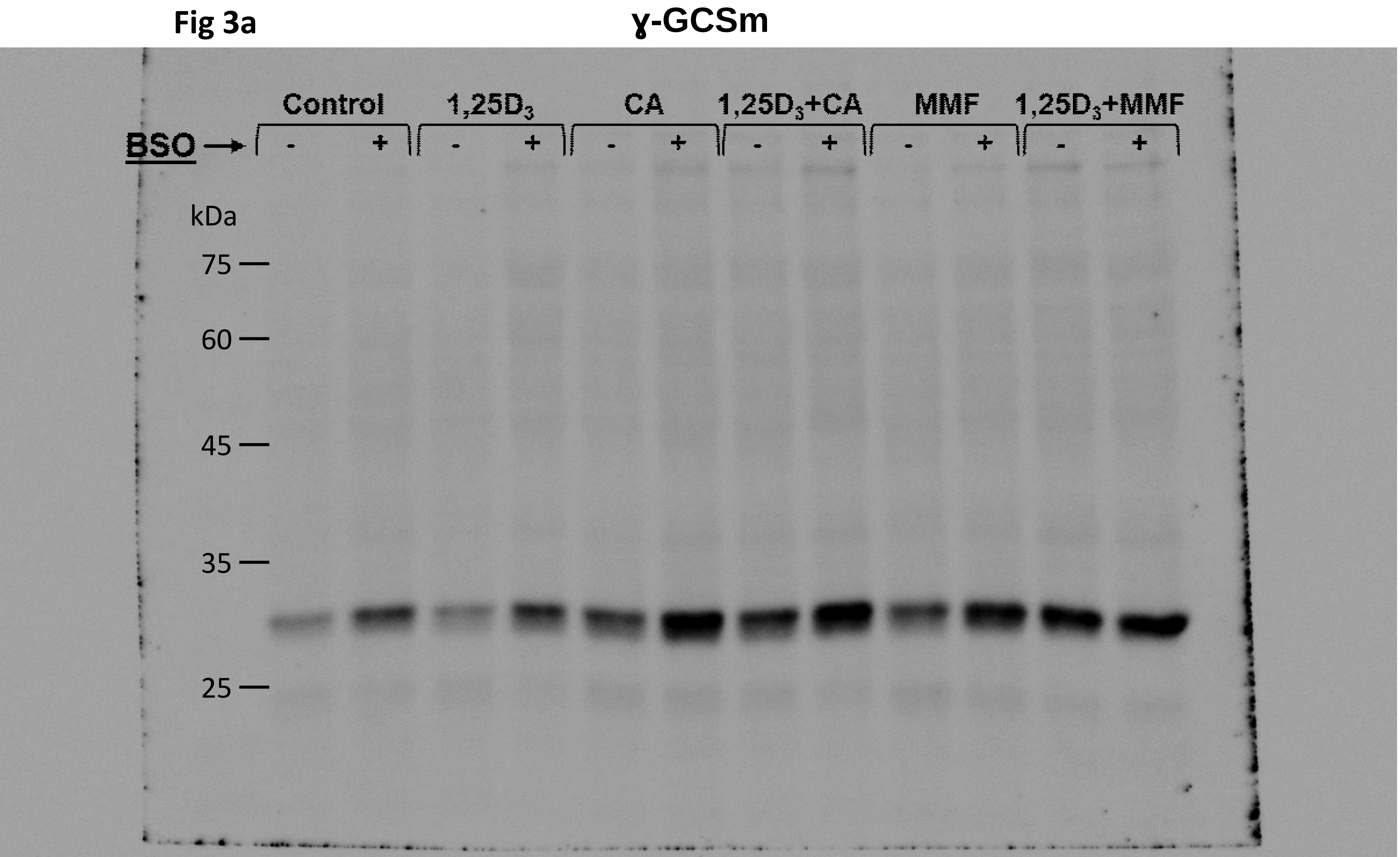
γ-GCSm
Control
1,25D3
CA
1,25D3+CA
MMF
1,25D3+MMF
BSO
- +
- +
- +
- +
- +
- +
kDa
75
60
45
35
25

Fig 3a

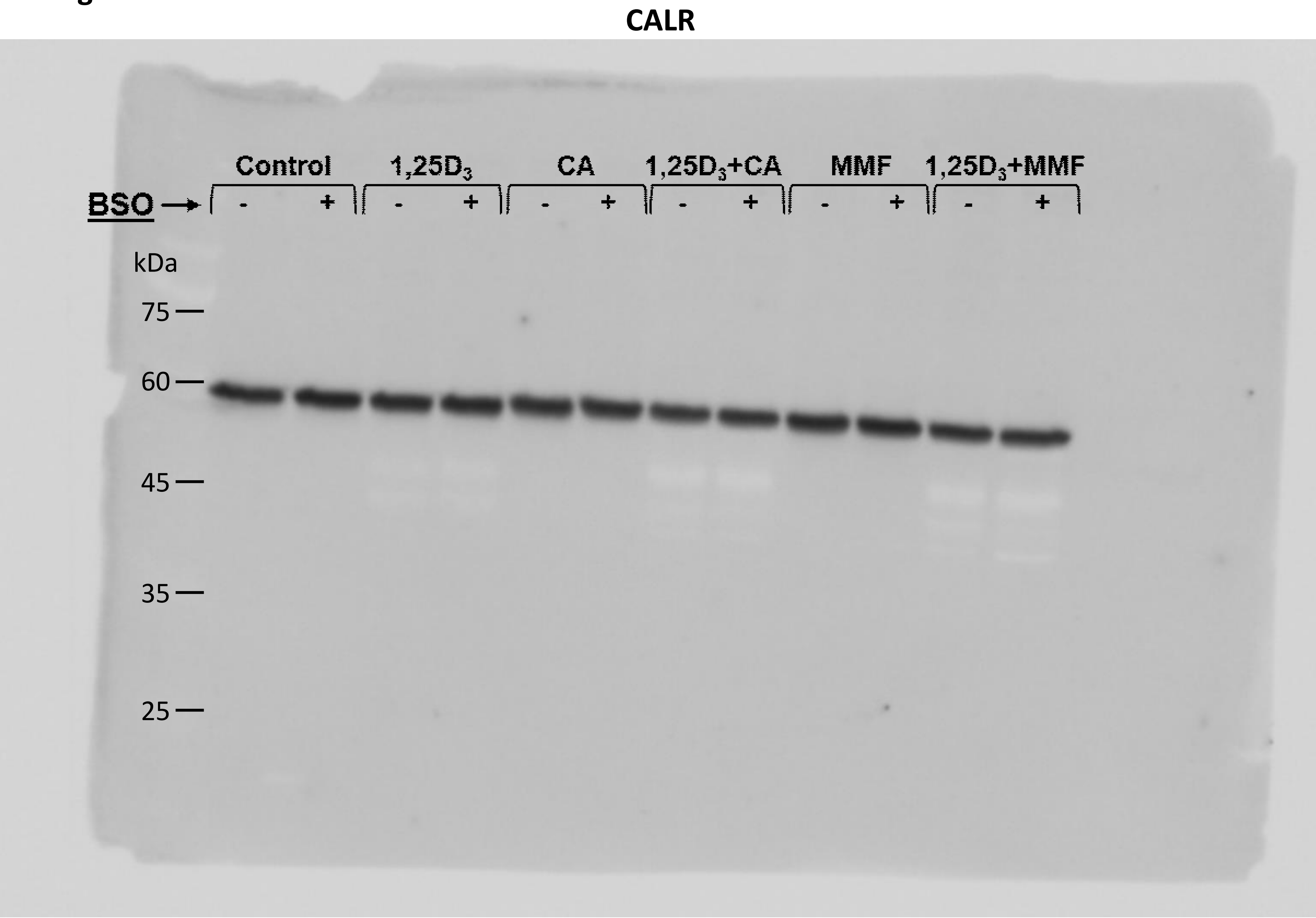

CALR
Control
1,25D3
CA
1,25D3+CA
MMF
1,25D3+MMF
BSO
- +
- +
- +
- +
- +
- +
kDa
75
60
45
35
25

Fig 3j

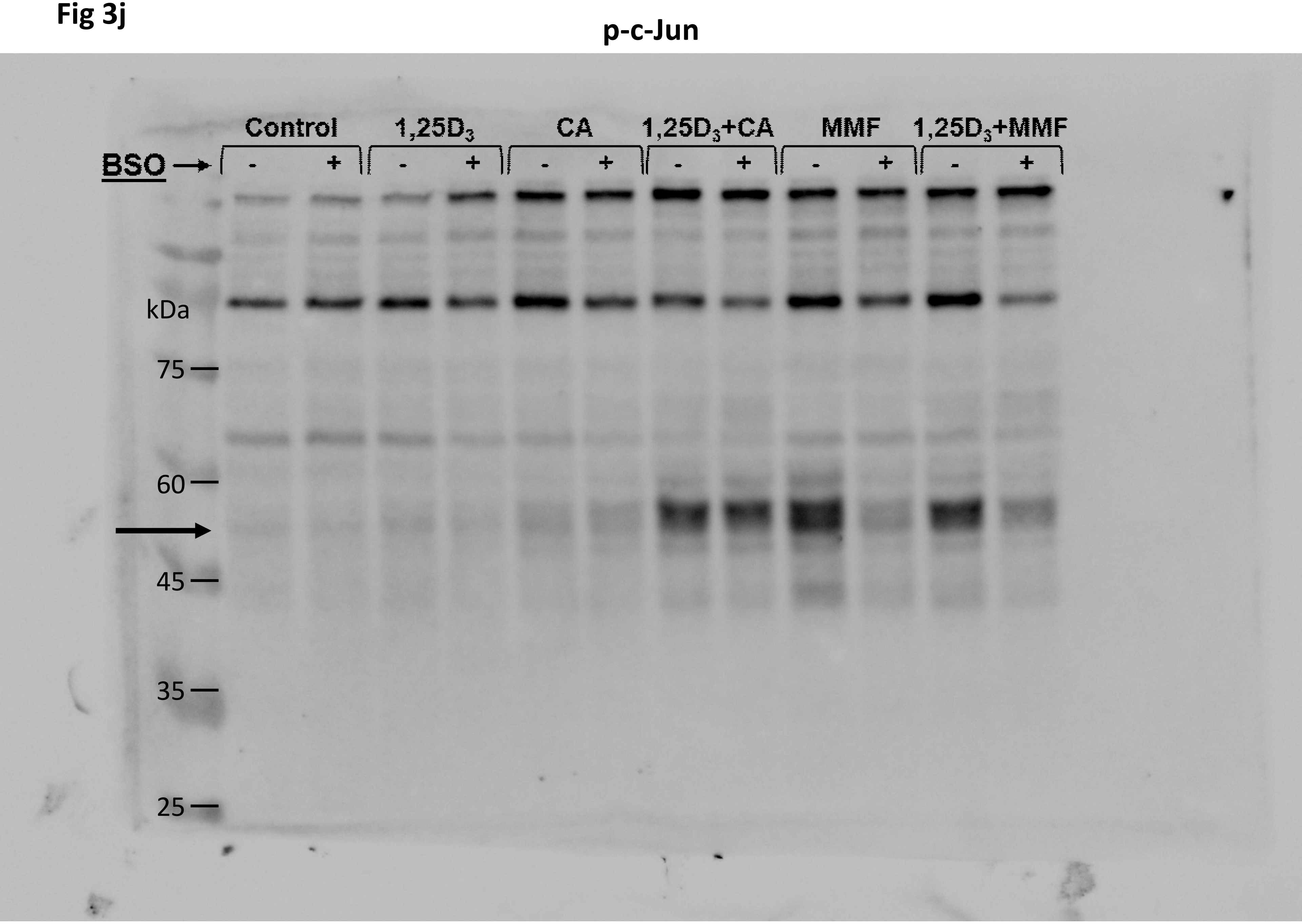
p-c-Jun
Control
1,25D3
CA
1,25D3+CA
MMF
1,25D3+MMF
BSO
-
+
kDa
75
60
45
35
25

Fig 3j

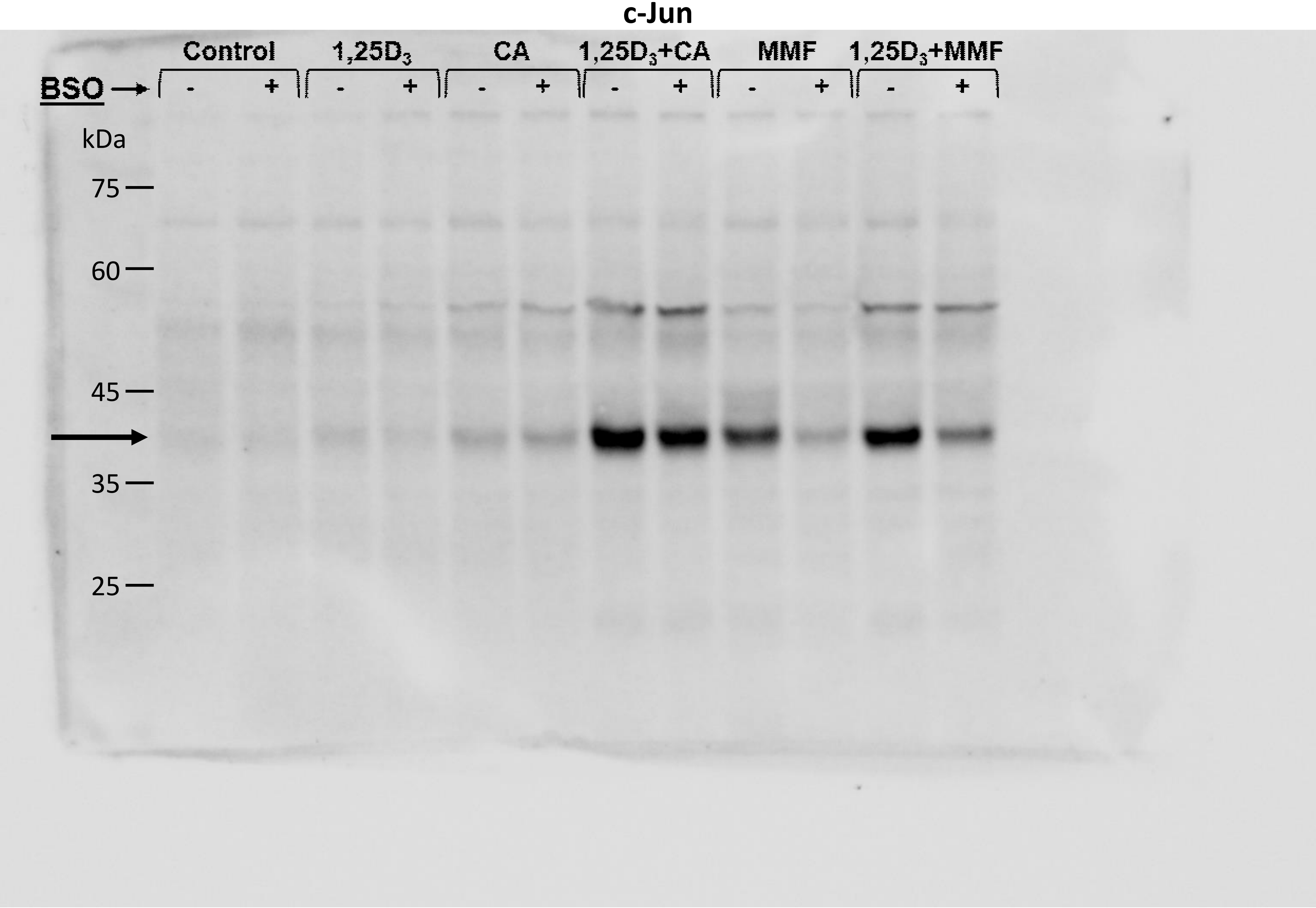
c-Jun
Control
1,25D3
CA
1,25D3+CA
MMF
1,25D3+MMF
BSO
- + - + - + - + - + - +
kDa
75
60
45
35
25

Fig 3j

CALR

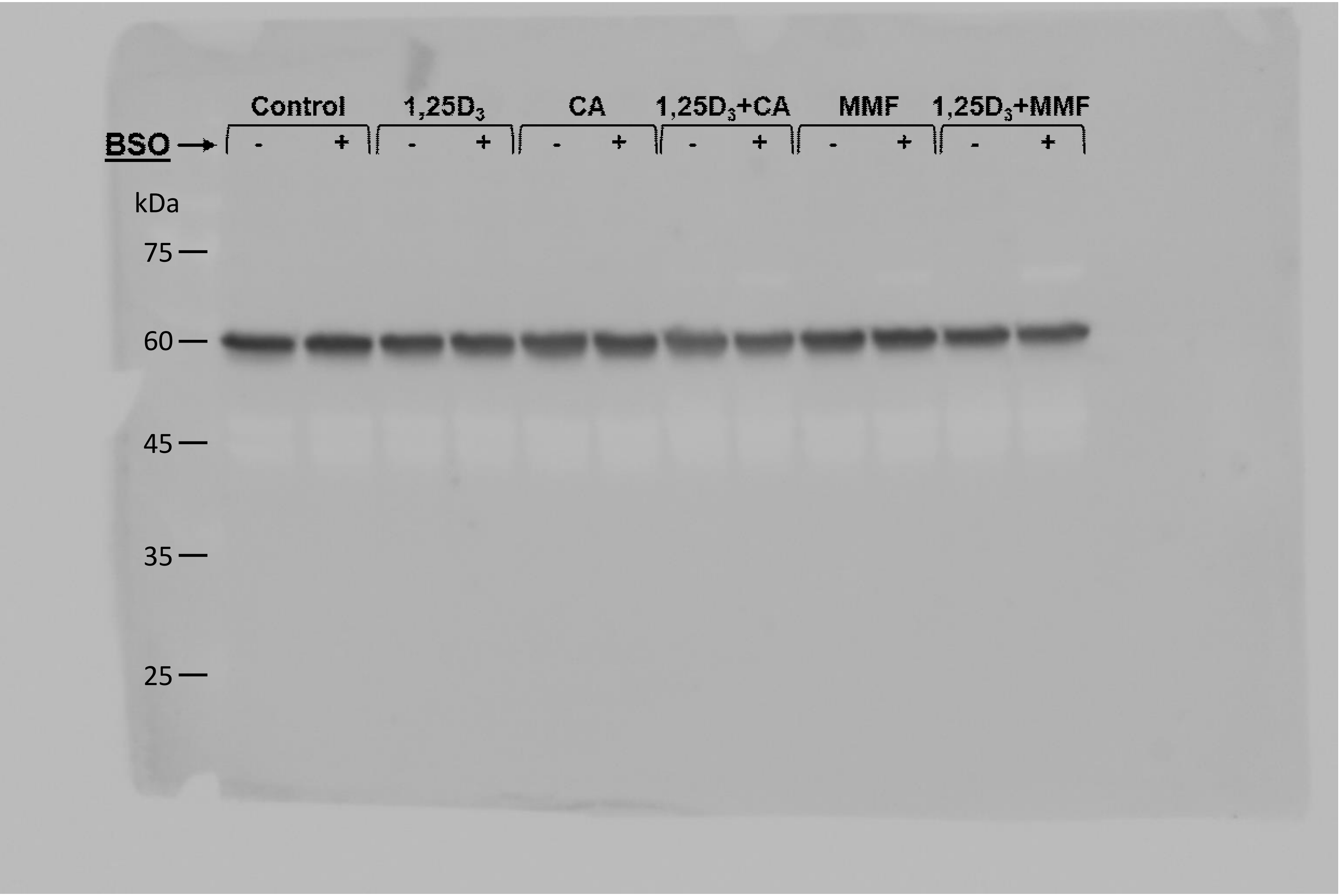

Fig 7a -pEF-HL60

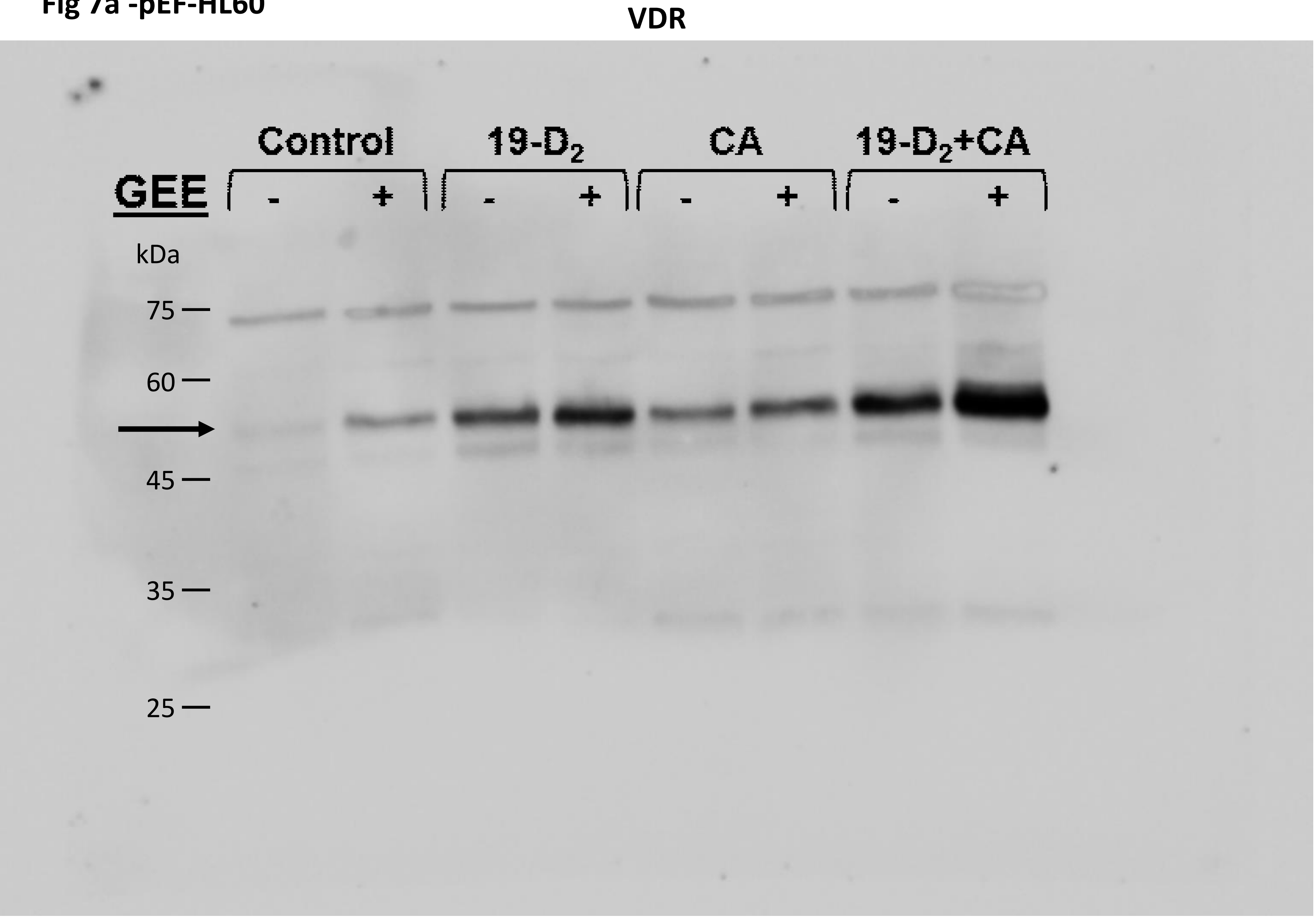

Fig 7a -pEF-HL60

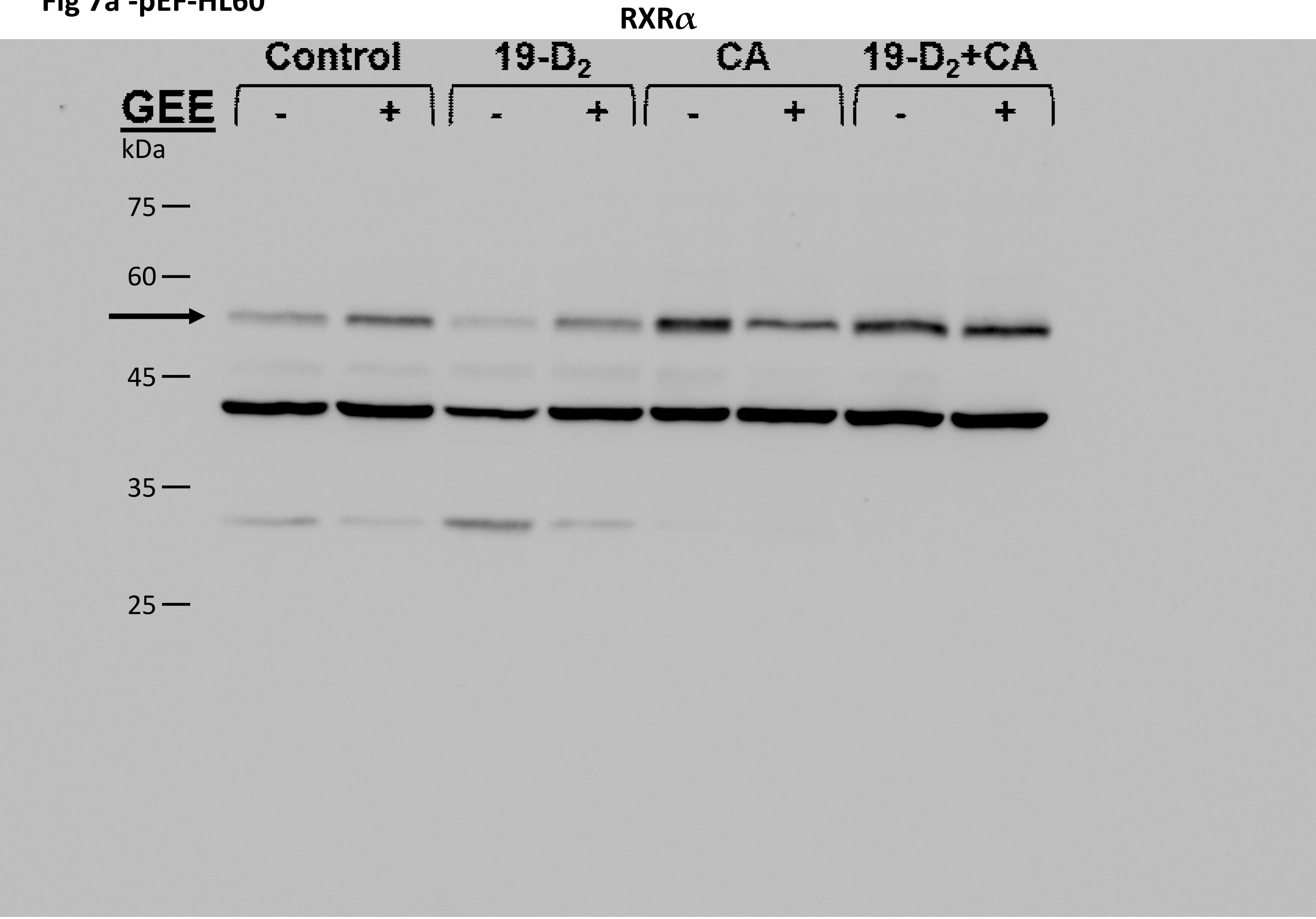

Fig 7a -pEF-HL60

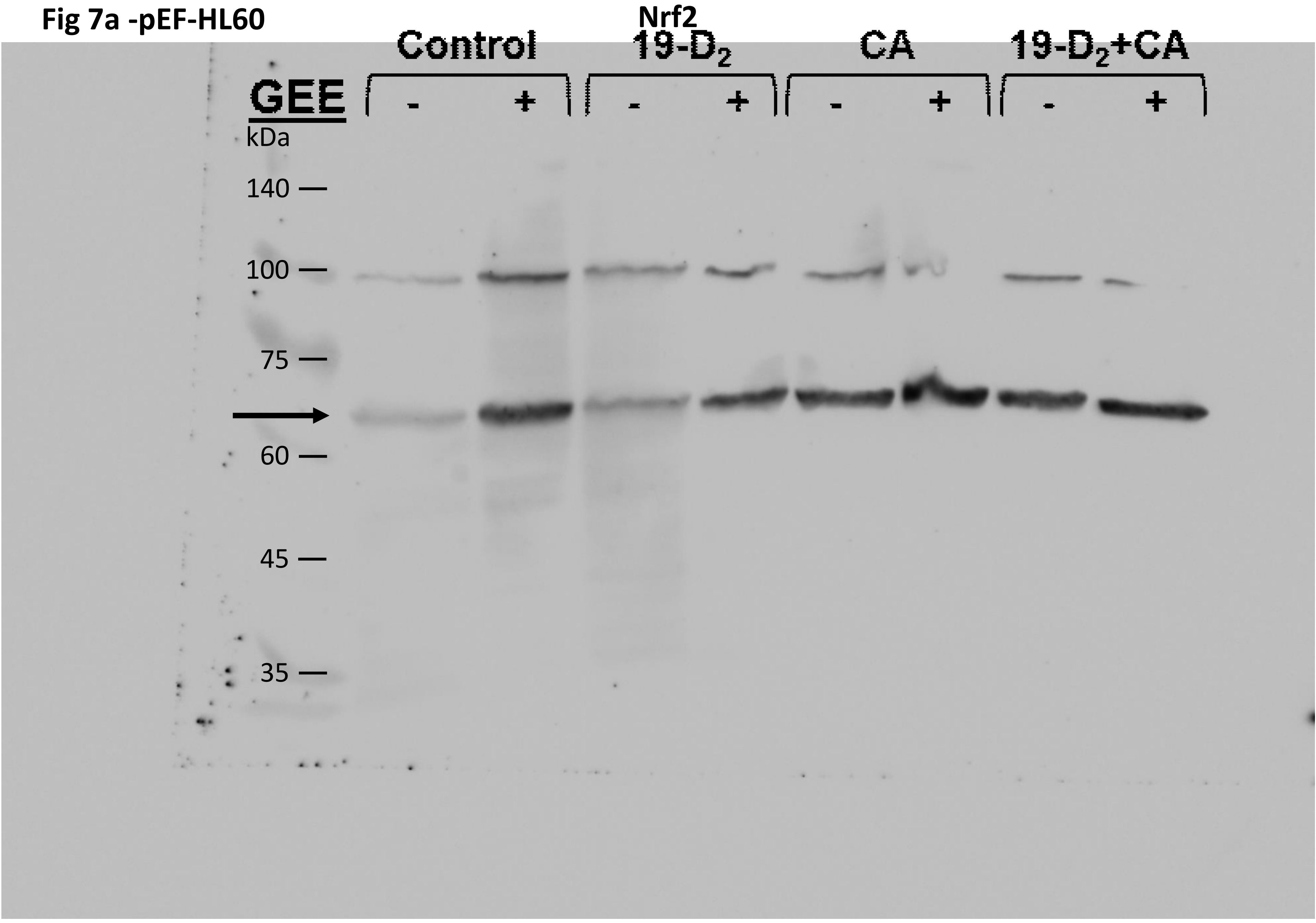

Fig 7a -pEF-HL60

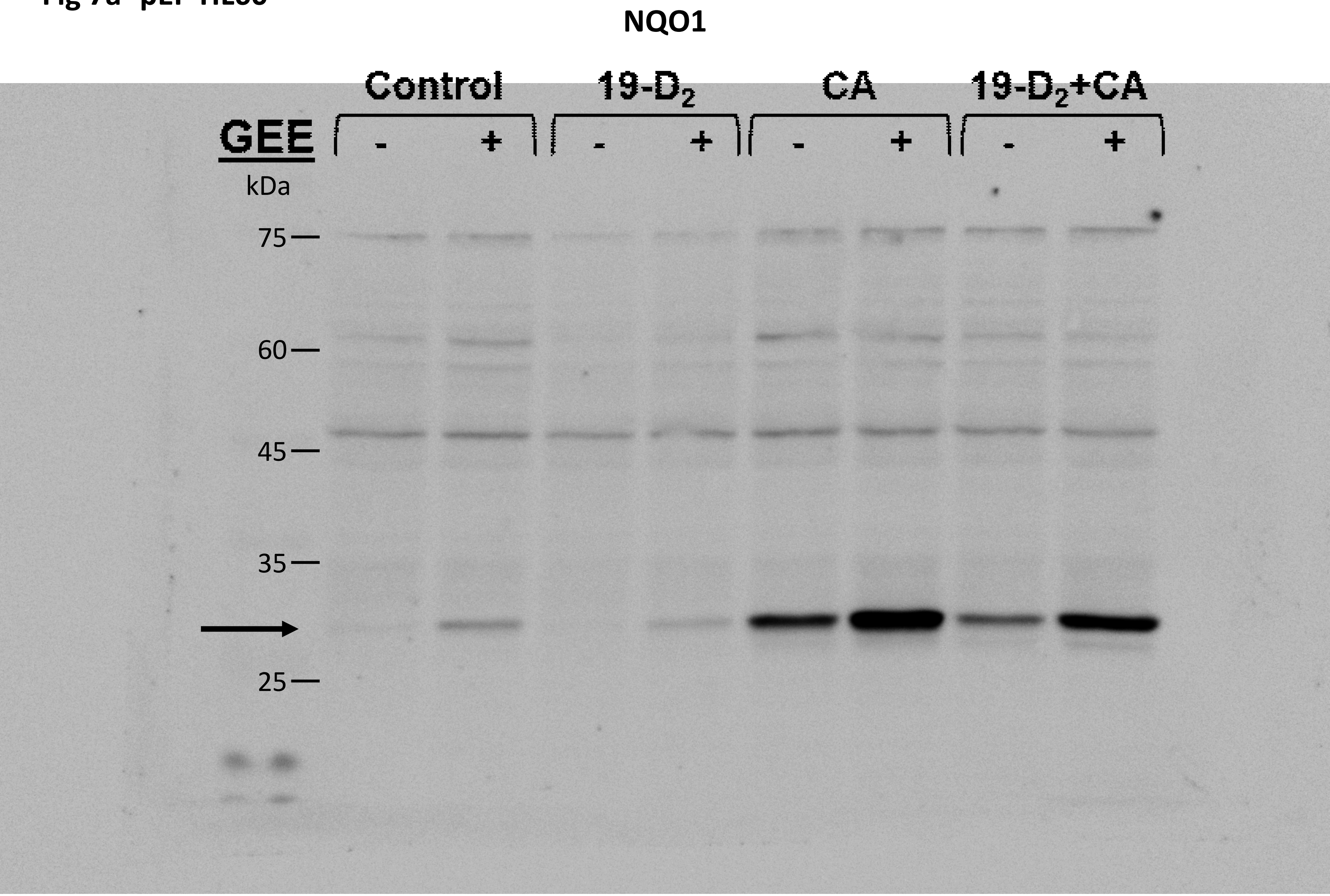

Fig 7a -pEF-HL60

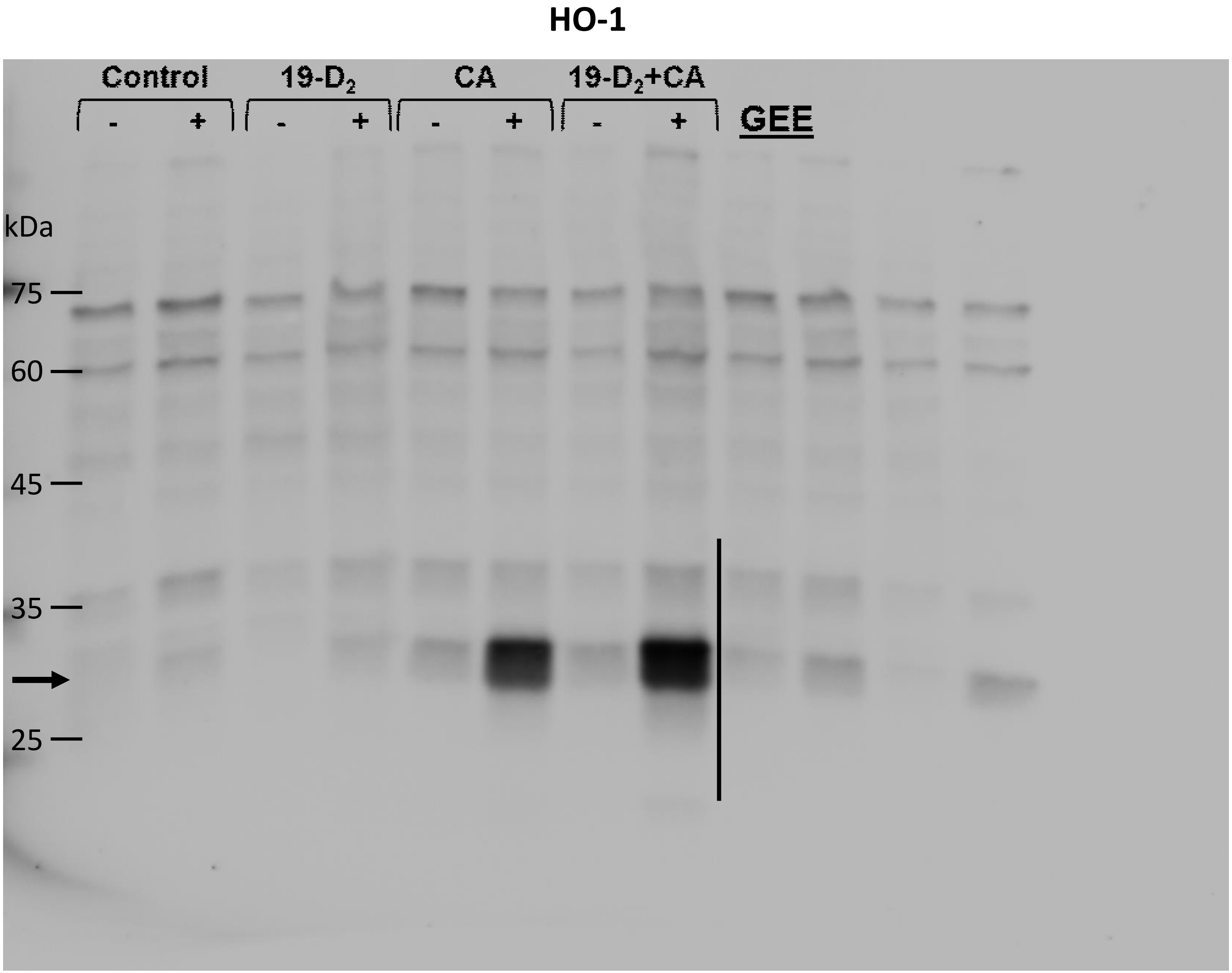

Fig 7a -pEF-HL60

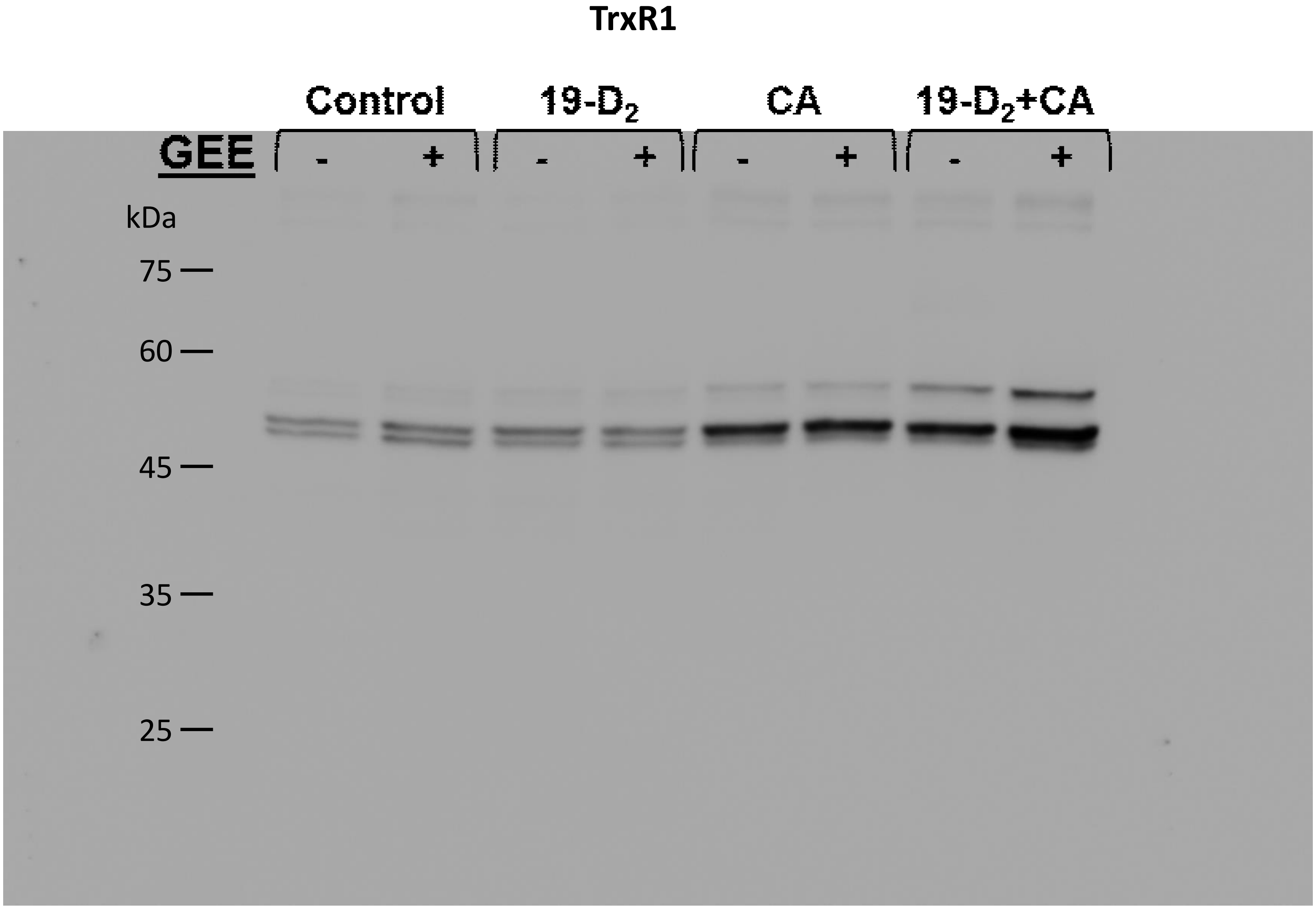

Fig 7a -pEF-HL60 ɣ-GCSc

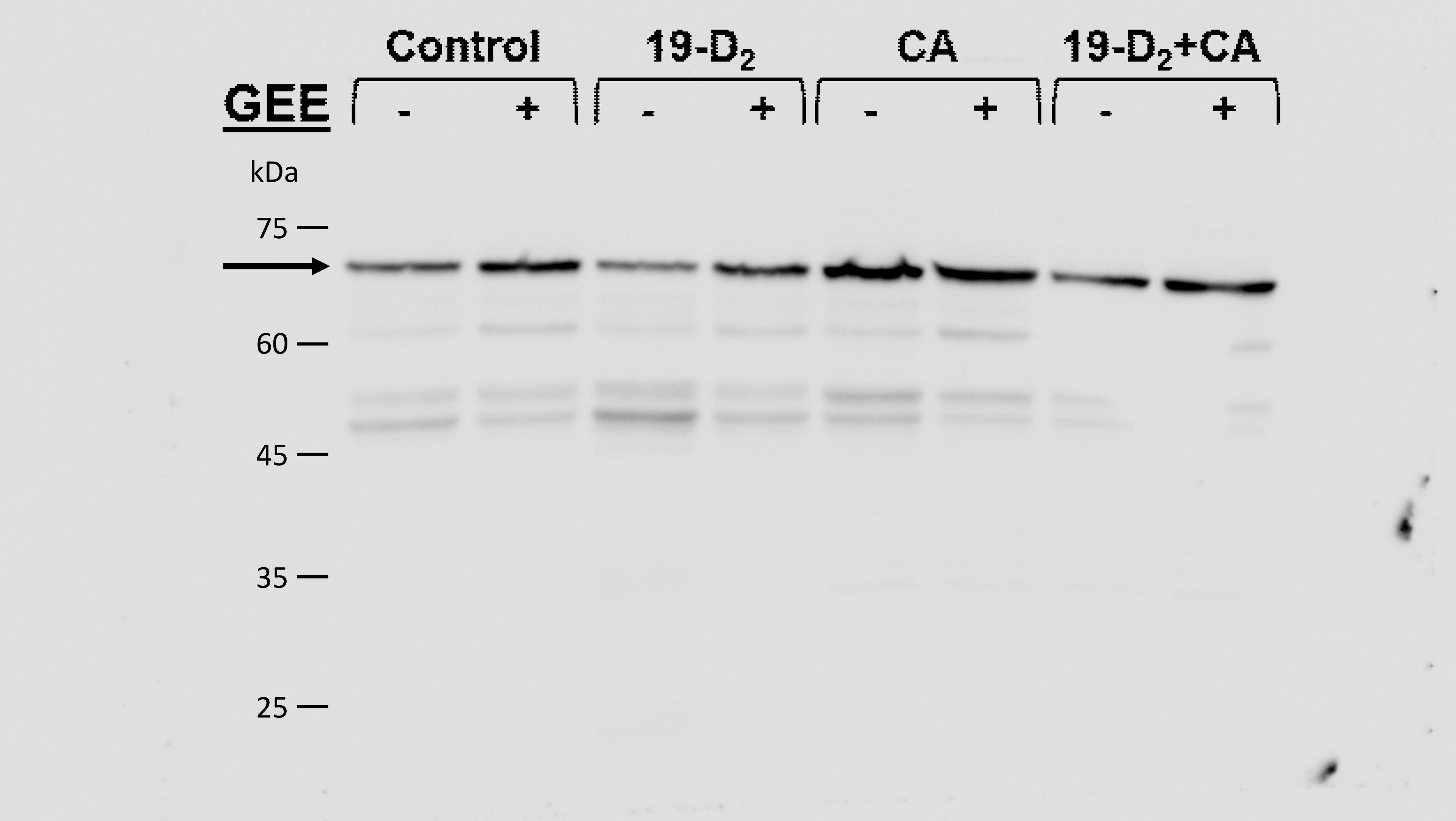

Fig 7a -pEF-HL60 **ɣ-GCSm**

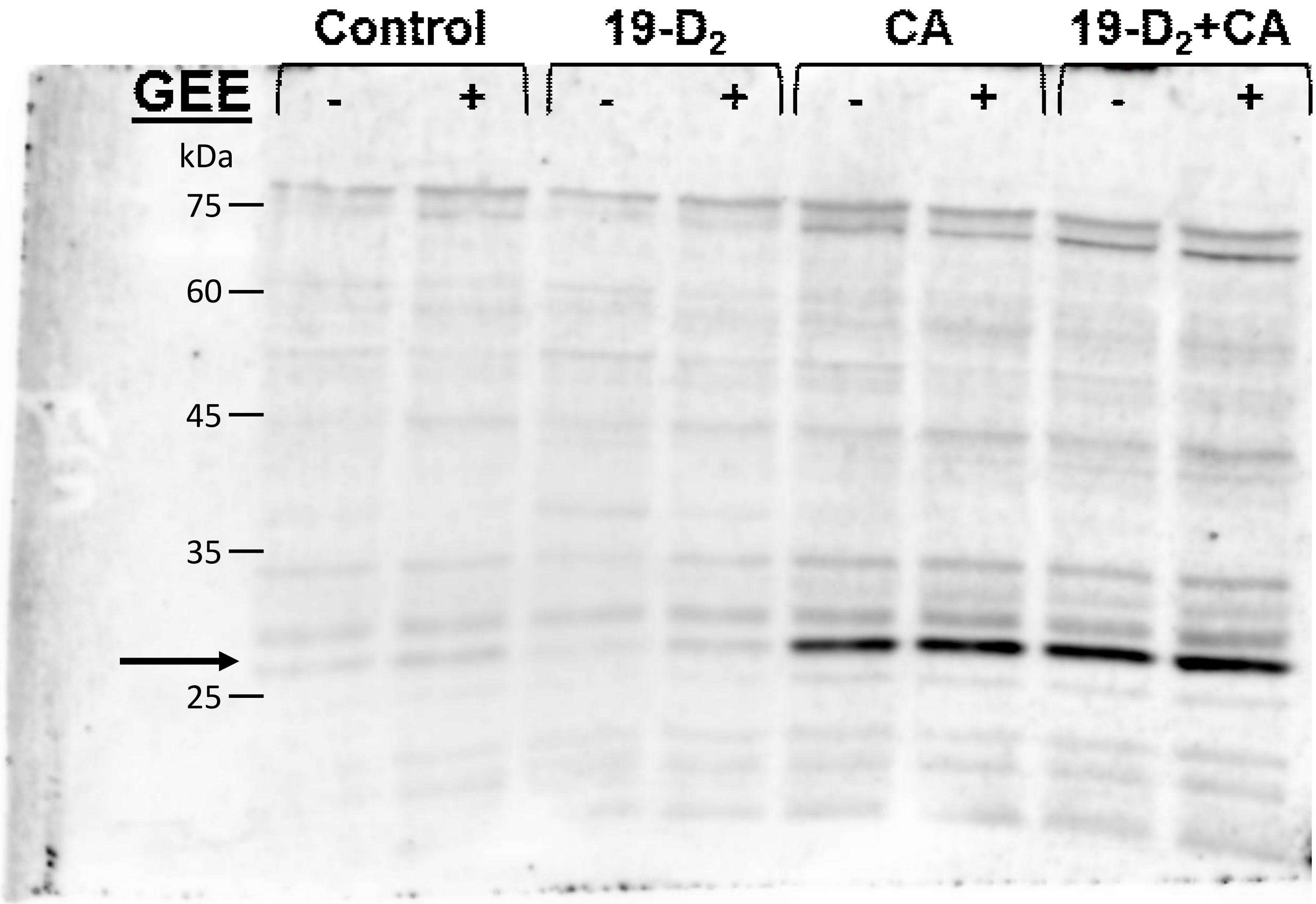

Fig 7a -pEF-HL60

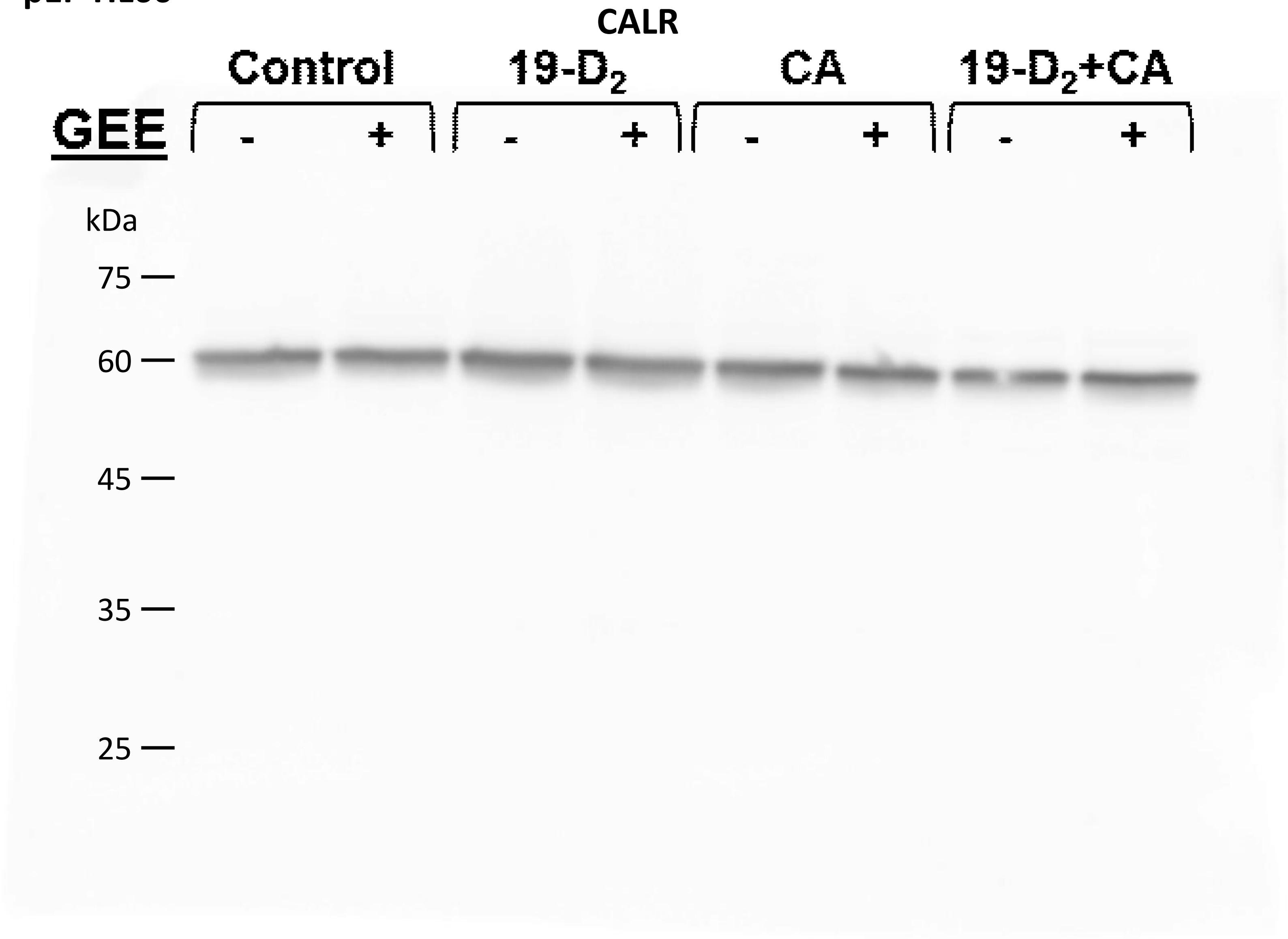

Fig 7a -dnNrf2-HL60 **VDR**

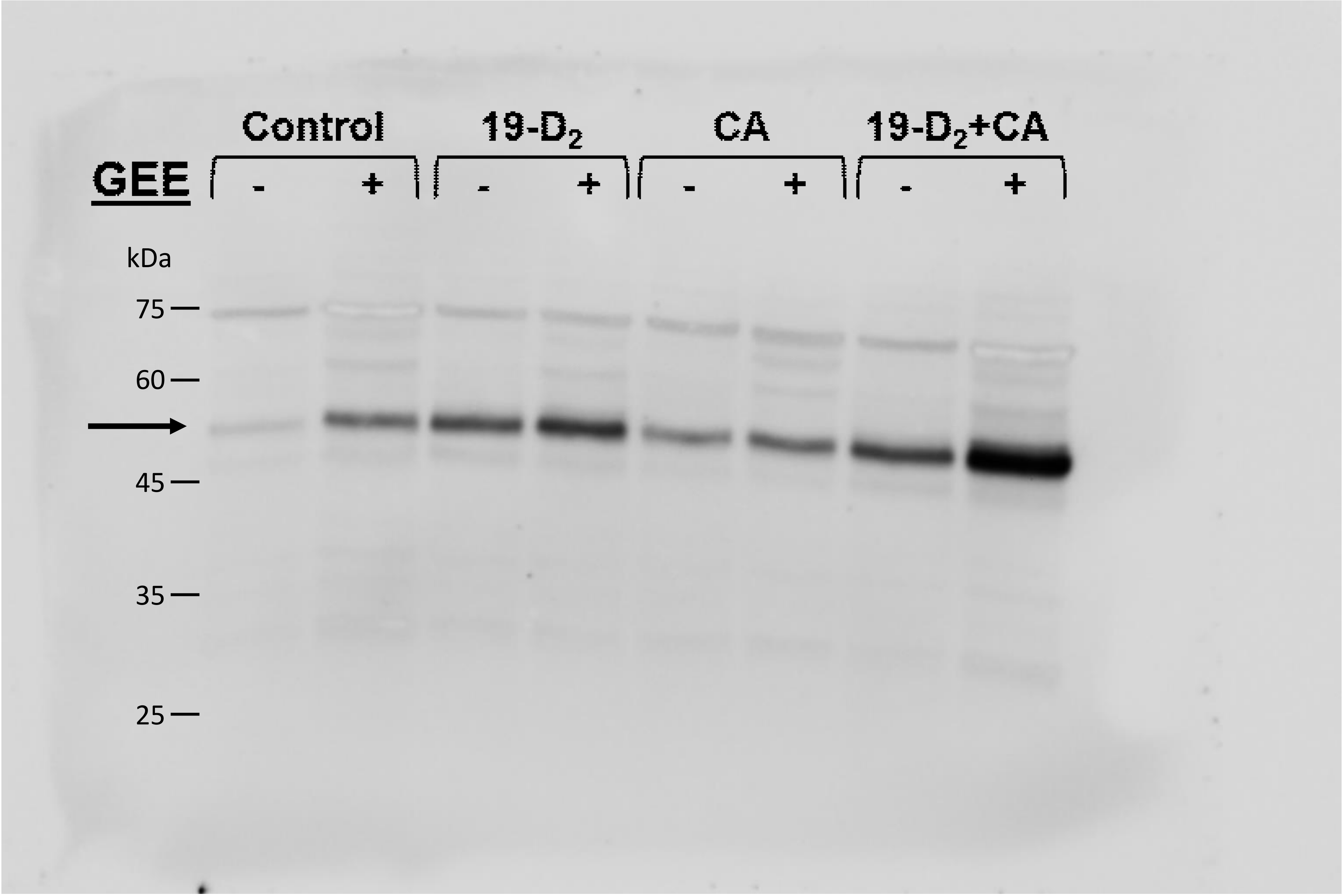

Fig 7a -dnNrf2-HL60

RXRα

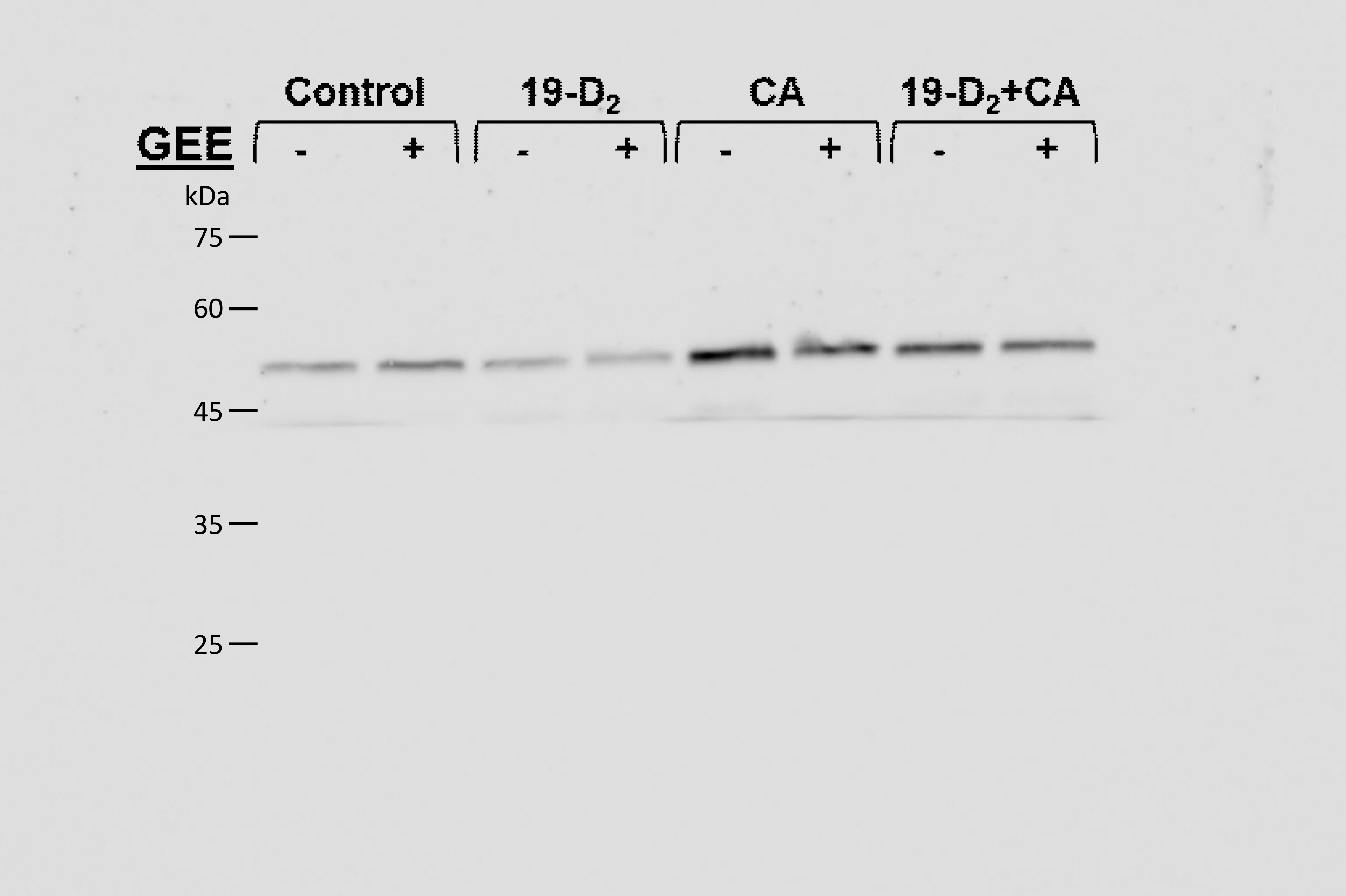

Fig 7a -dnNrf2-HL60

Nrf2

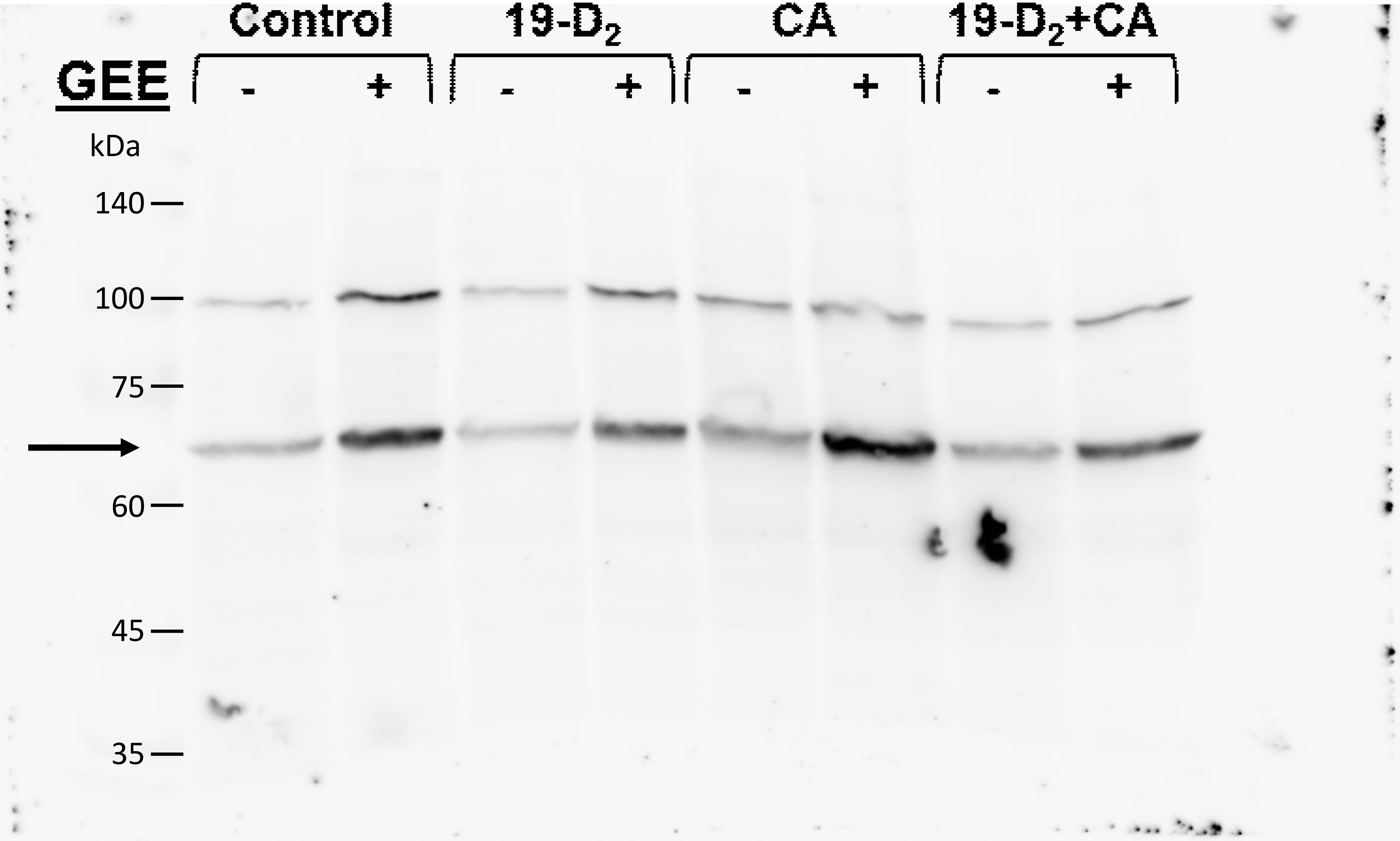

Fig 7a -dnNrf2-HL60

NQO1

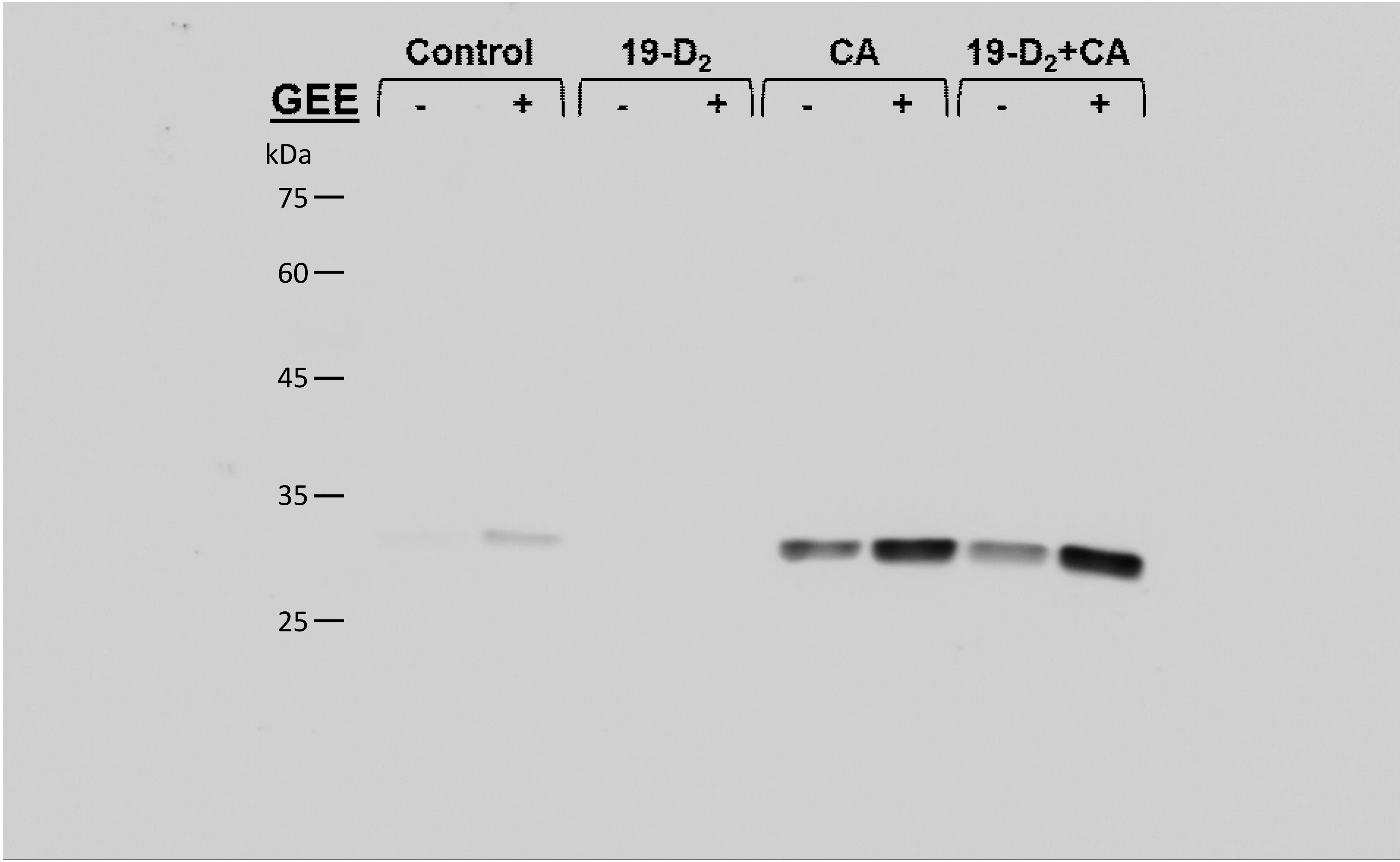

Fig 7a -dnNrf2-HL60 HO-1

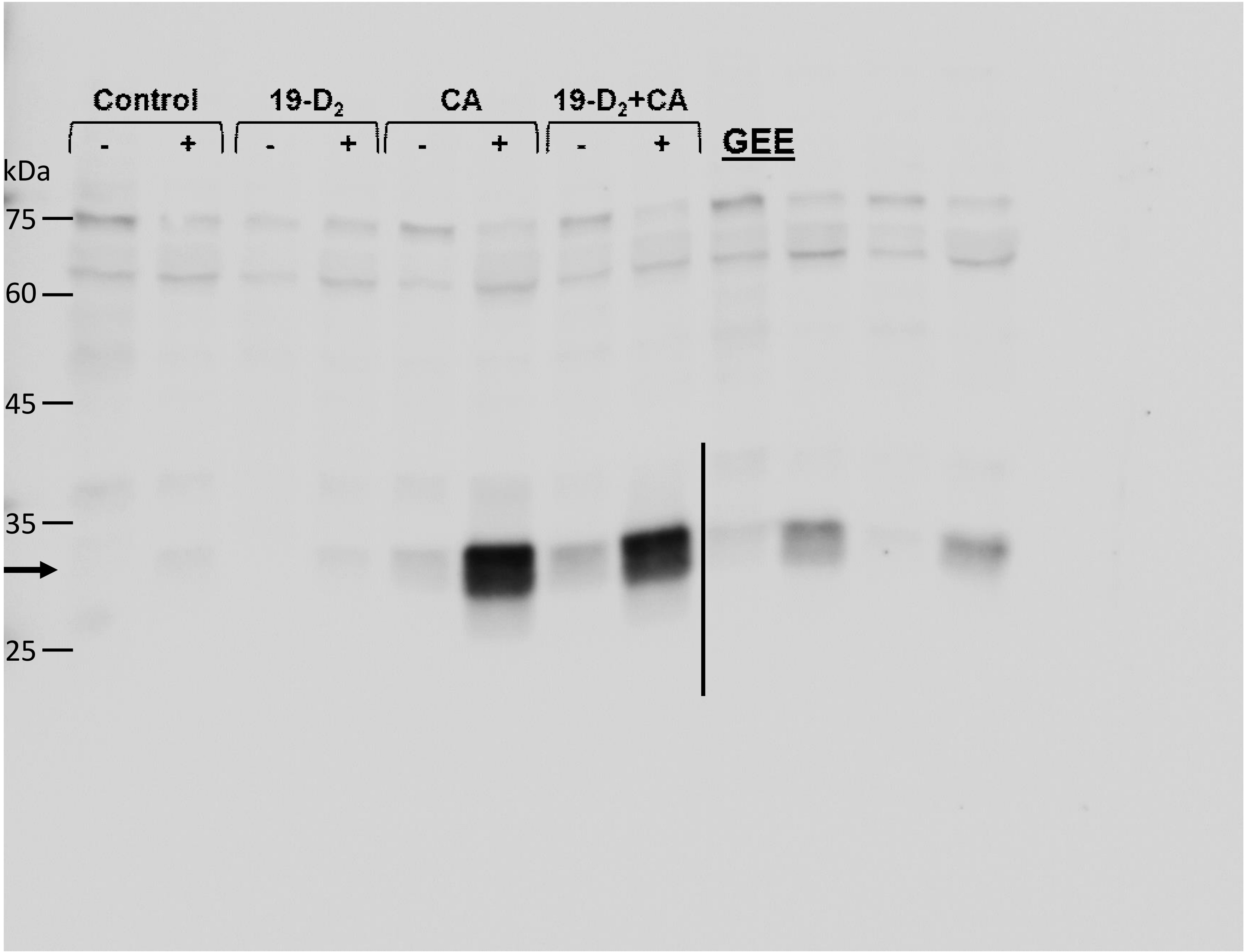

Fig 7a -dnNrf2-HL60

TrxR1

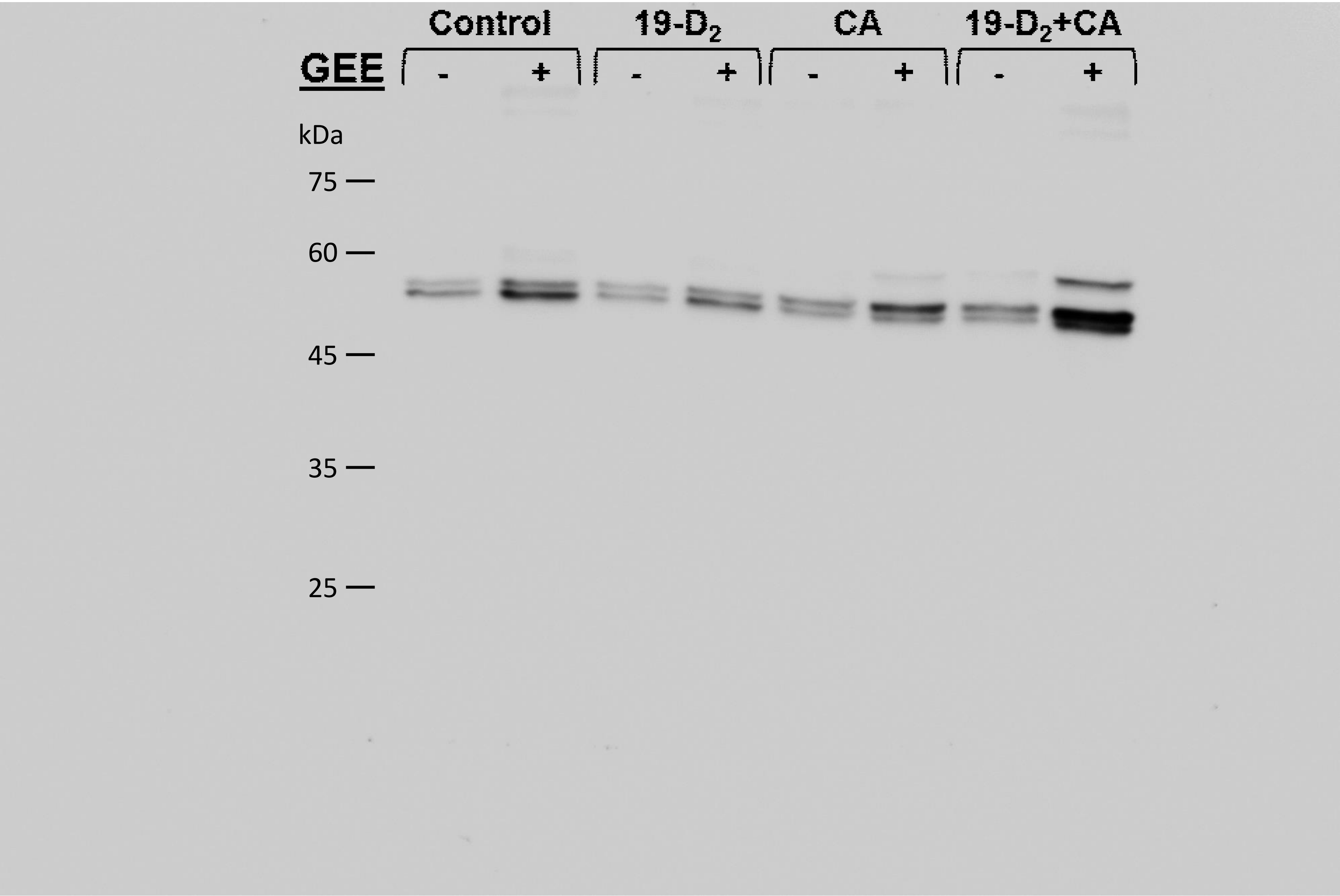

Fig 7a -dnNrf2-HL60

γ-GCSc

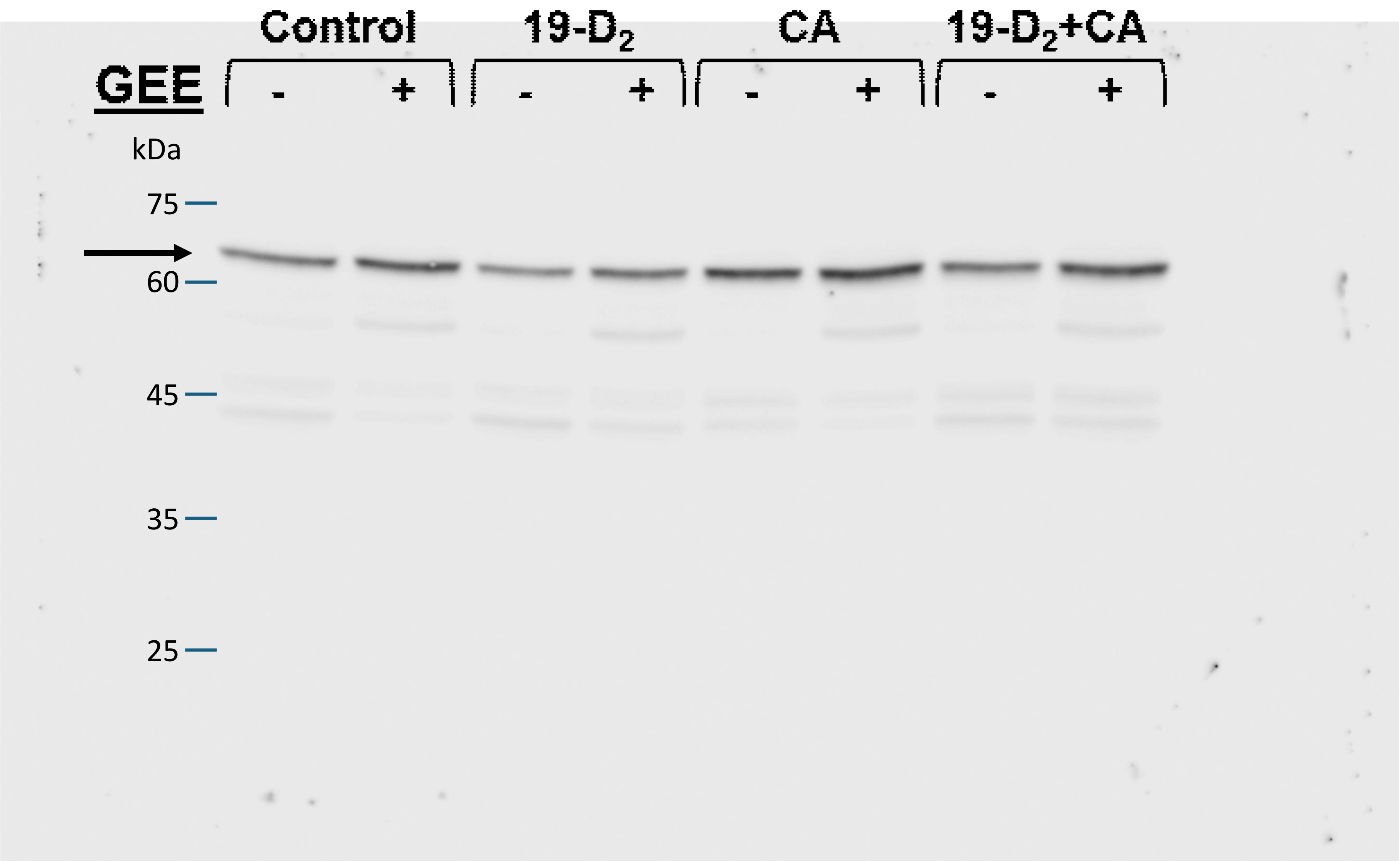

Fig 7a -dnNrf2-HL60

γ-GCSm

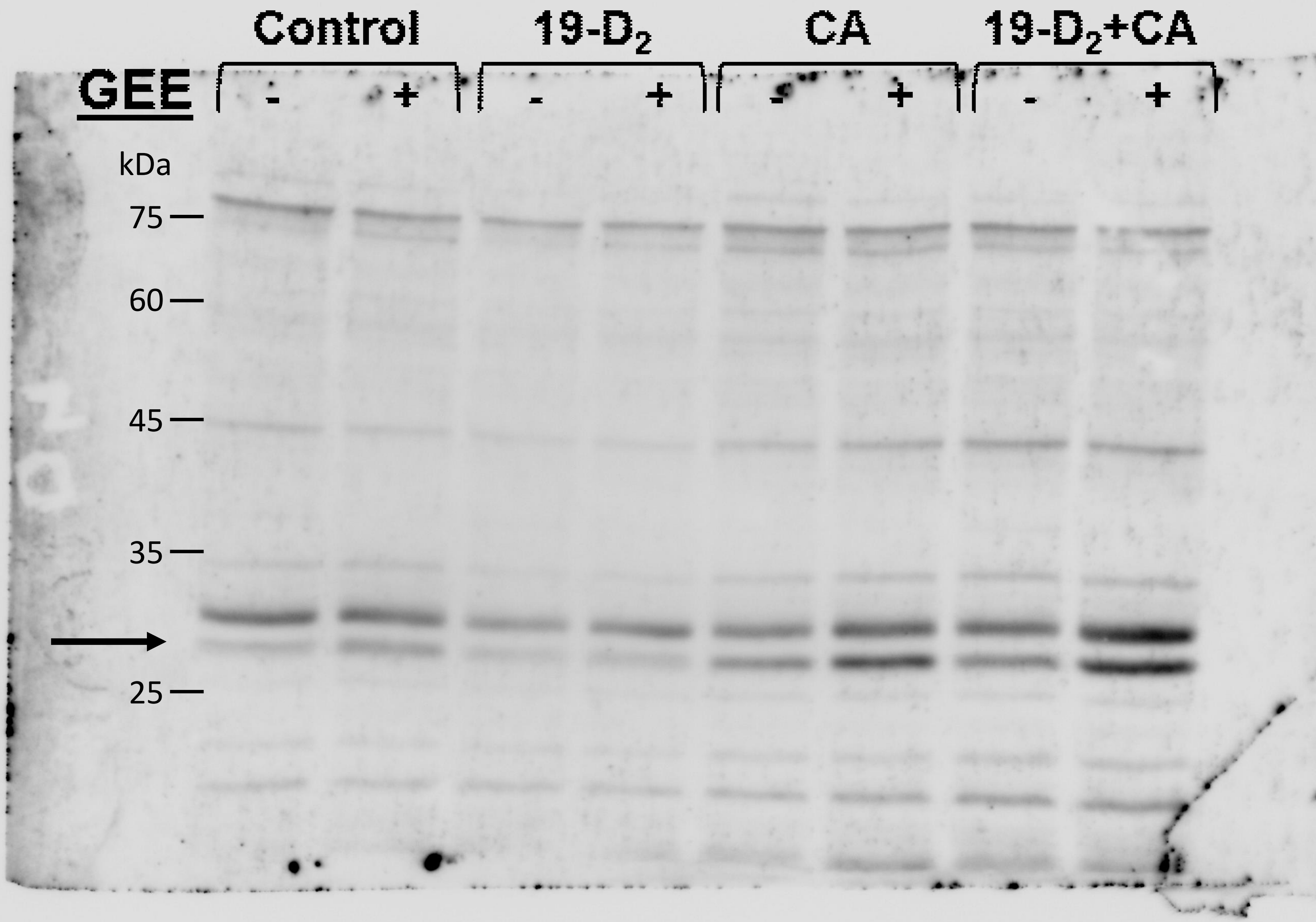

Fig 7a -dnNrf2-HL60 CALR

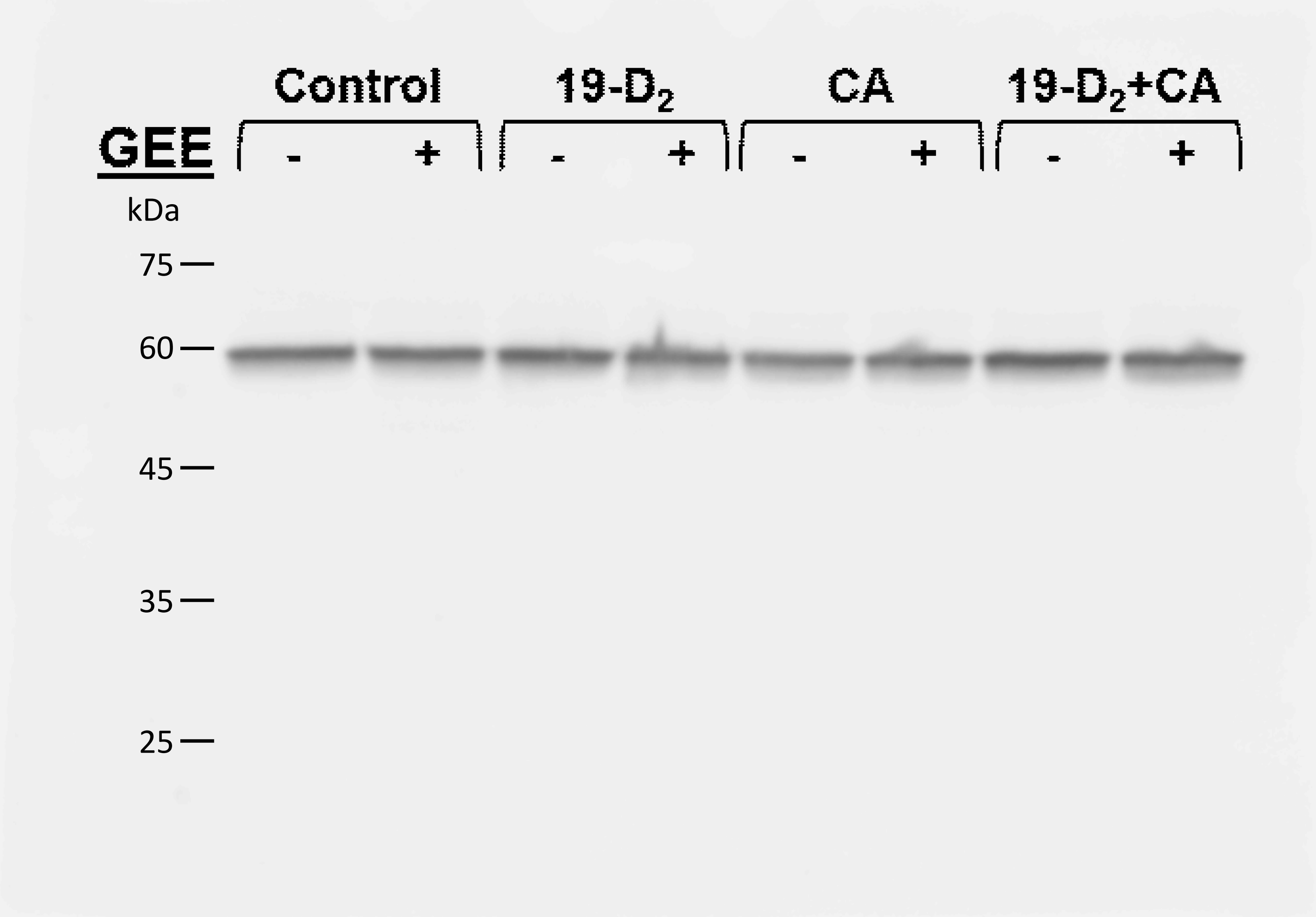

Fig 7a -pEF-HL60

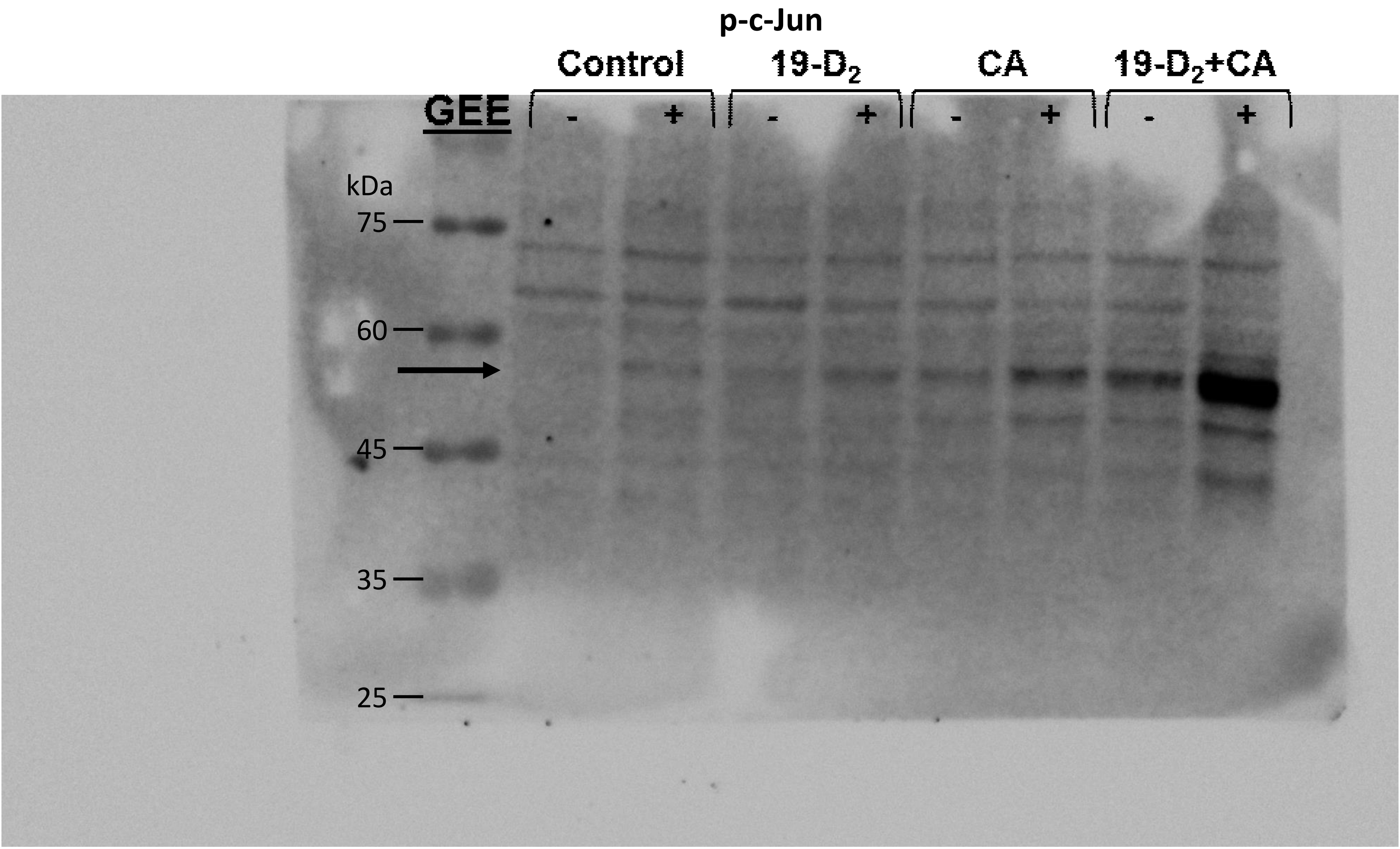

Fig 7j -pEF-HL60

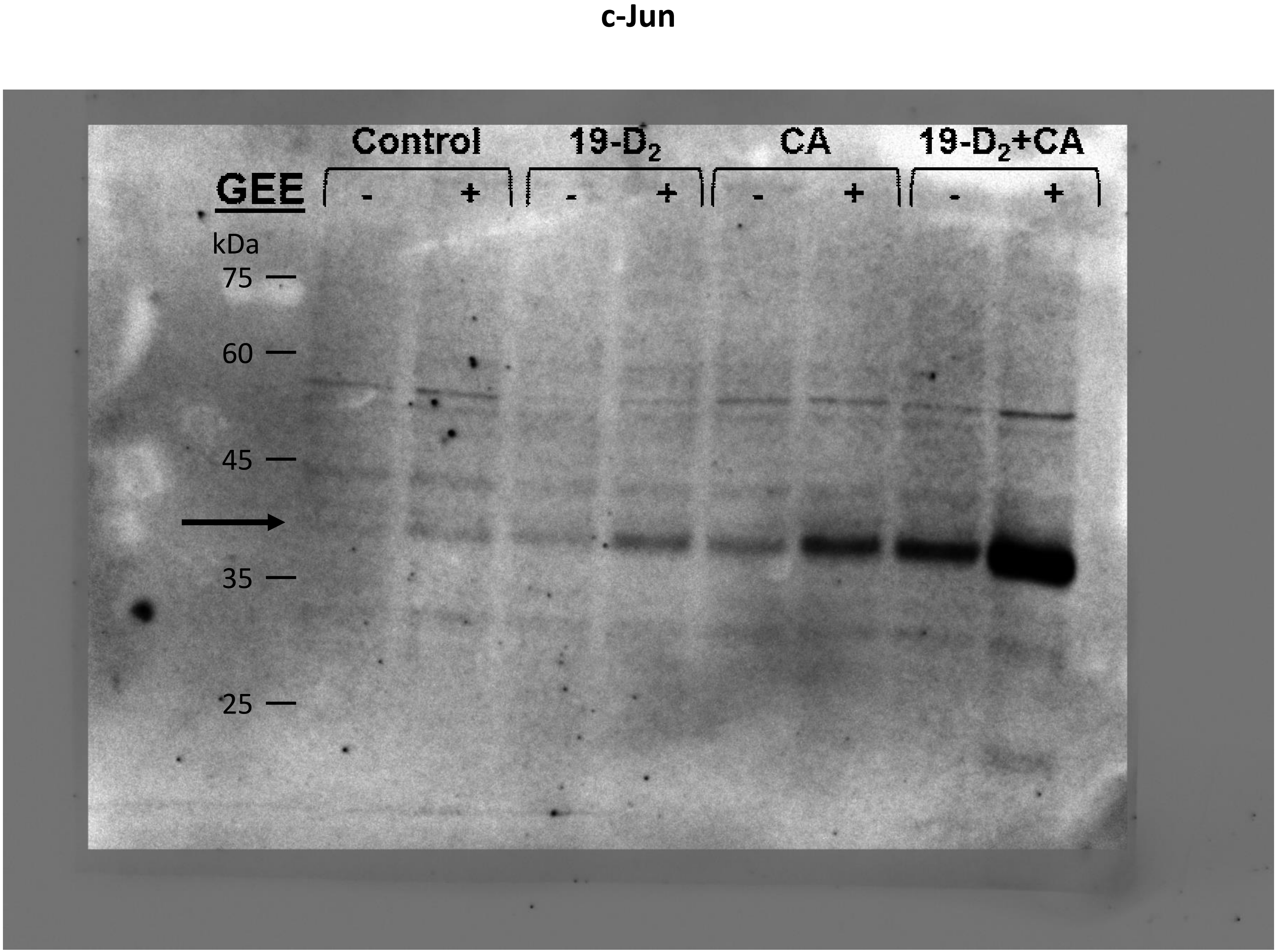

Fig 7a - pEF-HL60

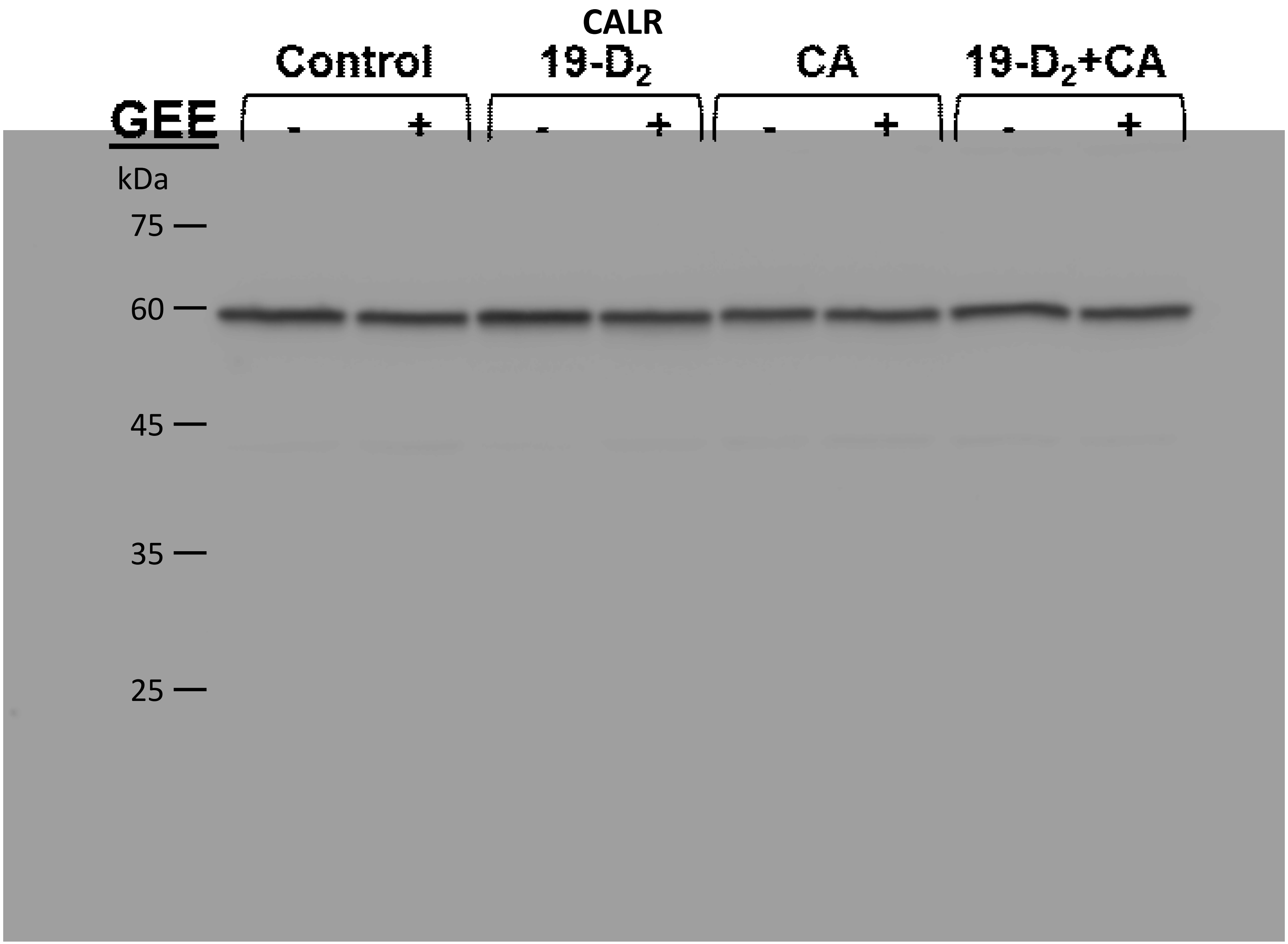

Fig 7j -dnNrf2-HL60

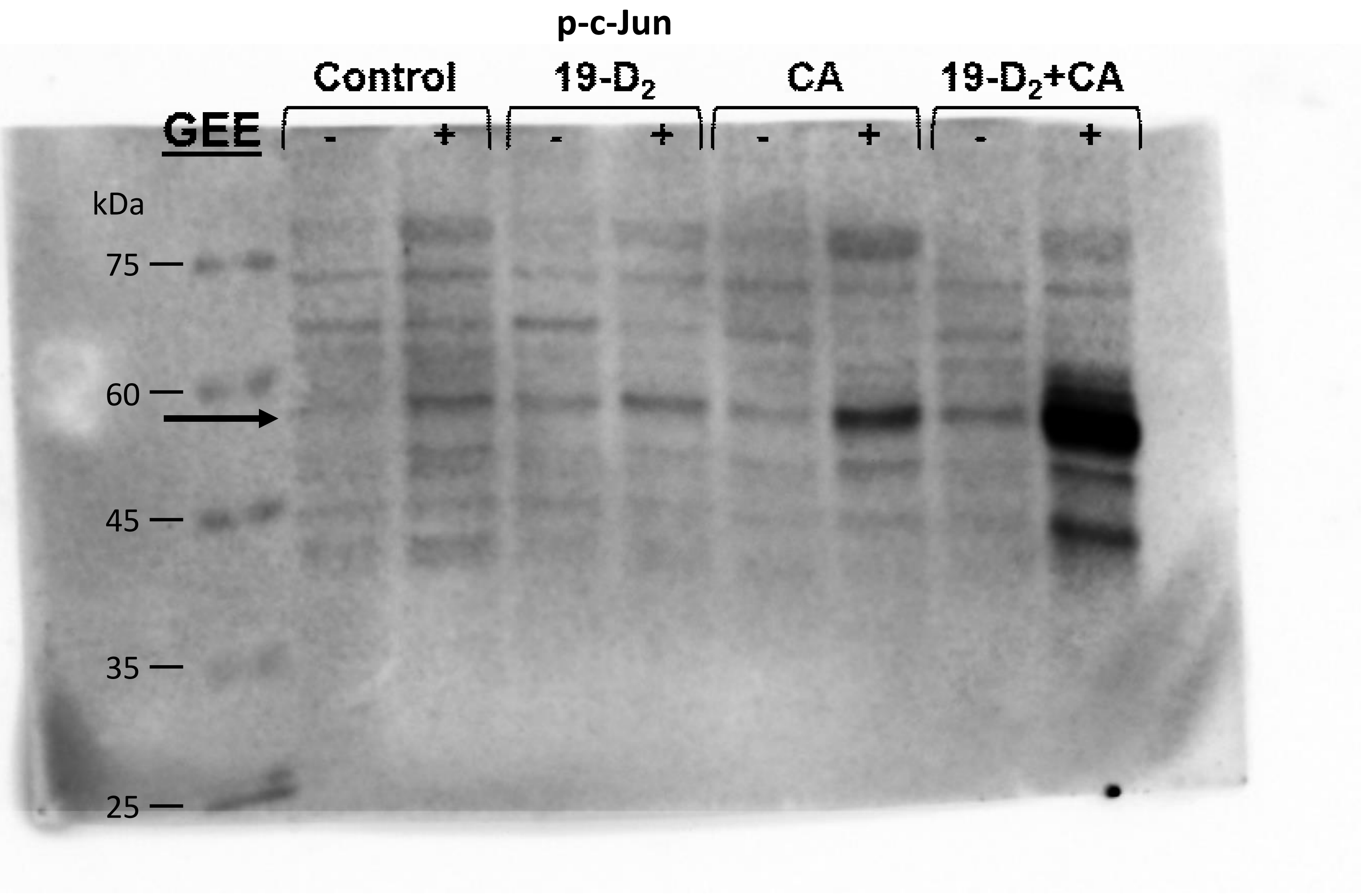

Fig 7j -dnNrf2-HL60

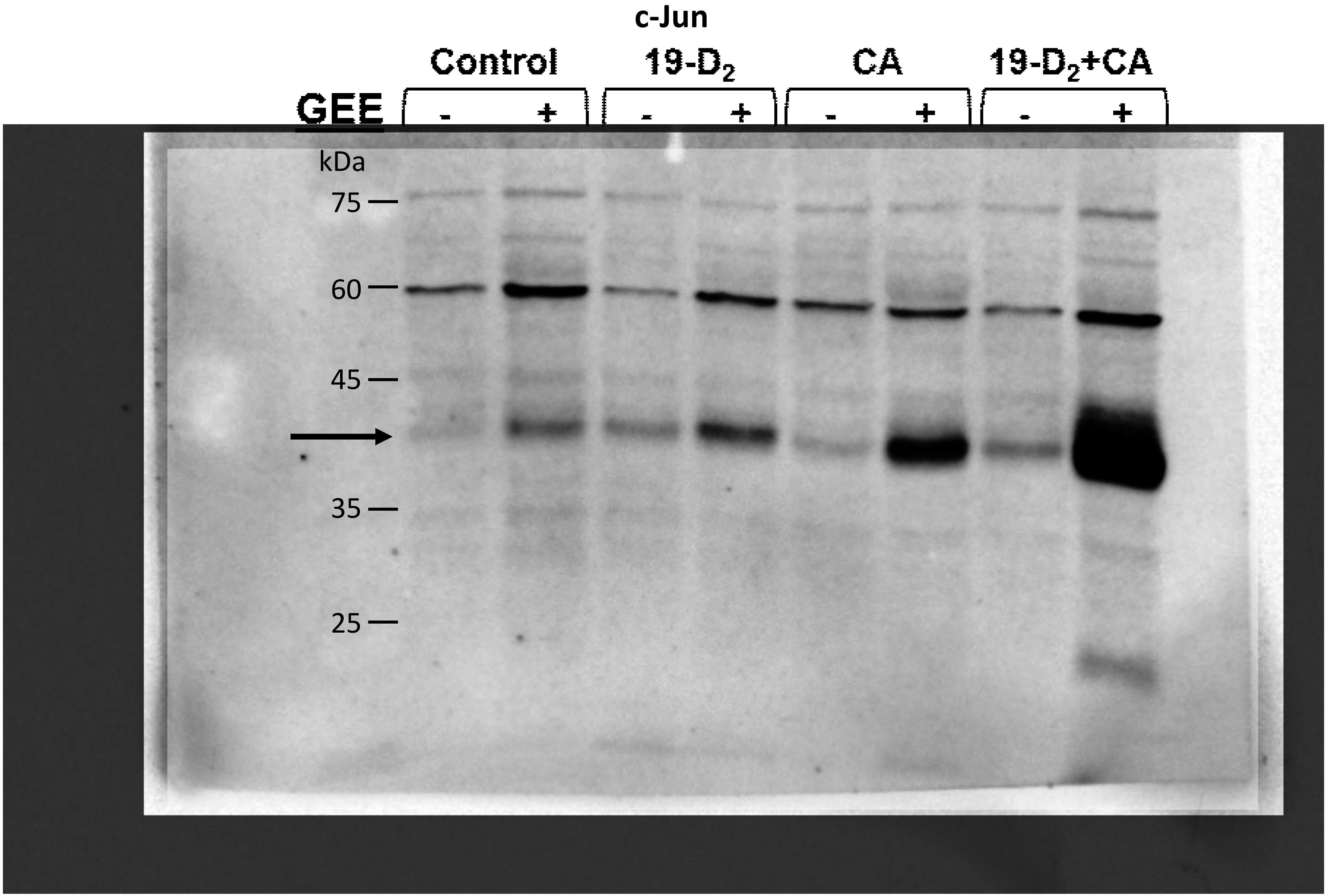

Fig 7j -dnNrf2-HL60

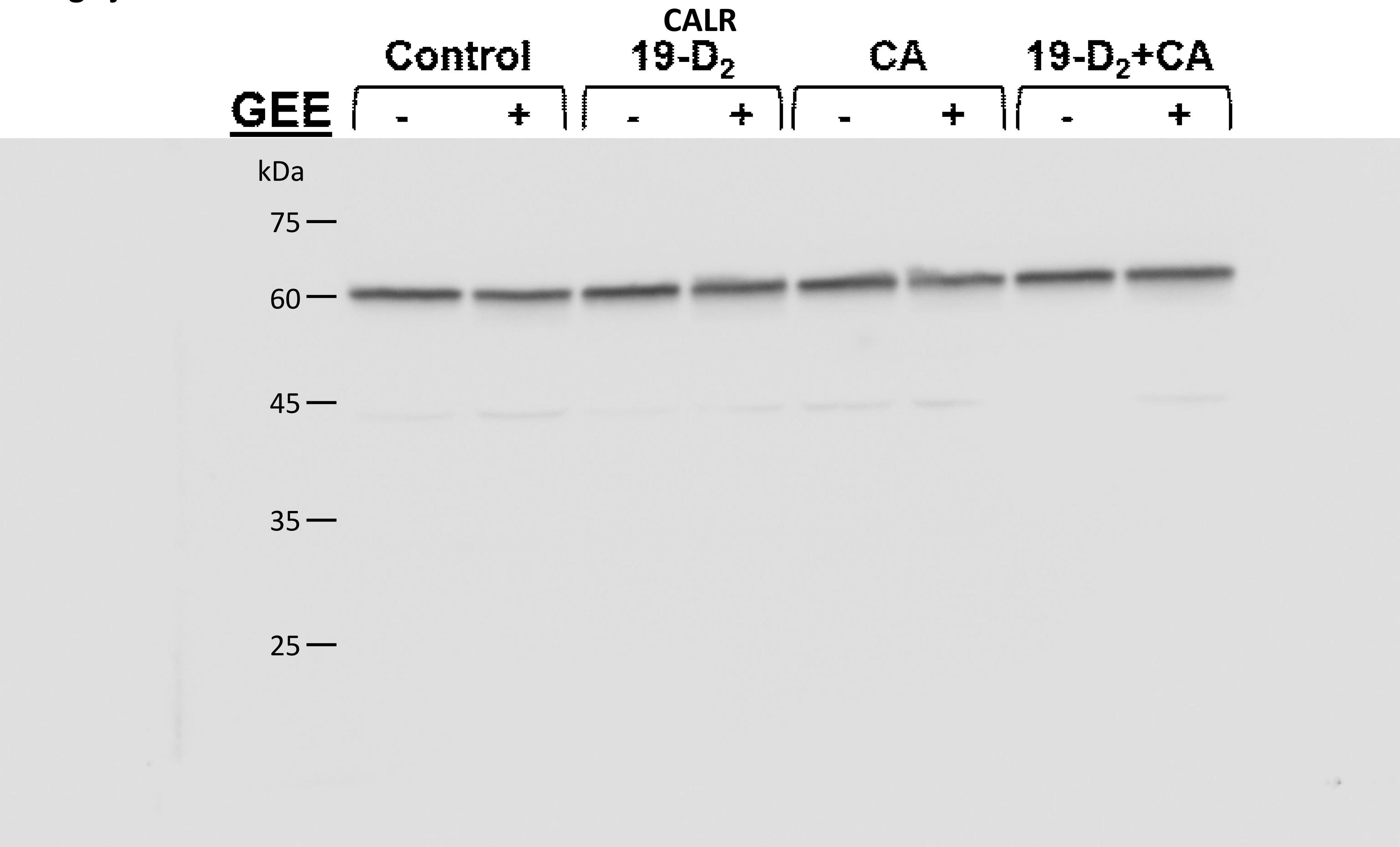

Fig 8b

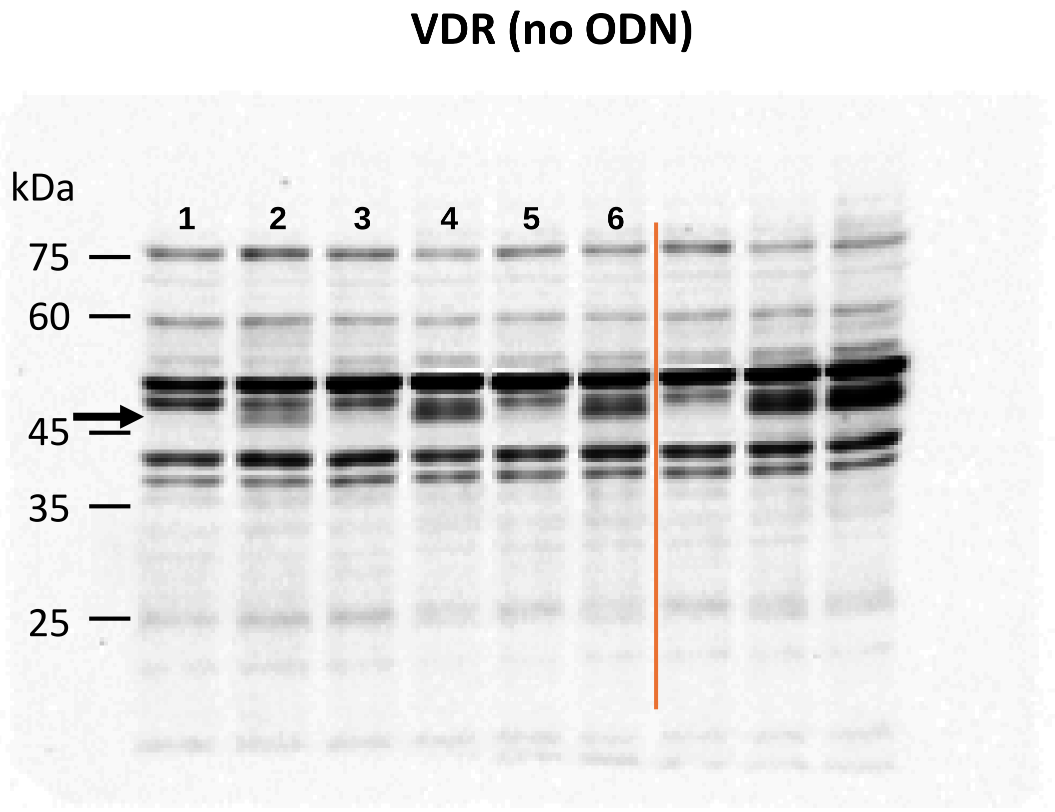

1- Control
2- 1,25D_3
3- CA
4- 1,25D_3+CA
5- MMF
6- 1,25D_3+MMF

Fig 8b

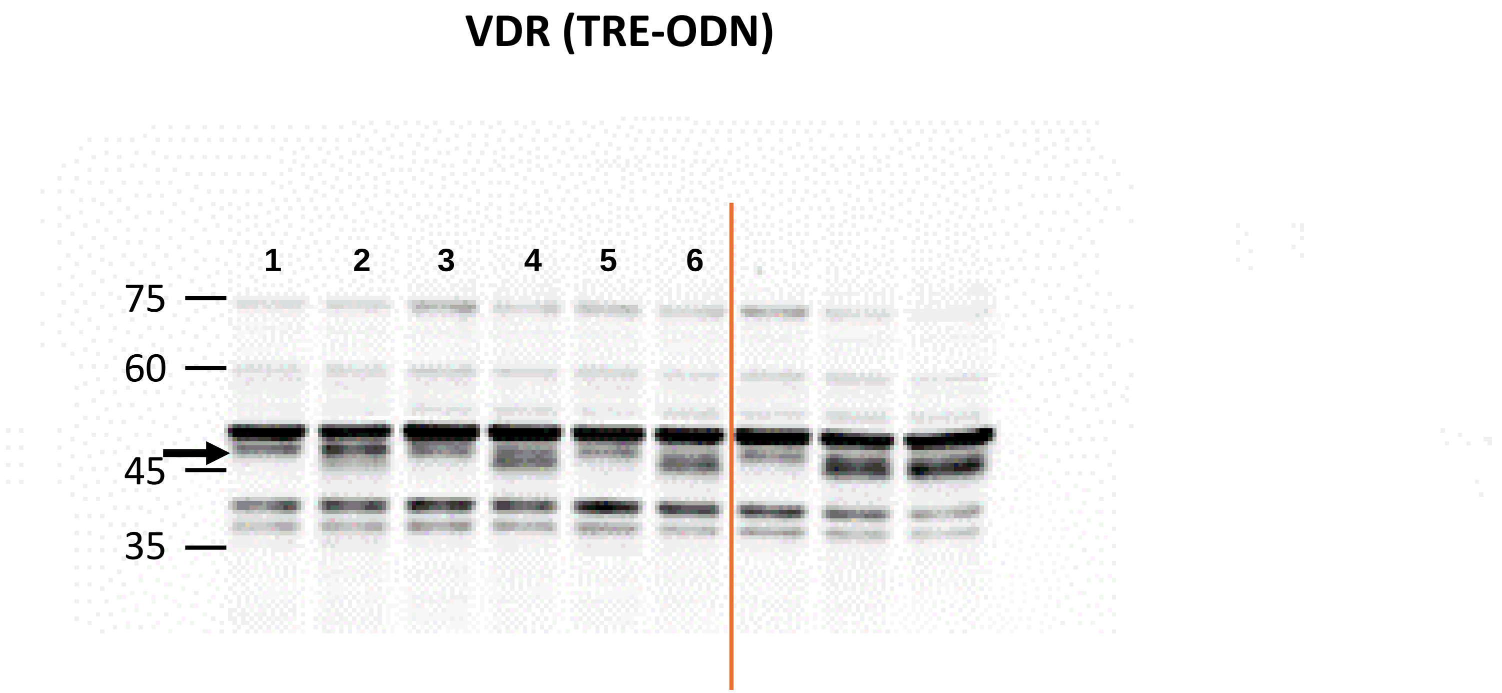

1- Control
2- 1,25D$_3$
3- CA
4- 1,25D$_3$+CA
5- MMF
6- 1,25D$_3$+MMF

Fig 8b

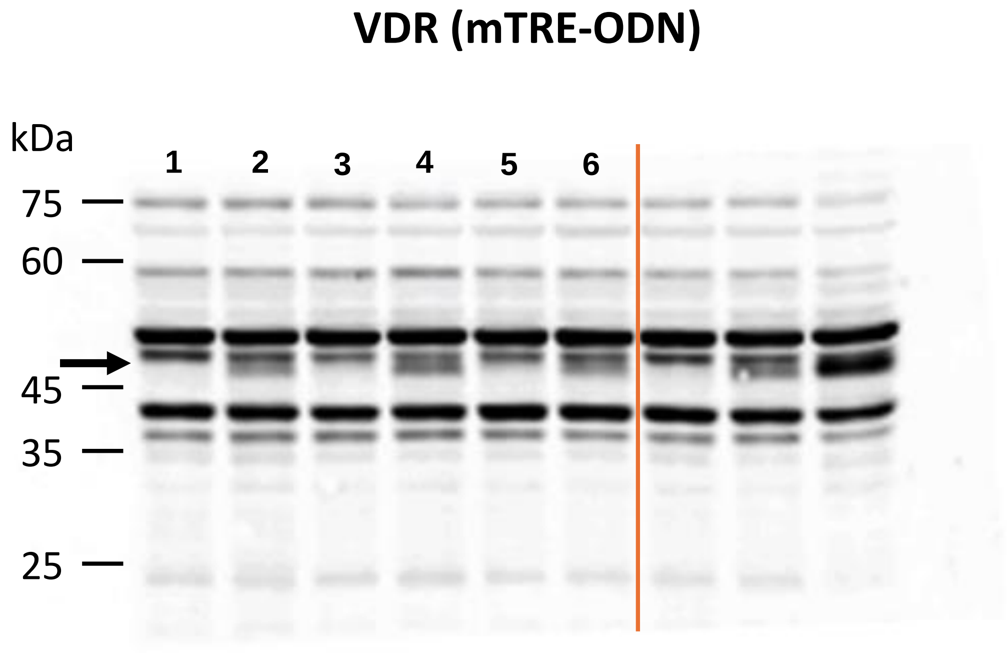

1- Control
2- 1,25D_3
3- CA
4- 1,25D_3+CA
5- MMF
6- 1,25D_3+MMF

Fig 8b

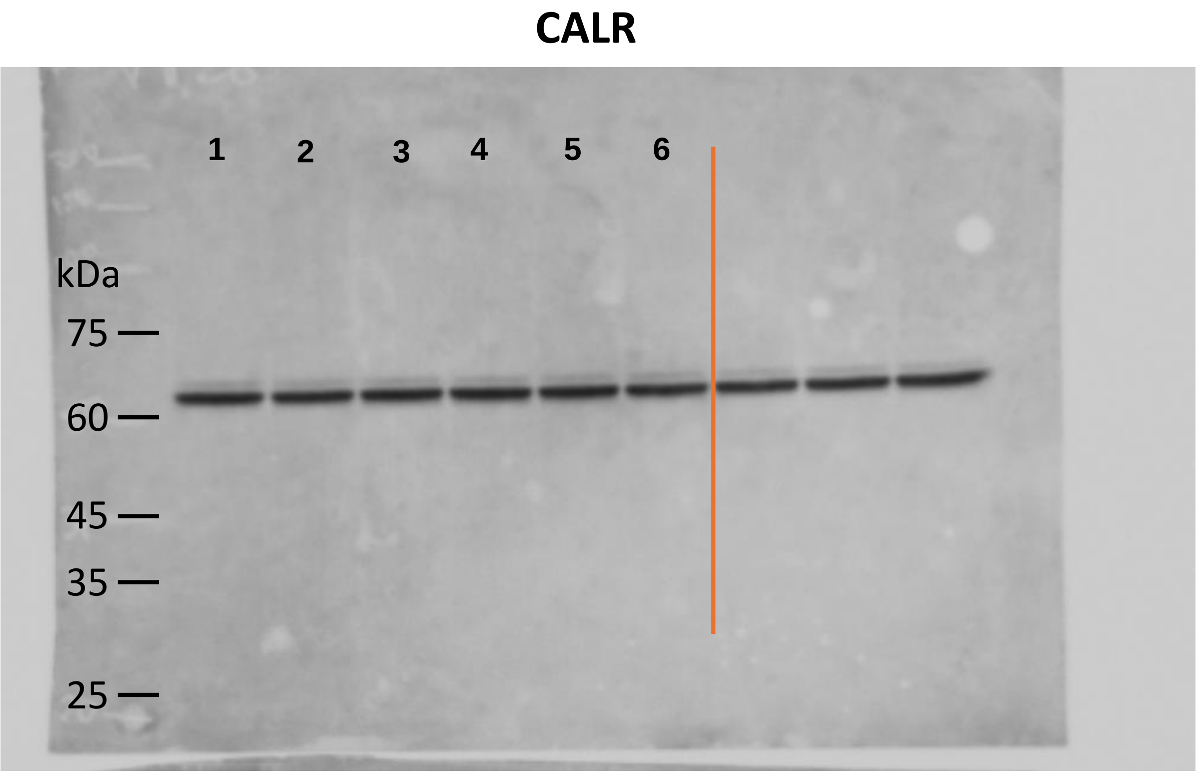

1- Control
2- $1,25D_3$
3- CA
4- $1,25D_3$+CA
5- MMF
6- $1,25D_3$+MMF

Fig 8c

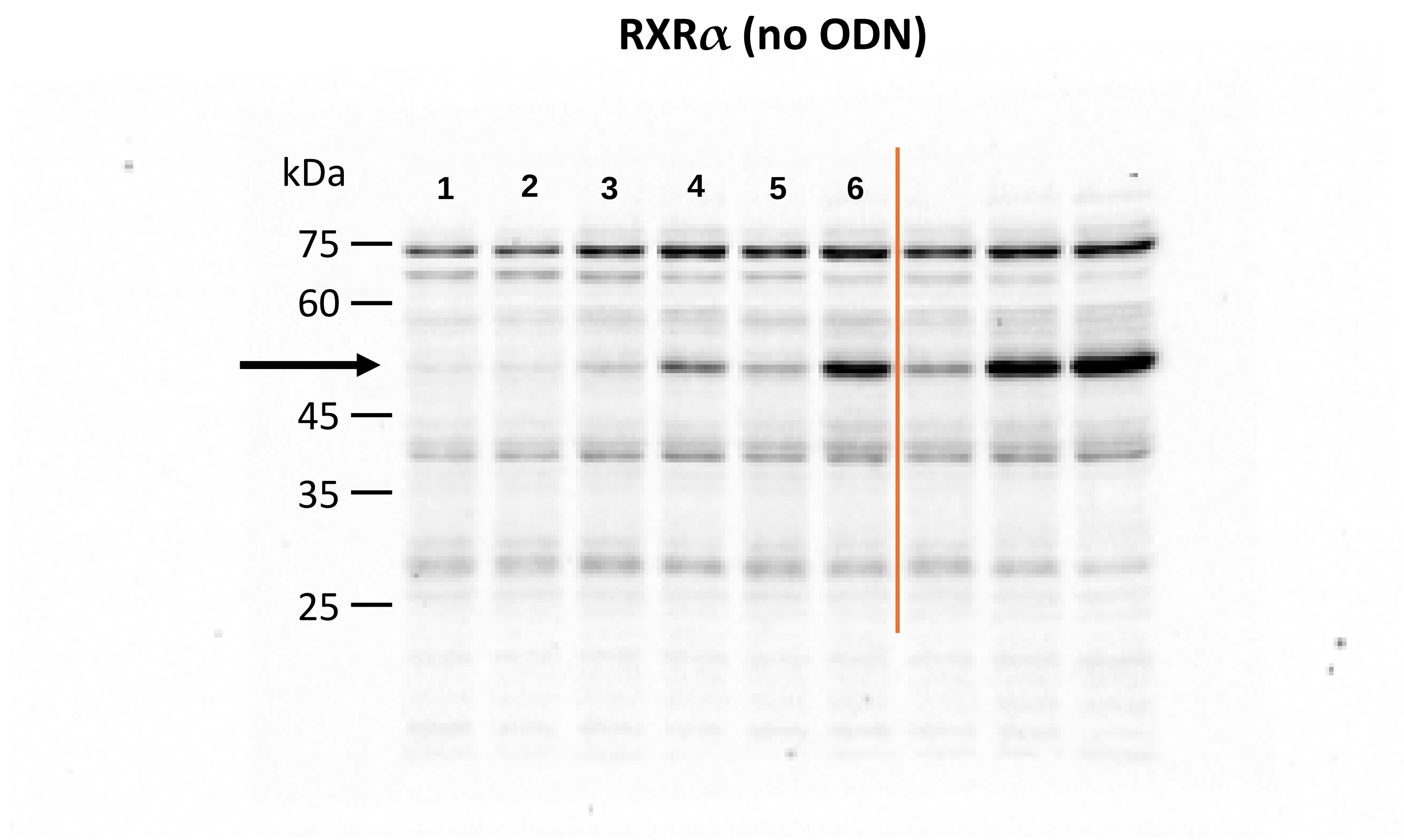

1- Control
2- $1,25D_3$
3- CA
4- $1,25D_3$+CA
5- MMF
6- $1,25D_3$+MMF

Fig 8c

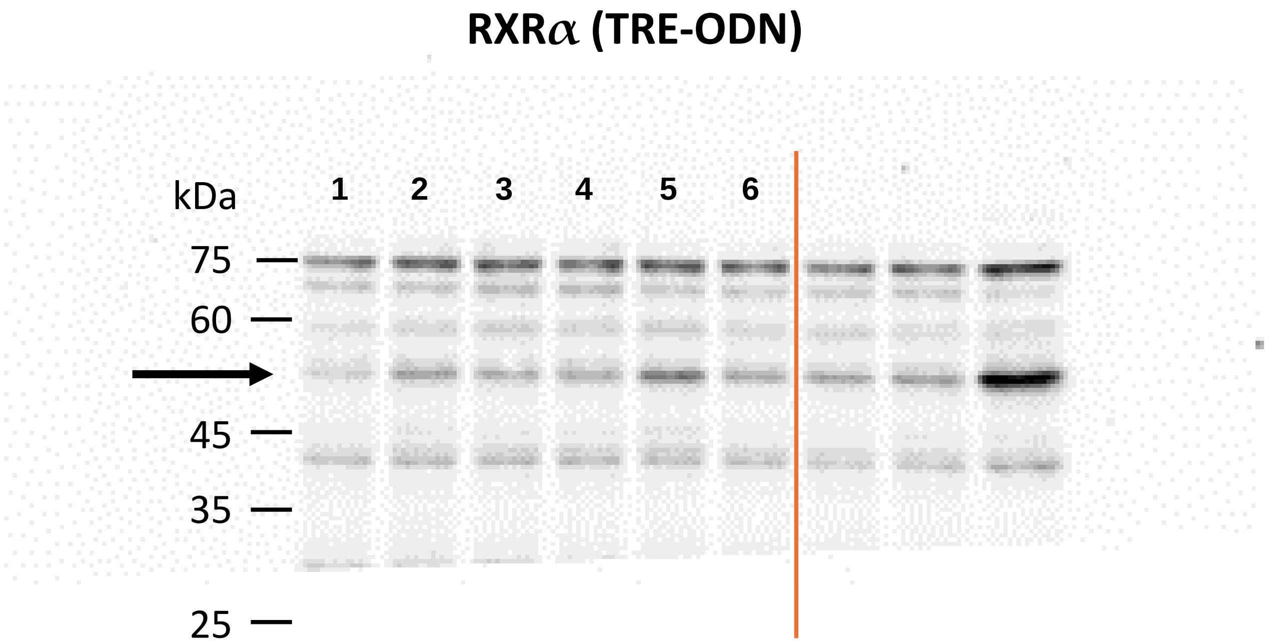

1- Control
2- 1,25D_3
3- CA
4- 1,25D_3+CA
5- MMF
6- 1,25D_3+MMF

Fig 8c

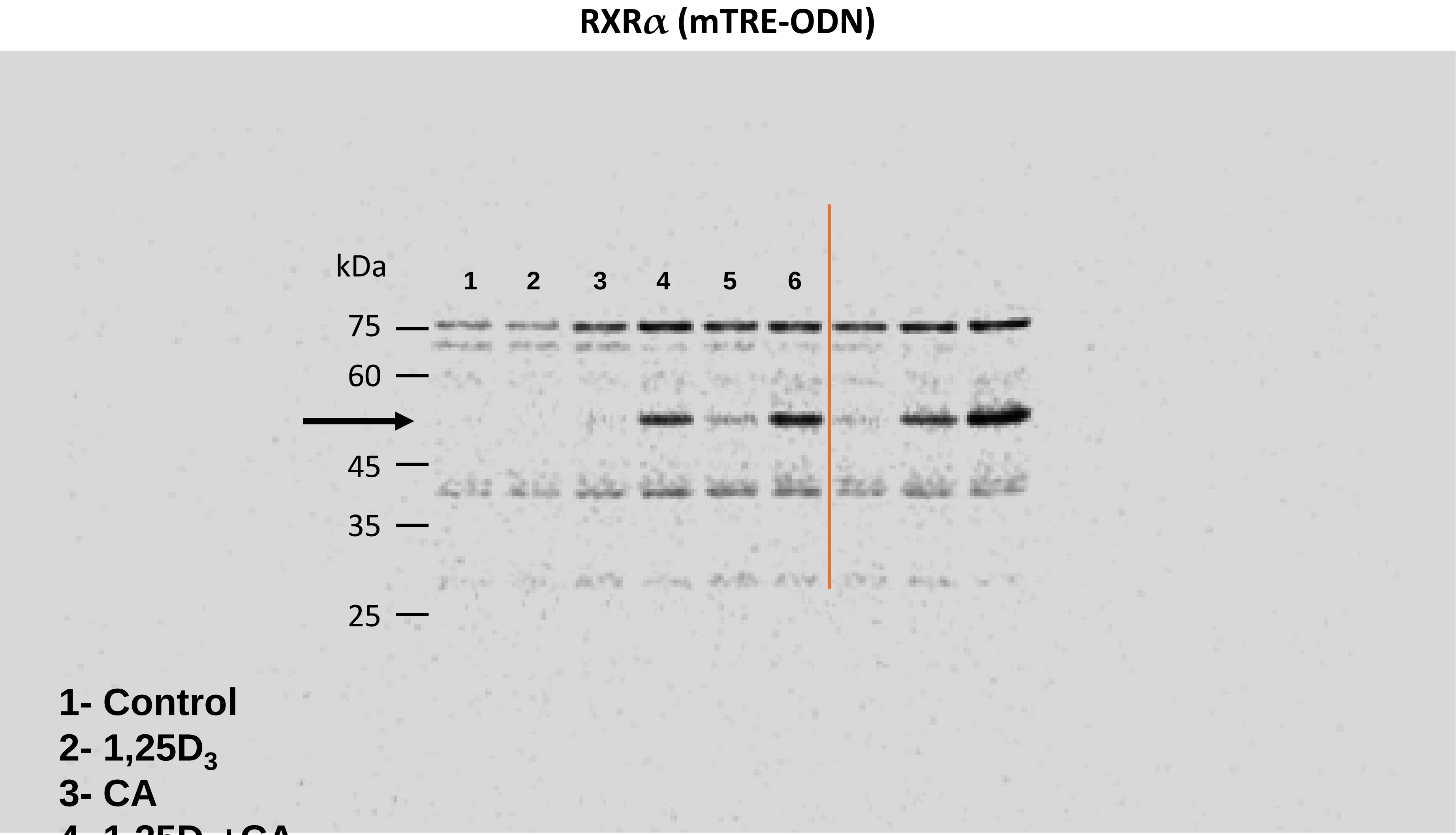

1- Control
2- 1,25D_3
3- CA
4- 1,25D_3+CA
5- MMF
6- 1,25D_3+MMF

Fig 8c

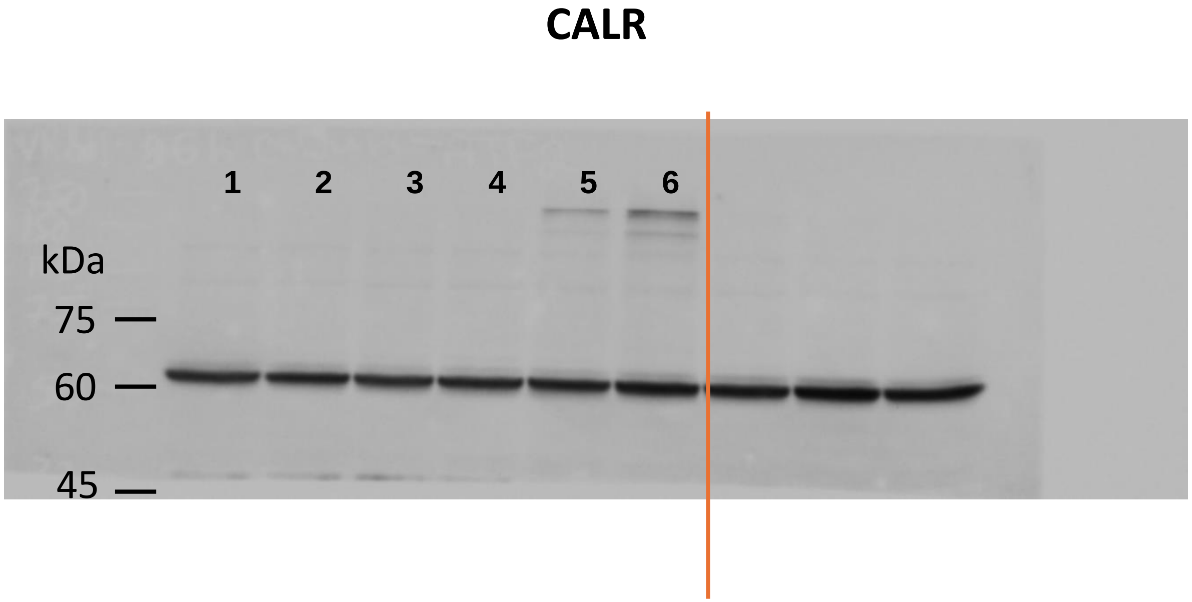

1- Control
2- $1,25D_3$
3- CA
4- $1,25D_3$+CA
5- MMF
6- $1,25D_3$+MMF
