# Supplementary material for: Roles of Glutathione and AP-1 in the Enhancement of Vitamin D-Induced Differentiation by Activators of the Nrf2 Signaling Pathway in Acute Myeloid Leukemia Cells
Source: Int J Mol Sci. 2024 Feb 14;25(4):2284. doi: 10.3390/ijms25042284 (PMC10889780; doi:10.3390/ijms25042284)
Supplement: Supplementary file 1 [file ijms-25-02284-s001.zip › Supplementary Figures S1-S3-ijms-2024.pdf]

International Journal of
Molecular Sciences

MDPI

Supplementaty Materials

Roles of Glutathione and AP-1 in the Enhancement of Vitamin D-induced Differentiation by Activators of the Nrf2 Signaling Pathway in Acute Myeloid Leukemia Cells

Yasmeen Jramne-Saleem and Michael Danilenko *

Department of Clinical Biochemistry and Pharmacology, Faculty of Health Sciences, Ben-Gurion University of the Negev, Beer Sheva 8410501, Israel; jramne@post.bgu.ac.il

* Correspondence: misha@bgu.ac.il; Tel.: +972-8-647-9979

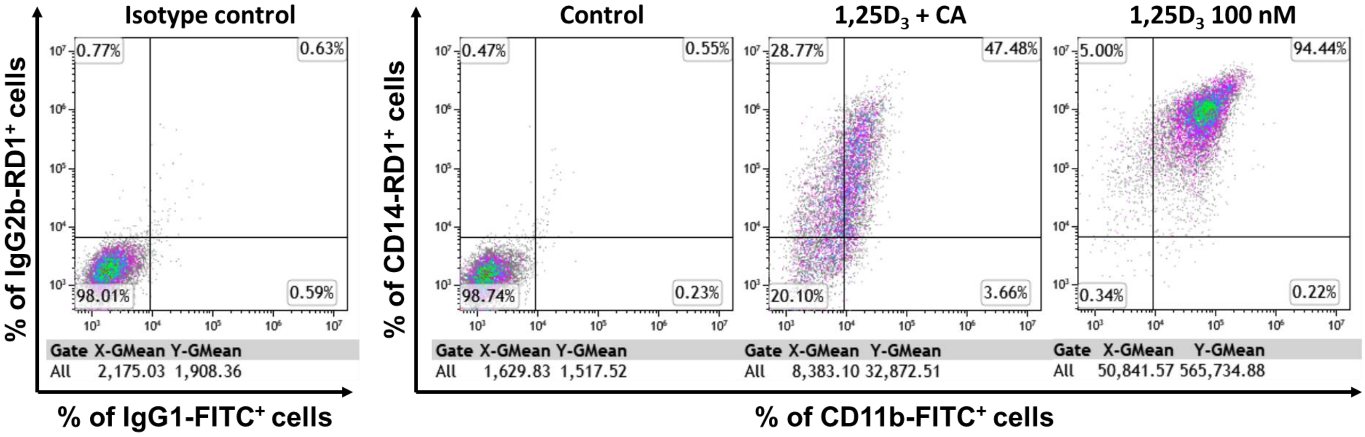

Supplementary Figure S1. Typical analysis of cell surface CD14 and CD11b expression in HL60 cells by bivariate flow cytometry. Cells were incubated with vehicle (0.2% ethanol; Control), the combination of 1 nM $1,25D_3$ and 10 μM carnosic acid (CA) or a high concentration of $1,25D_3$ (100 nM; Positive control), for 48 h. Following treatments, cells were washed and incubated with fluorescent antibodies followed by flow cytometric analysis, as described in the Materials and Methods. The vehicle-treated cell sample was divided into two parts. One part was stained with isotype control antibodies (IgG2b-RD1 for CD14-RD1 and IgG1-FITC for CD11b-FITC). The other part as well as the rest of the samples were stained with CD14-RD1 and CD11b-FITC antibodies. .

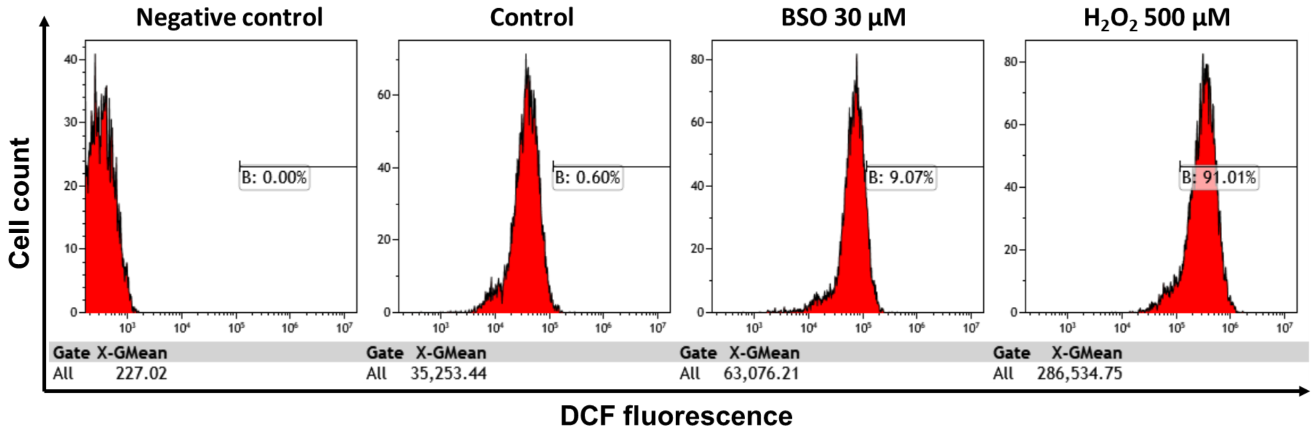

Supplementary Figure S2. Typical analysis of cytosolic ROS levels in HL60 cells by flow cytometry. Cells were incubated with vehicle (0.2% ethanol; Control) or 30 μM BSO for 72 h. Following incubations, the control sample was divided into three parts. One part was put aside and used as a negative

control. The rest of the control and BSO-treated samples were loaded with DCFH-DA. One of the DCFH-DA-loaded control samples was treated with 500 μM H_2O_2 for 15 min and used as a positive control. All samples were then subjected to flow cytometric analysis, as described in Materials and Methods.

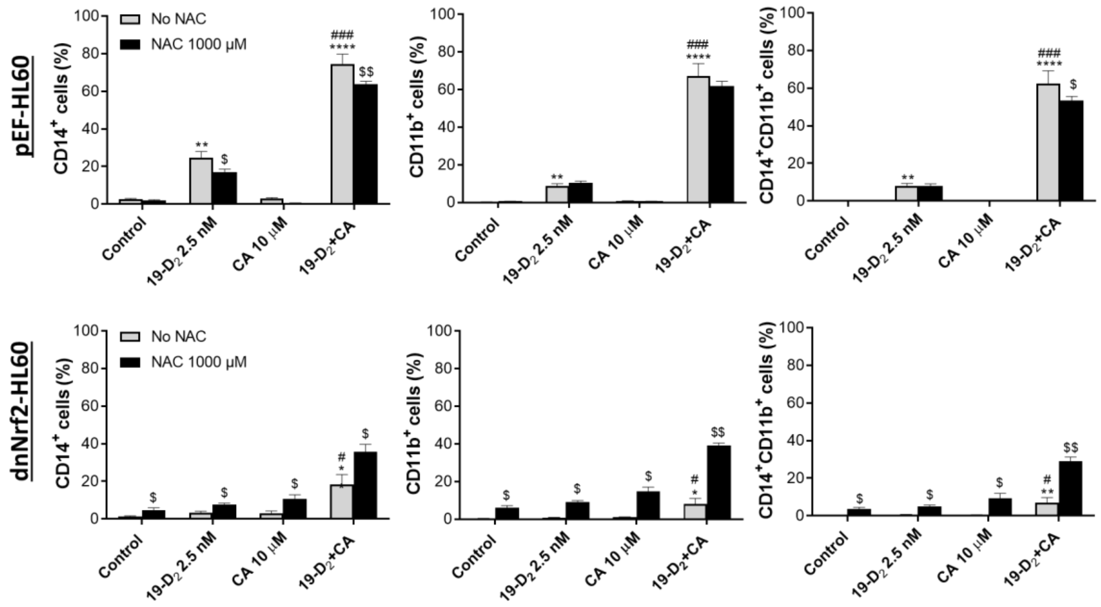

Supplementary Figure S3. N-acetylcysteine partially reverses the inhibitory effect of dominant-negative Nrf2 on the differentiation of HL60 cells. (a,b) pEF-HL60 and dnNrf2-HL60 cells were pre-incubated with vehicle or 1000 μM N-acetylcysteine (NAC) for 1 h, followed by incubating with the indicated concentrations of paricalcitol, CA, or their combination for another 48 h. The expression of CD14 and CD11b was determined by flow cytometry. The data are means ± SD of 3 experiments *, $p < 0.05$; **, $p < 0.01$; ****; $p < 0.0001$, vs. corresponding untreated control group; #, $p < 0.05$; ###, $p <$ 0.001; ####, $p < 0.0001$ vs. corresponding sum of the effects of single agents; $, $p < 0.05$; $$, NAC-treated vs. corresponding NAC-untreated group.
